# Supplementary material for: Systematic review and meta-analysis of cohort studies of long term outdoor nitrogen dioxide exposure and mortality
Source: PLoS One. 2021 Feb 4;16(2):e0246451. doi: 10.1371/journal.pone.0246451 (PMC7861378; doi:10.1371/journal.pone.0246451)
Supplement: S1 Fig — (PDF) [file pone.0246451.s001.pdf]

Online supplementary figure S1. Risk of bias criteria adapted from Lam J, Sutton P, Kalkbrenner A, et al. A Systematic Review and Meta-Analysis of Multiple Airborne Pollutants and Autism Spectrum Disorder. PLoS One. 2016;11(9):e0161851. Published 2016 Sep 21. doi:10.1371/journal.pone.0161851

# Selection Bias/ Generalizability

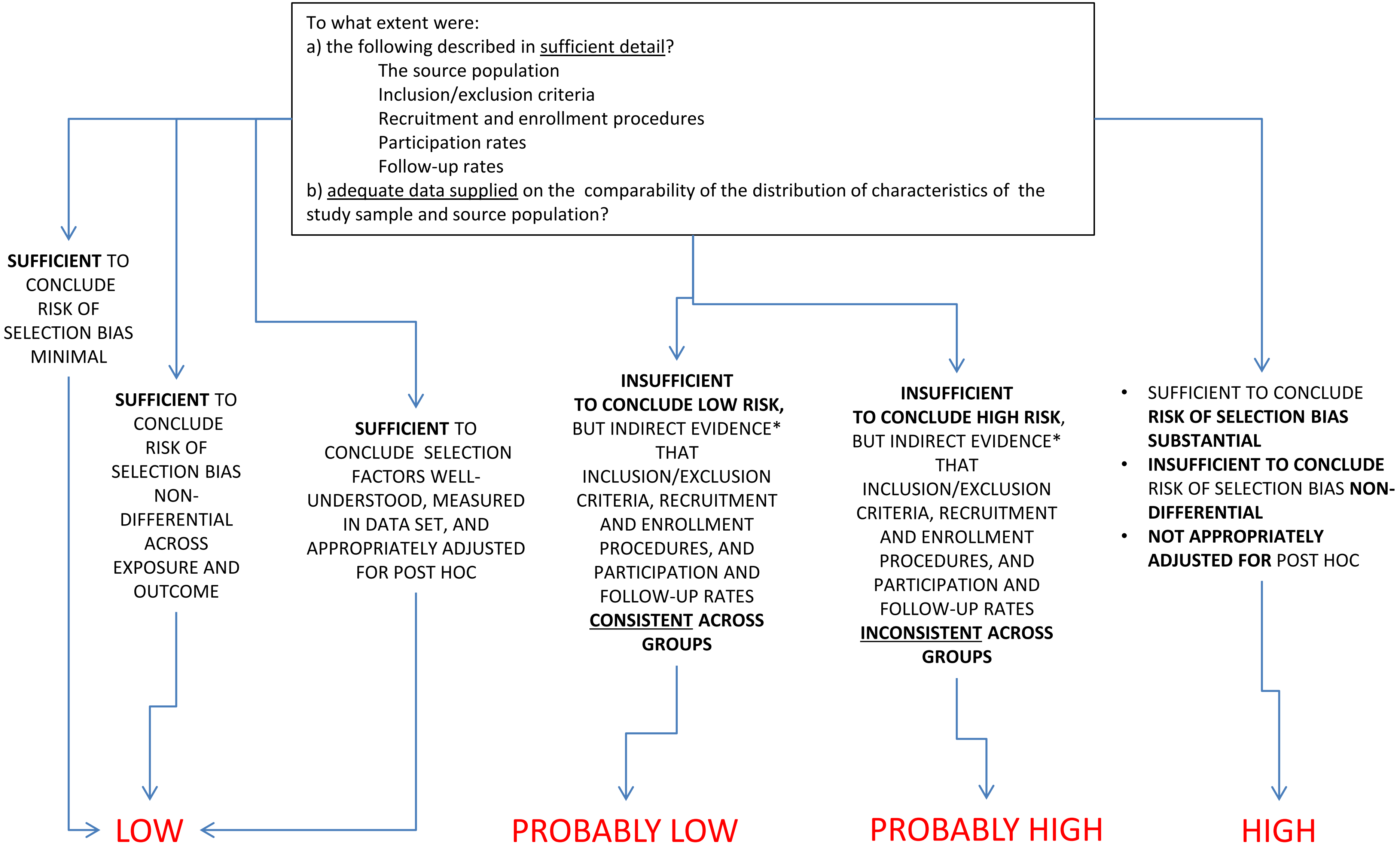


*e.g. groups expected to be similar/different based on selection procedure

# Exposure Assessment - Modeling

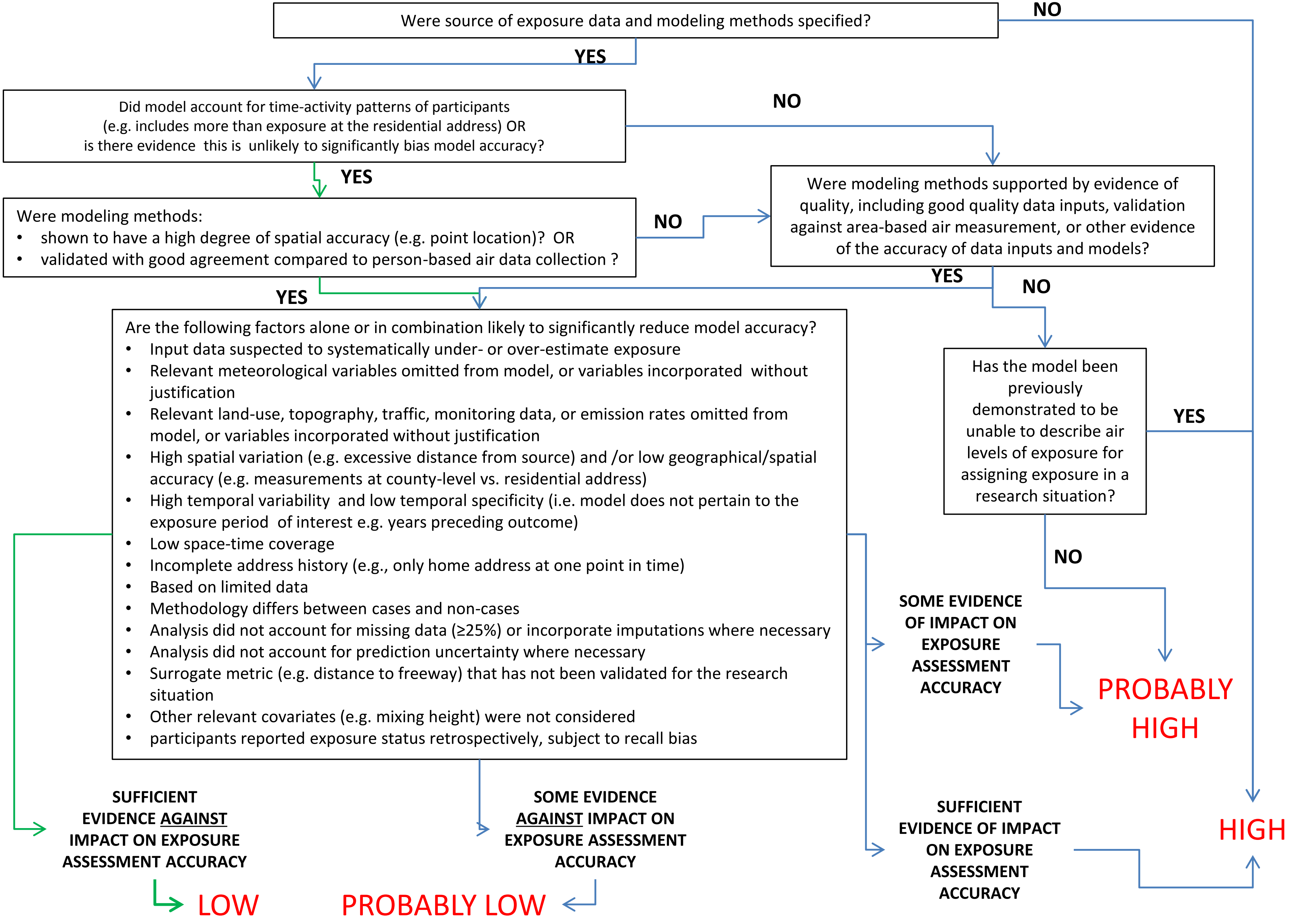

# Exposure Assessment - Monitoring

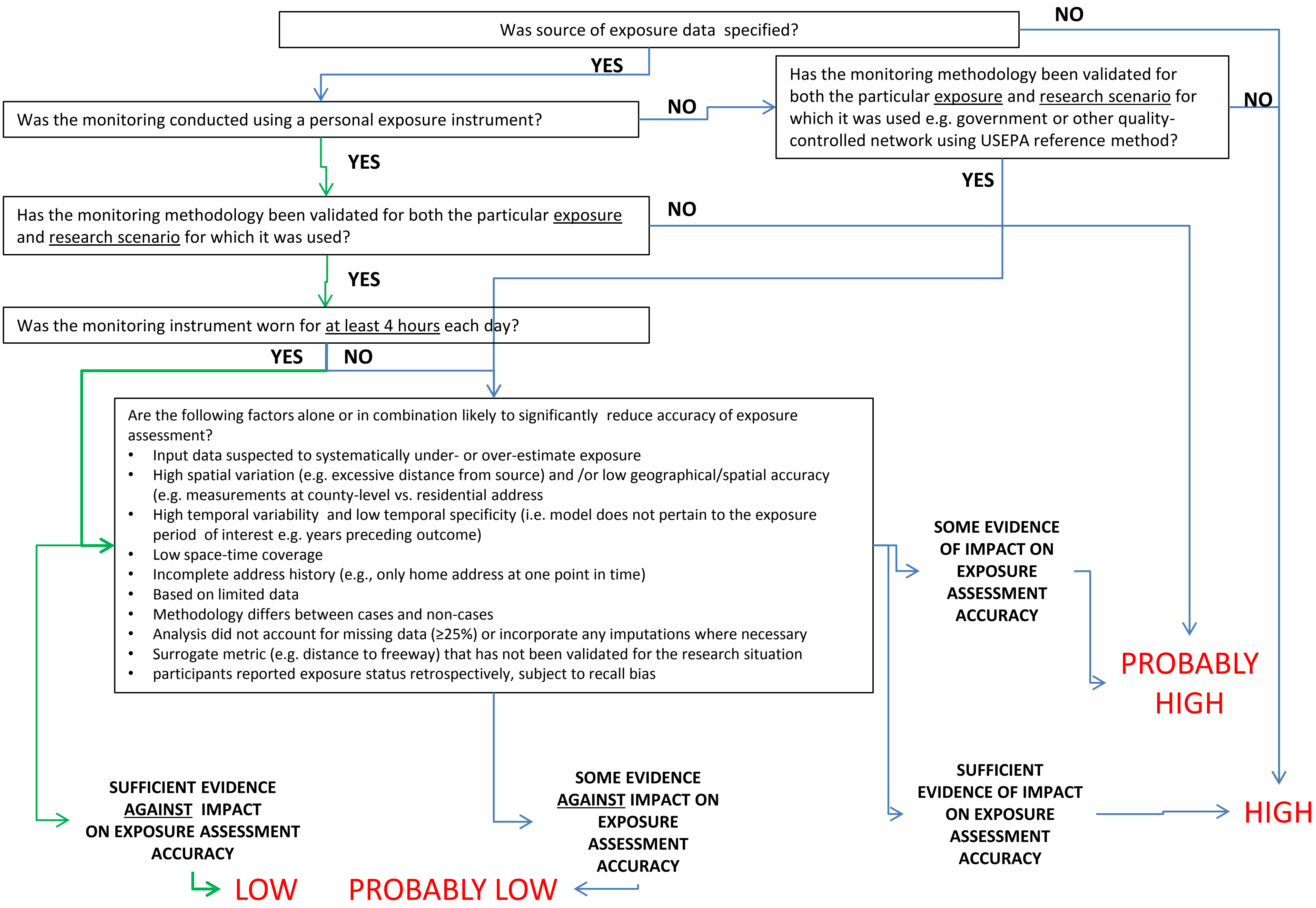

# Confounding

To what extent did the study measure <u>important</u> potential confounders consistently across study groups using valid and reliable methods and account for them in design or analysis:

- **<u>Age</u>**
- **<u>Sex</u>**
- **<u>Smoking</u>**
- Alcohol consumption
- Race
- Diet
- Medication
- Comorbidity
- Obesity (BMI)
- Education
- Occupation
- Income
- **<u>Individual or Neighborhood SES</u>**
- Spatial autocorrelation
- Greenness, noise

<u>OR</u> did the study report that potential confounders were <u>evaluated and omitted</u> because inclusion did not substantially affect the results?

**ALL IMPORTANT CONFOUNDERS**
**e.g. includes all of age, sex, smoking, SES**

LOW

**MOST IMPORTANT CONFOUNDERS AND THIS IS NOT EXPECTED TO INTRODUCE SUBSTANTIAL BIAS**
**e.g. indirect adjustment for omitted covariates**

PROBABLY LOW

**SOME BUT NOT ALL IMPORTANT CONFOUNDERS AND THIS IS EXPECTED TO INTRODUCE SUBSTANTIAL BIAS**
**e.g. missing any 1 of age, sex, smoking, SES**

PROBABLY HIGH

**DID NOT ACCOUNT FOR OR EVALUATE MULTIPLE IMPORTANT CONFOUNDERS**
**e.g. missing 2 or more of age, sex, smoking, SES**

HIGH

# Outcome Assessment

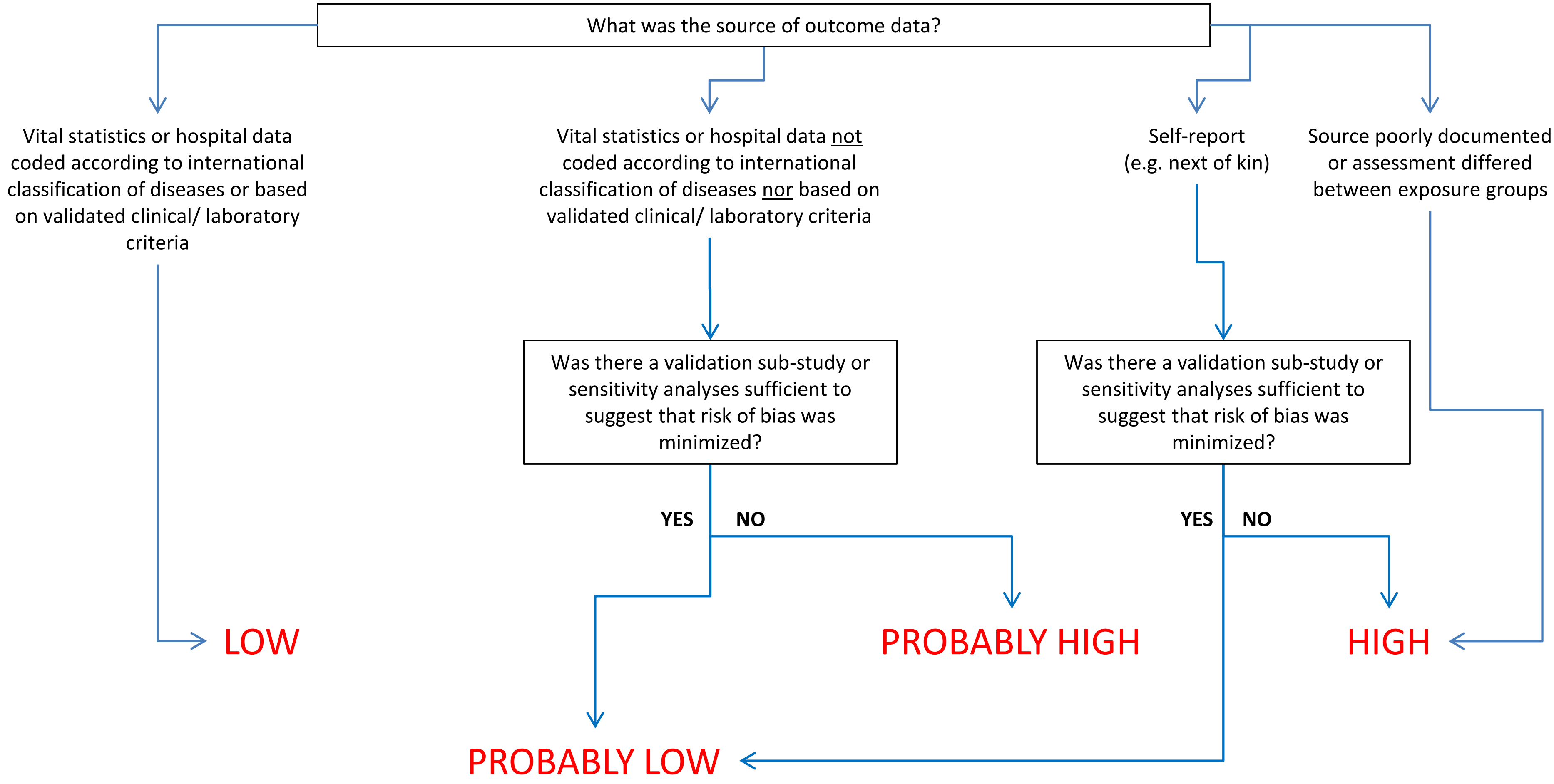

## Completeness of Outcome Data

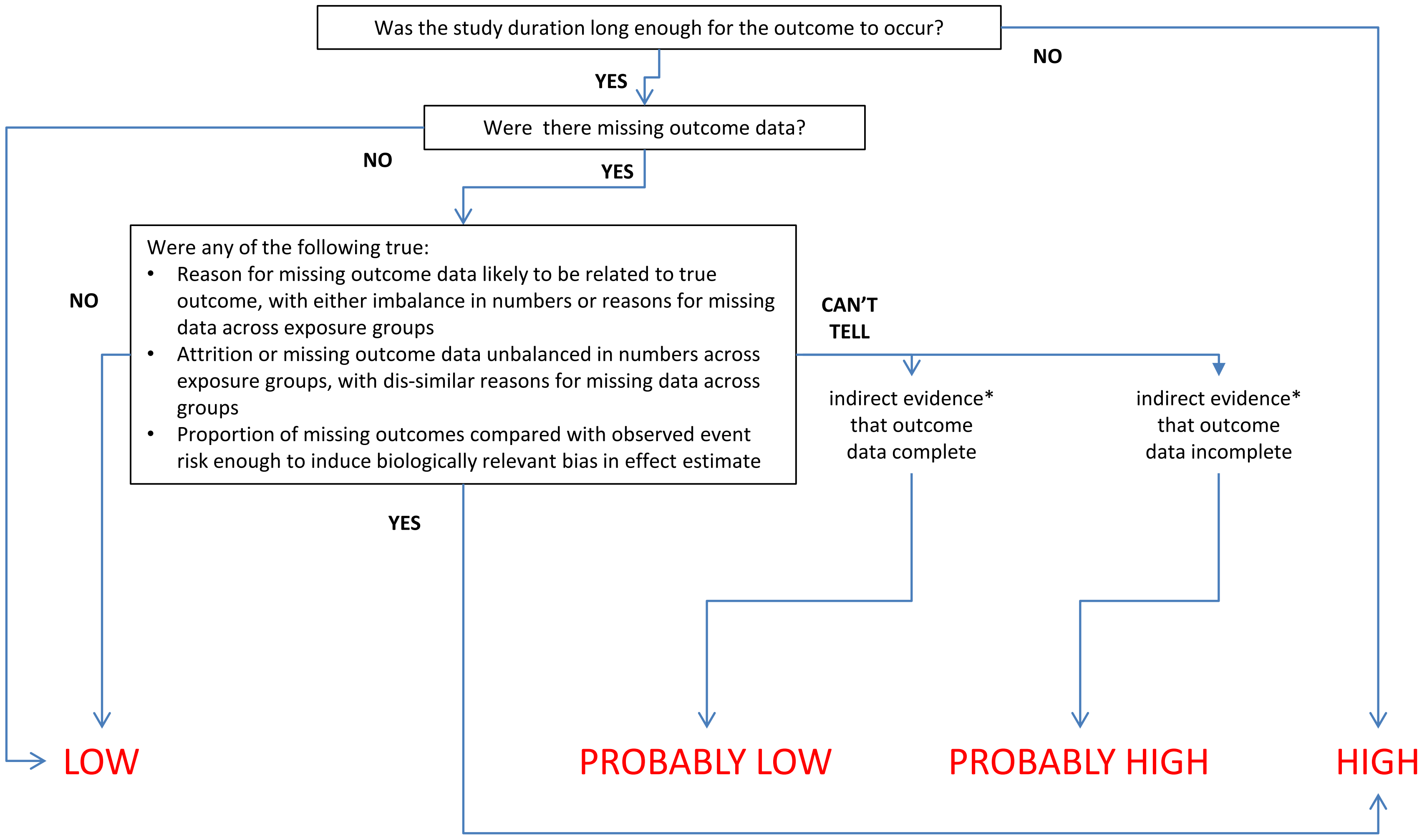


*e.g. based on study design

# Selective Outcome Reporting

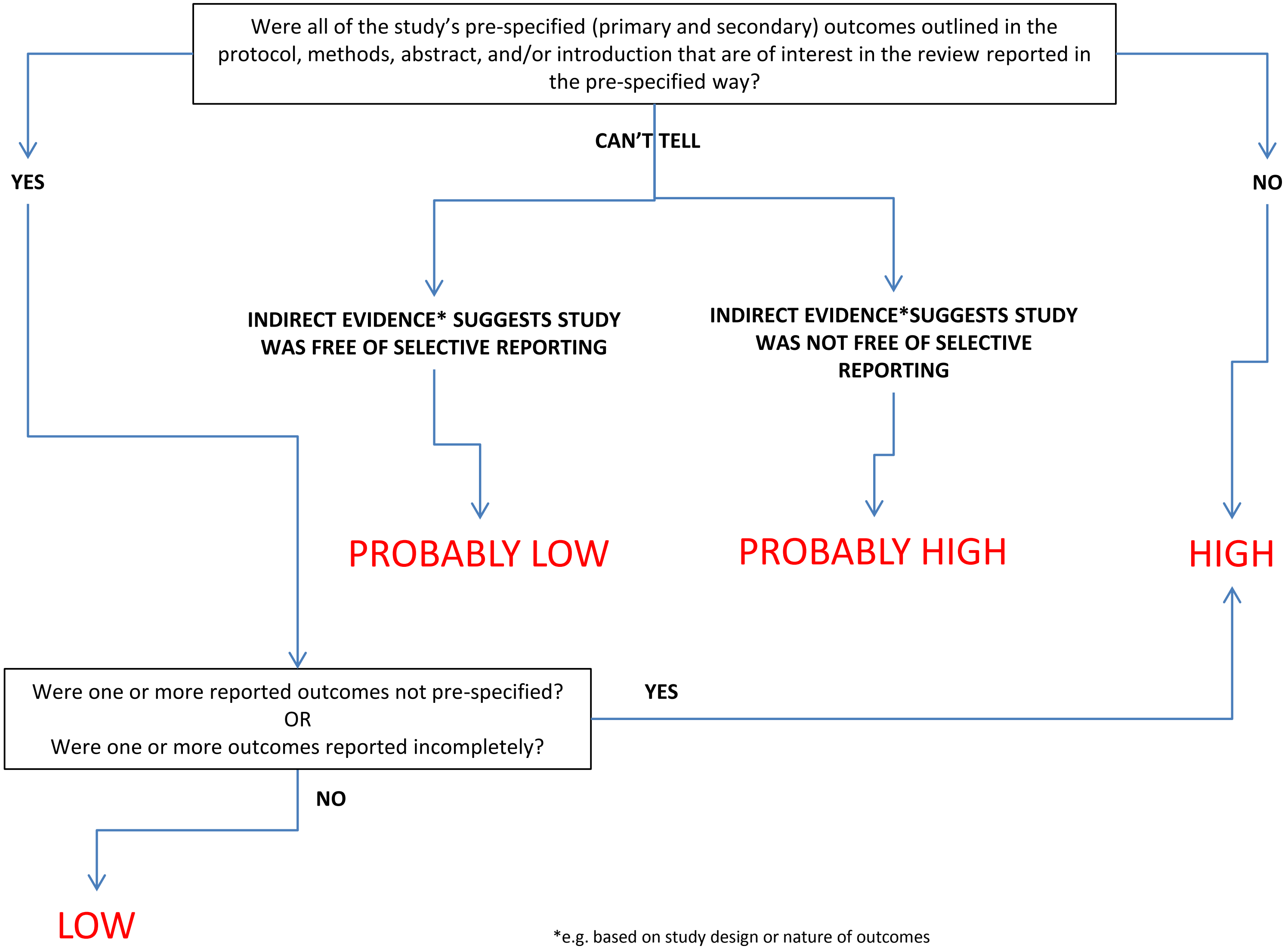


*e.g. based on study design or nature of outcomes

# Conflict of Interest

Is it clearly established that the study did not receive support from a company, study author, or other entity having a financial interest in the outcome of the study. Examples include the following:

- Funding source is limited to government, non-profit organizations, or academic grants funded by government, foundations and/or non-profit organizations;
- Chemicals or other treatment used in study were purchased from a supplier;
- Company affiliated staff are not mentioned in the acknowledgements section;
- Authors were not employees of a company with a financial interest in the outcome of the study;
- Company with a financial interest in the outcome of the study was not involved in the design, conduct, analysis, or reporting of the study and authors had complete access to the data;
- Study authors make a claim denying conflicts of interest;
- Study authors are unaffiliated with companies with financial interest, and there is no reason to believe a conflict of interest exists;
- All study authors are affiliated with a government agency (are prohibited from involvement in projects for which there is a conflict of interest or an appearance of conflict of interest).?

**YES** → LOW

**NO** →

Did the study receive support from a company, study author, or other entity having a financial interest in the outcome of the study. Examples of support include:

- Research funds;
- Chemicals, equipment or testing provided at no cost;
- Writing services;
- Author/staff from study was employee or otherwise affiliated with company with financial interest;
- Company limited author access to the data;
- Company was involved in the design, conduct, analysis, or reporting of the study;
- Study authors claim a conflict of interest

**YES** → HIGH

**NO OR CAN'T TELL** →

**INDIRECT EVIDENCE* SUGGESTS STUDY WAS FREE OF CONFLICT** → PROBABLY LOW

**INDIRECT EVIDENCE* SUGGESTS STUDY WAS NOT FREE OF CONFLICT** → PROBABLY HIGH

*reviewer judgement

# Other Sources of Bias

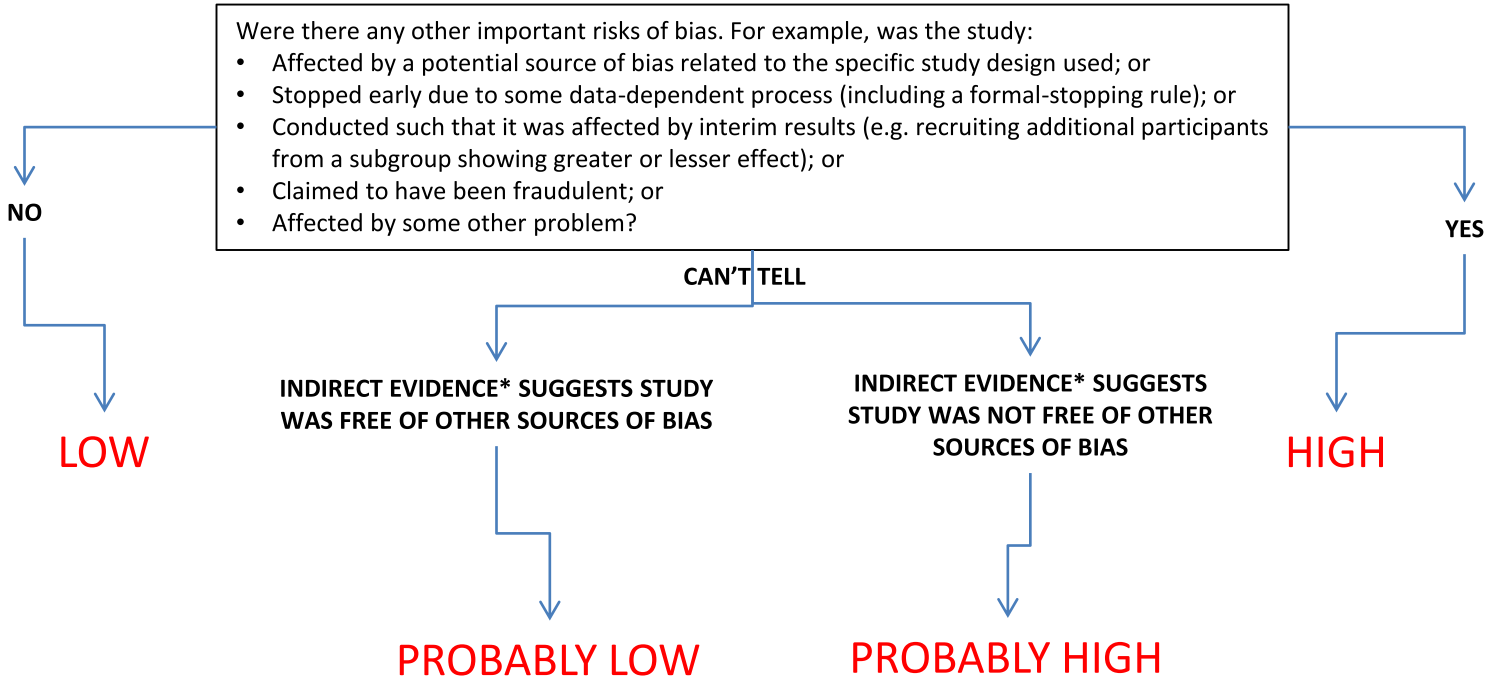


*reviewer judgement
