# Supplementary material for: Systematic review and meta-analysis of cohort studies of long term outdoor nitrogen dioxide exposure and mortality
Source: PLoS One. 2021 Feb 4;16(2):e0246451. doi: 10.1371/journal.pone.0246451 (PMC7861378; doi:10.1371/journal.pone.0246451)
Supplement: S2 Fig — (PDF) [file pone.0246451.s002.pdf]

Online supplementary figure S2 . Risk of bias ratings for individual studies

Study [reference]	Region	Cohort	Selection Bias	Exposure Assessment	Confounding	Outcome Assessment	Completeness of Outcome Data	Selective Outcome Reporting	Conflict of Interest	Other
Crouse 2015 [1]	Canada	CanCHEC 1991	Low	Probably Low	Probably Low	Low	Low	Low	Low	Low
Crouse 2015 [2]	Canada	CanCHEC 1991 (10 cities)	Low	Probably Low	Probably Low	Low	Low	Low	Low	Low
Weichenthal 2017 [3]	Canada	CanCHEC 2001	Low	Probably Low	Probably High	Low	Low	Low	Low	Low
Villeneuve 2013 [4]	Canada	OTCS	Low	Probably High	Probably Low	Low	Low	Low	Low	Low
Chen 2013 [5]	Canada	OTCS	Low	Probably Low	Probably Low	Low	Low	Low	Low	Low
Jerrett 2009 [6]	Canada	Toronto Western Hospital	Low	Probably High	Low	Low	Low	Low	Low	Low
Gan 2011 [7]	Canada	Vancouver CHD	Low	Probably Low	Probably Low	Low	Low	Low	Low	Low
Gan 2013 [8]	Canada	Vancouver COPD	Low	Probably Low	Probably Low	Low	Low	Low	Low	Low
Abbey 1999 [9]	United States	AHSMOG	Probably Low	Probably High	Low	Low	Low	Low	Low	Low
Chen 2005 [10]	United States	AHSMOG	Probably Low	Probably High	Low	Low	Low	Low	Low	Low
Eckel 2016 [11]	United States	California cancer	Low	Probably High	Probably High	Low	Low	Low	Low	Low
Hartiala 2016 [12]	United States	Cleveland Clinic GeneBank	Probably Low	Probably High	Low	Probably Low	Low	Low	Low	Low
Jerrett 2013 [13]	United States	CPS II	Probably Low	Probably High	Low	Low	Low	Low	Low	Low
Pope 2002 [14]	United States	CPS II	Probably Low	Probably High	Low	Low	Low	Low	Low	Low
Krewski 2009 [15]	United States	CPS II	Probably Low	Probably High	Low	Low	Low	Low	Low	Low
McKean-Cowdin 2009 [16]	United States	CPS II	Probably Low	Probably High	Low	Low	Low	Low	Low	Low
Turner 2017 [17]	United States	CPS II	Probably Low	Probably High	Low	Low	Low	Low	Low	Low
Turner 2016 [18]	United States	CPS II	Probably Low	Probably High	Low	Low	Low	Low	Low	Low
Lipsett 2011 [19]	United States	CTS	Probably Low	Probably Low	Low	Low	Low	Low	Low	Low
Krewski 2000 [20]	United States	Harvard Six Cities	Probably Low	Probably Low	Low	Low	Low	Low	Low	Low
Eum 2019 [21]	United States	Medicare	Low	Probably High	Probably High	Low	Low	Low	Probably Low	Low
Lefler 2019 [22]	United States	NHIS	Probably Low	Probably High	Low	Low	Low	Low	Low	Low
Hart 2013 [23]	United States	NHS	Probably Low	Probably Low	Low	Low	Low	Low	Low	Low
Lim 2019 [24]	United States	NIH-AARP	Probably Low	Probably High	Low	Low	Low	Low	Low	Low
Lim 2019 [25]	United States	NIH-AARP	Probably Low	Probably High	Low	Low	Low	Low	Low	Low
Lim 2018 [26]	United States	NIH-AARP	Probably Low	Probably High	Low	Low	Low	Low	Low	Low
Hart 2011 [27]	United States	TrIPS	Probably Low	Probably High	High	Low	Low	Low	Low	Low
Lipfert 2006 [28]	United States	WU/EPRI Veterans	Probably Low	Probably High	Low	Low	Low	Low	Probably Low	Low
Lipfert 2006 [29]	United States	WU/EPRI Veterans	Probably Low	Probably High	Low	Low	Low	Low	Probably Low	Low
Lipfert 2009 [30]	United States	WU/EPRI Veterans	Probably Low	Unable to assess	Low	Low	Low	Low	Probably Low	Low
Lipfert 2019 [31]	United States	WU/EPRI Veterans	Probably Low	Probably High	Low	Low	Low	Low	Probably Low	Low
Lipfert 2018 [32]	United States	WU/EPRI Veterans	Probably Low	Probably High	Low	Low	Low	Low	Probably Low	Low
Bauleo 2019 [33]	Europe	Civitavecchia	Low	Unable to assess	Probably High	Low	Low	Low	Low	Low
Carey 2013 [34]	Europe	CPRD	Probably Low	Probably High	Low	Low	Low	Low	Low	Low
Sifaki-Pistolla 2017 [35]	Europe	Crete lung cancer	Low	Probably High	Probably High	Low	Low	Low	Low	Low
Raaschou-Nielsen 2012 [36]	Europe	DDCH	Probably Low	Probably Low	Low	Low	Low	Low	Low	Low
Raaschou-Nielsen 2012 [37]	Europe	DDCH	Probably Low	Probably Low	Low	Low	Low	Low	Low	Low
Hvitfeldt 2019 [38]	Europe	DDCH	Probably Low	Probably Low	Low	Low	Low	Low	Low	Low
Sørensen 2014 [39]	Europe	DDCH	Probably Low	Probably Low	Low	Low	Low	Low	Low	Low
Andersen 2011 [40]	Europe	DDCH	Probably Low	Probably Low	Low	Low	Low	Low	Low	Low
Fischer 2015 [41]	Europe	DUELS	Low	Probably High	Probably Low	Low	Low	Low	Low	Low
Dimakopoulou 2014 [42]	Europe	ESCAPE	Probably Low	Probably Low	Low	Low	Low	Low	Low	Low
Beelen 2014 [43]	Europe	ESCAPE	Probably Low	Probably Low	Low	Low	Low	Low	Low	Low
Beelen 2014 [44]	Europe	ESCAPE	Probably Low	Probably Low	Low	Low	Low	Low	Low	Low
Bentayeb 2015 [45]	Europe	GAZEL	Probably Low	Probably Low	Low	Low	Low	Low	Low	Low
Tonne 2013 [46]	Europe	MINAP	Low	Probably Low	Low	Low	Low	Low	Low	Low
Tonne 2016 [47]	Europe	MINAP	Low	Probably Low	Low	Low	Low	Low	Low	Low
Stockfelt 2015 [48]	Europe	MPPS	Probably Low	Probably Low	Low	Low	Low	Low	Low	Low
Dehbi 2017 [49]	Europe	NHSD, SABRE	Probably Low	Probably High	Low	Low	Low	Low	Low	Low
Beelen 2008 [50]	Europe	NLCS	Probably Low	Probably Low	Low	Low	Low	Low	Low	Low
Beelen 2008 [51]	Europe	NLCS	Probably Low	Probably Low	Low	Low	Low	Low	Low	Low
Brunekreef 2009 [52]	Europe	NLCS	Probably Low	Probably Low	Low	Low	Low	Low	Low	Low
Hoek 2002 [53]	Europe	NLCS	Probably Low	Probably Low	Low	Low	Low	Low	Low	Low
Nafstad 2004 [54]	Europe	Norwegian men	Probably Low	Probably Low	Low	Low	Low	Low	Low	Low
Naess 2007 [55]	Europe	Oslo	Low	Probably Low	Probably Low	Low	Low	Low	Low	Low
Filleul 2005 [56]	Europe	PAARC	Probably Low	Probably High	Low	Low	Low	Low	Low	Low
Klompmaker 2020 [57]	Europe	PHM	Probably Low	Probably High	Low	Low	Low	Low	Low	Low
Cesaroni 2012 [58]	Europe	RoLS	Low	Probably Low	Probably High	Low	Low	Low	Low	Low
Cesaroni 2013 [59]	Europe	RoLS	Low	Probably Low	Probably Low	Low	Low	Low	Low	Low
Rosenlund 2008 [60]	Europe	Rome	Low	Probably High	Probably Low	Low	Low	Low	Low	Low
Schikowski 2007 [61]	Europe	SALIA	Probably Low	Probably High	Low	Low	Low	Low	Low	Low
Gehring 2006 [62]	Europe	SALIA	Probably Low	Probably High	Low	Low	Low	Low	Low	Low
Heinrich 2013 [63]	Europe	SALIA	Probably Low	Probably High	Low	Low	Low	Low	Low	Low
Nieuwenhuijsen 2018 [64]	Europe	SIDIAP	Low	Probably Low	Low	Low	Low	Low	Low	Low
Maheswaran 2010 [65]	Europe	SLSR	Probably Low	Probably Low	Low	Low	Low	Low	Low	Low
Desikan 2016 [66]	Europe	SLSR	Probably Low	Probably Low	Probably High	Low	Low	Low	Low	Low
Heritier 2019 [67]	Europe	SNC	Low	Probably High	Probably High	Low	Low	Low	Low	Low
Cao 2011 [68]	Other	CNHS	Probably Low	Probably High	Low	Low	Low	Low	Low	Low
Dirgawati 2019 [69]	Other	HIMS	Probably Low	Probably Low	Low	Low	Low	Low	Low	Low
Yang 2018 [70]	Other	HKEHC	Probably Low	Probably High	Low	Low	Low	Low	Low	Low
Barratt 2018 [71]	Other	HKEHC	Probably Low	Low	Low	Low	Low	Low	Low	Low
Kim 2017 [72]	Other	NHIS-NSC	Low	Probably High	Probably High	Low	Low	Low	Low	Low
Chen 2016 [73]	Other	Northern Chinese	Probably Low	Probably Low	Low	Low	Probably Low	Low	Low	Low
Yorifuji 2010 [74]	Other	SEC	Probably Low	Probably High	Low	Low	Probably High	Low	Low	Low
Yorifuji 2013 [75]	Other	SEC	Probably Low	Probably High	Low	Low	High	Low	Low	Low
Dong 2012 [76]	Other	Shenyang	Probably Low	Probably High	Probably High	Low	Low	Low	Low	Low
Zhang 2011 [77]	Other	Shenyang	Probably Low	Probably High	Probably High	Low	Low	Low	Low	Low
Tseng 2015 [78]	Other	Taiwan civil servants	Probably Low	Probably High	Low	Low	Low	Low	Low	Low
Katanoda 2011 [79]	Other	Three Prefecture	Probably Low	Probably High	Low	Low	Low	Low	Low	Low

Risk of Bias

Low | Probably Low | Probably High | High | Unable to assess

Cohort abbreviations: AHSMOG, Adventist Health and smog; CanCHEC, Canadian Census Health and Environment Cohort; CPS-II, Cancer Prevention Study-II; CNHS, China National Hypertension Survey; CPRD, Clinical Practice Research Datalink; CTS, California Teachers Study; DDCH, Danish Diet, Cancer and Health; DUELS, Dutch Environmental Longitudinal Study; ESCAPE, European Study of Cohorts for Air Pollution Effects; GAZEL, GAZ and ELectricité; HIMS, Health in Men Study; HKEHC, Hong Kong Elderly Health Centres; JPHC, Japan Public Health Centre; MINAP, Myocardial Ischaemia National Audit Project; MPPS, Multifactor Primary Prevention Study; NHIS, National Health Interview Survey; NHIS-NSC, National Health Insurance Service–National Sample Cohort; NIH-AARP, National Institutes of Health, American Association of Retired Persons; NSHD, National Survey of Health and Development; NLCS, Netherlands Cohort study on Diet and Cancer; NHS, Nurses Health Study; OTCS, Ontario Tax Cohort study; PAARC, Pollution Atmosphérique et Affections Respiratoires Chroniques; PHM, Public Health Monitor; RoLS, Rome Longitudinal Study; SEC, Shizuoka elderly cohort; SIDIAP, Sistema d'Informació pel Desenvolupament de la Investigació en Atenció Primària; SLSR, South London Stroke Register; SABRE, Southall And Brent Revisited; SALIA, Study on the influence of Air pollution on Lung function, Inflammation and Aging; SNC, Swiss National Cohort; TRiPS, the Trucking Industry Particle Study; WU/EPRI, Washington University/Electric Power Research Institute.
