# Supplementary material for: Systematic review and meta-analysis of cohort studies of long term outdoor nitrogen dioxide exposure and mortality
Source: PLoS One. 2021 Feb 4;16(2):e0246451. doi: 10.1371/journal.pone.0246451 (PMC7861378; doi:10.1371/journal.pone.0246451)
Supplement: S3 Fig — (PDF) [file pone.0246451.s003.pdf]

Online supplementary figure S3. Forest plots by region

A) Canada

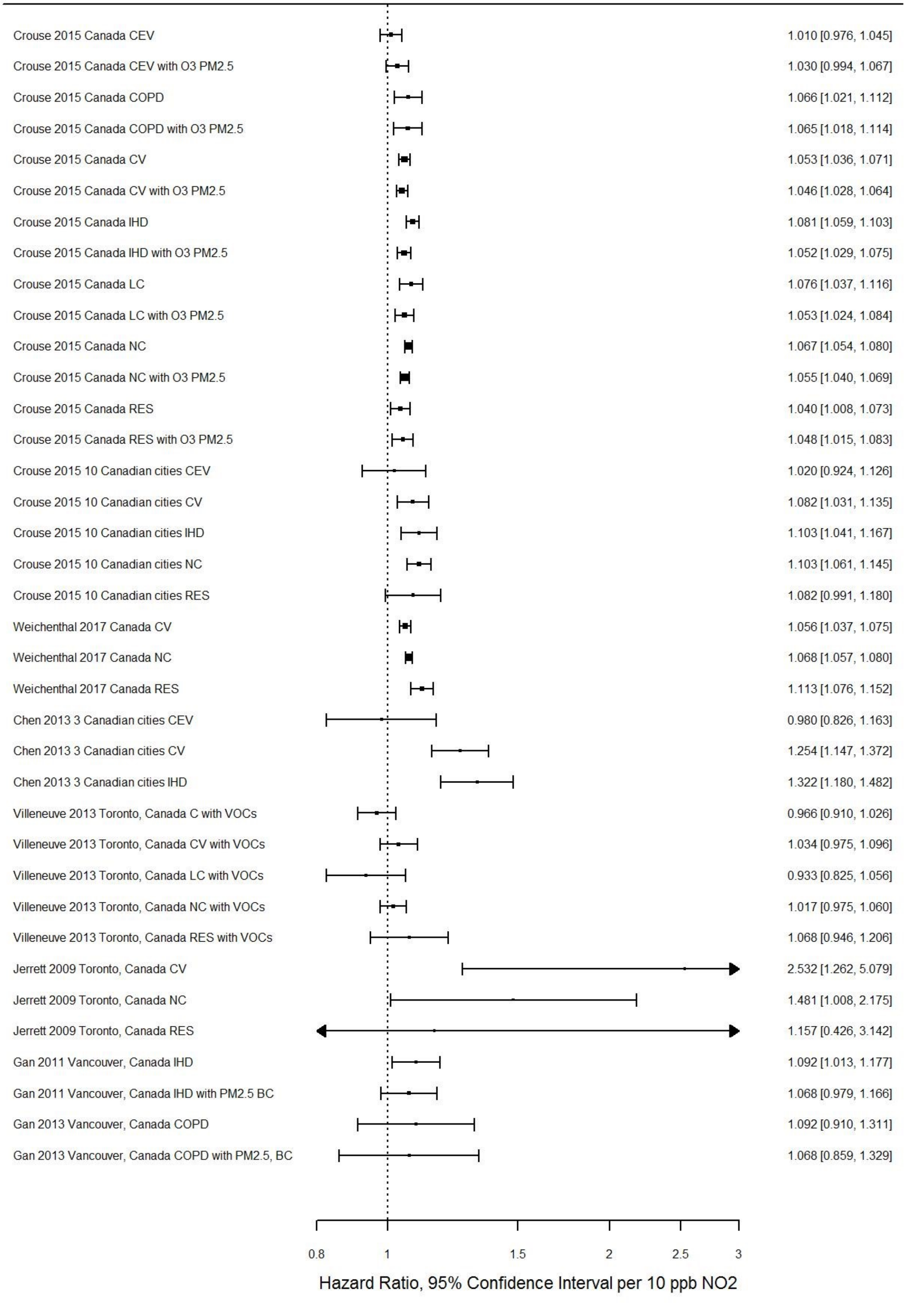

B) United States – All/natural cause

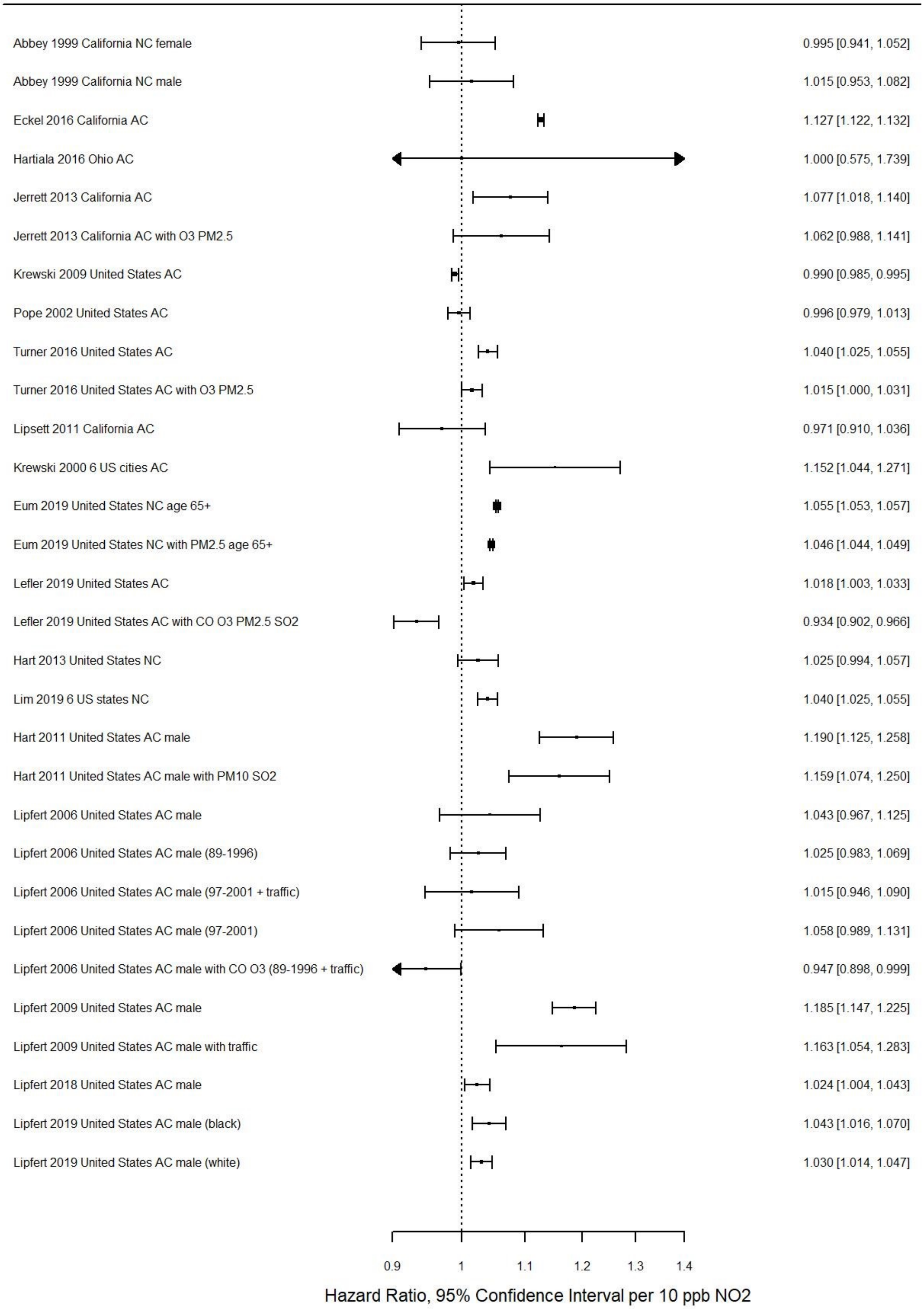

C) United States – Specific causes

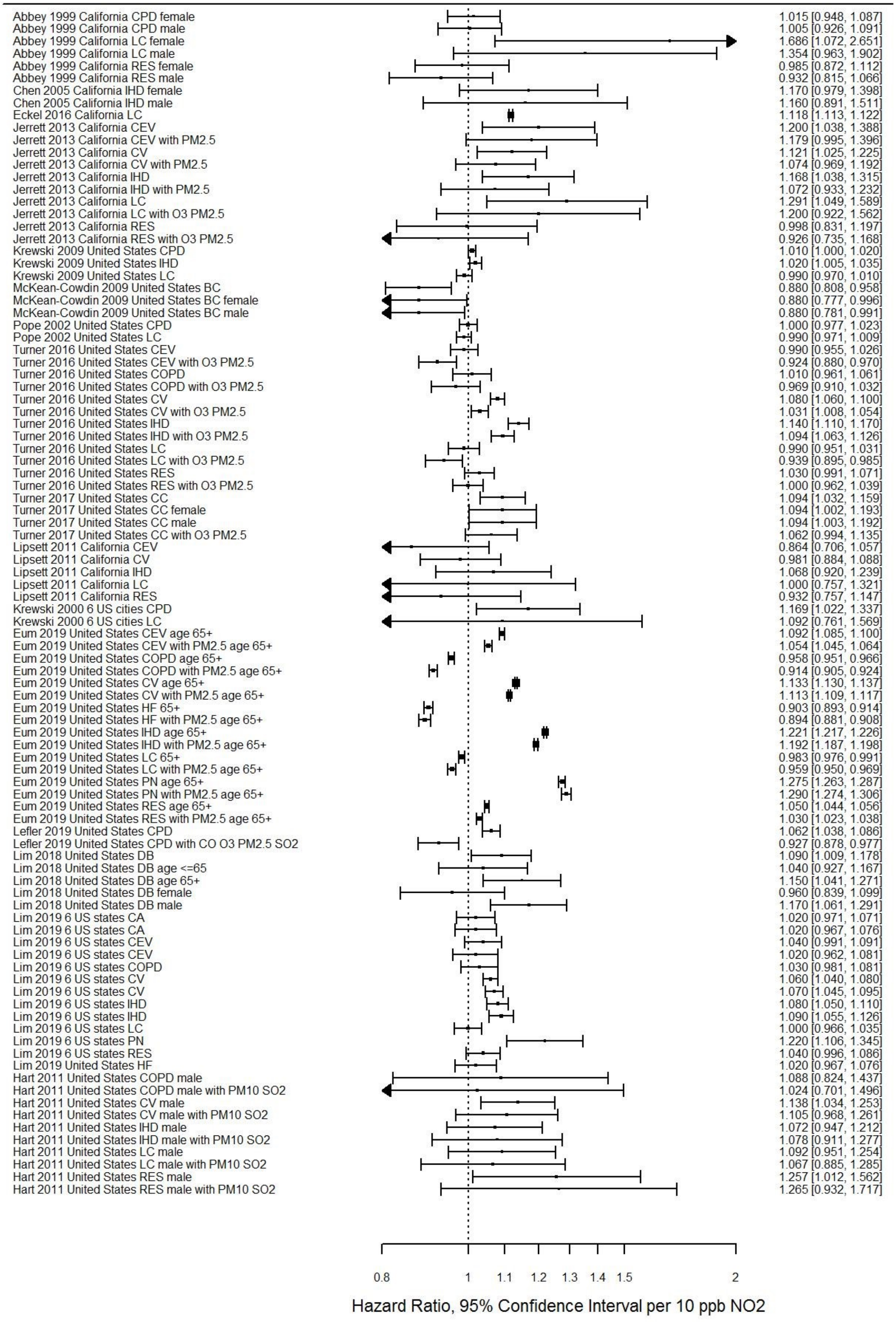

D) Europe – All/natural cause

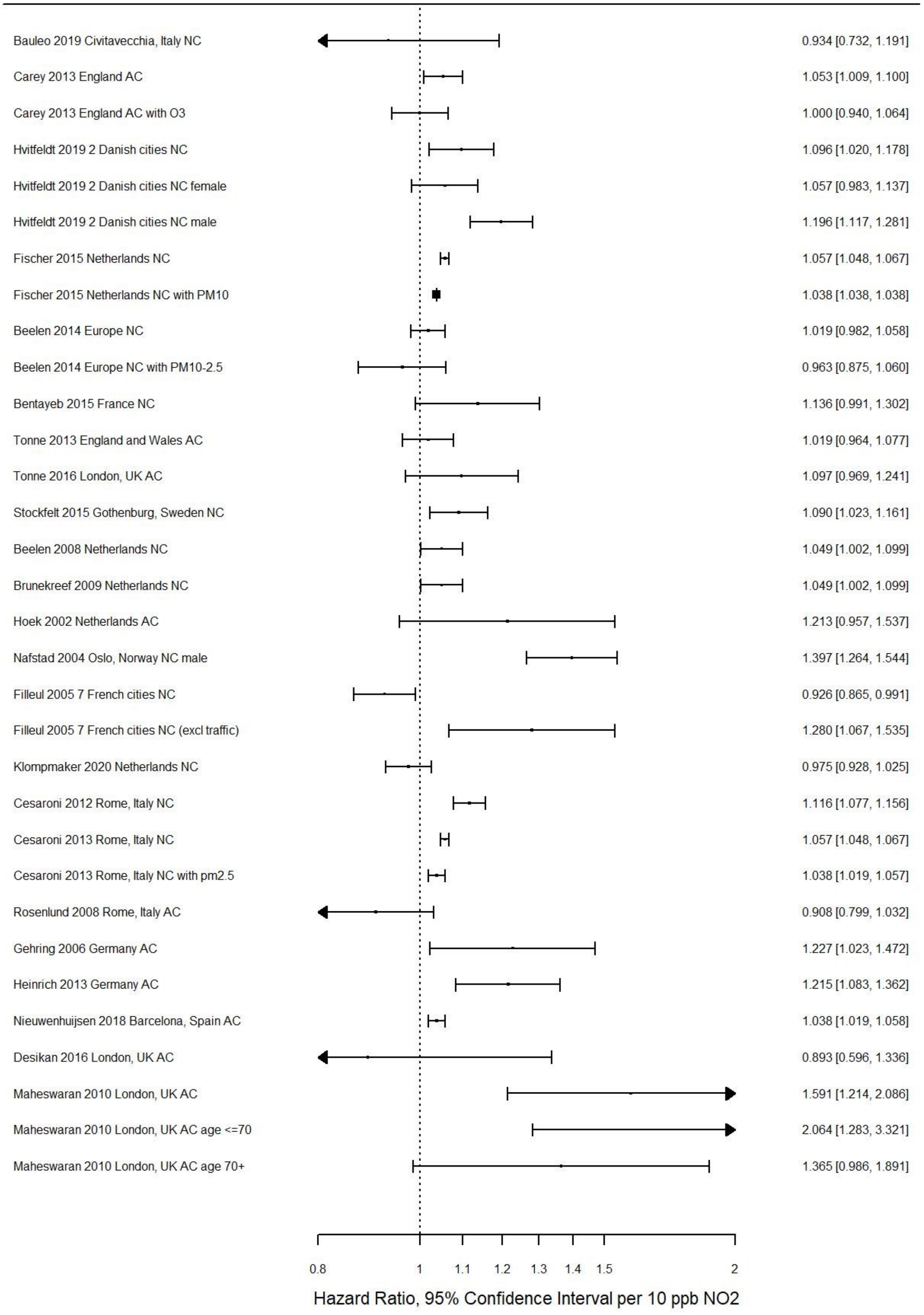

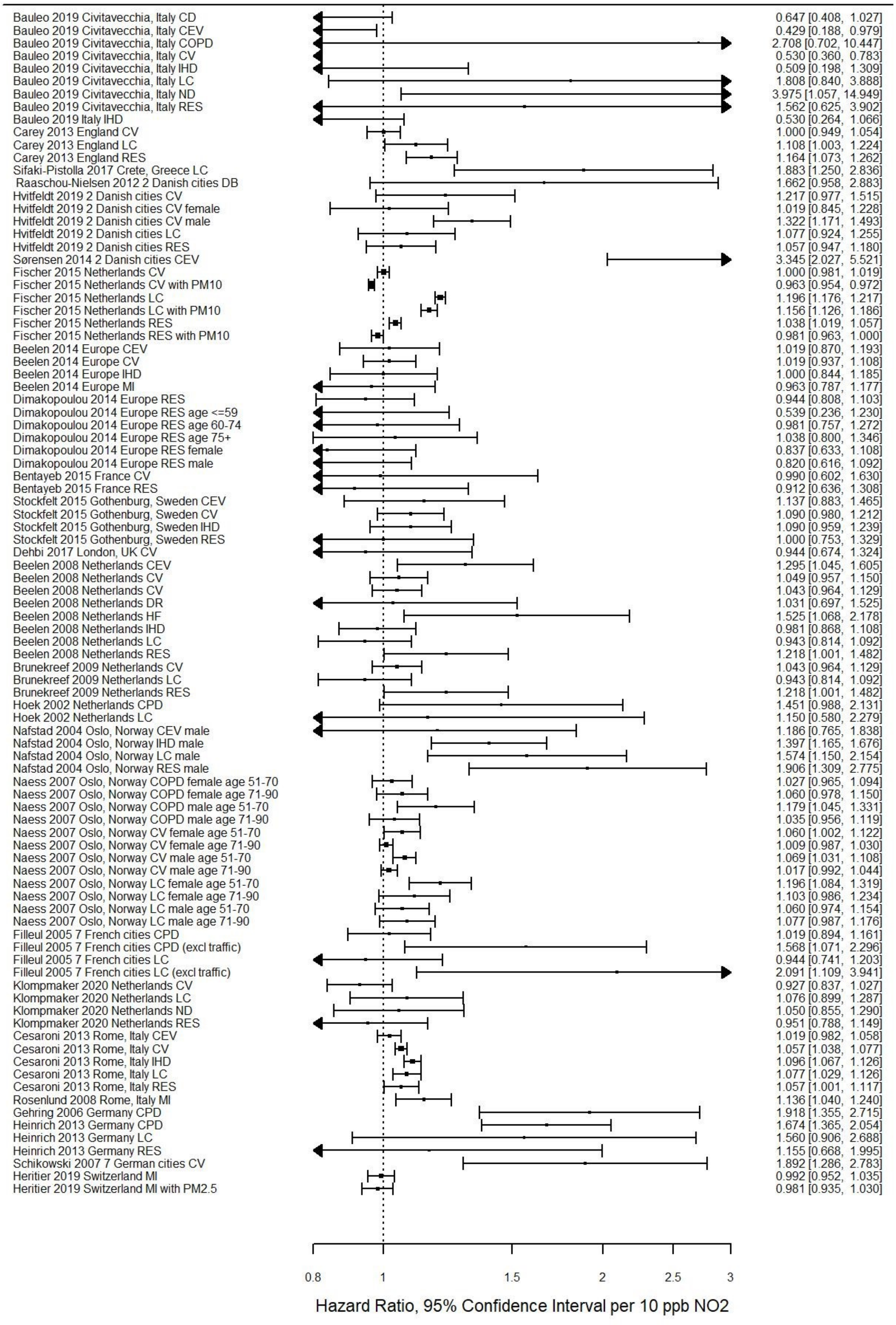
Bauleo 2019 Civitavecchia, Italy CD 0.647 [0.408, 1.027]
Bauleo 2019 Civitavecchia, Italy CEV 0.429 [0.188, 0.979]
Bauleo 2019 Civitavecchia, Italy COPD 2.708 [0.702, 10.447]
Bauleo 2019 Civitavecchia, Italy CV 0.530 [0.360, 0.783]
Bauleo 2019 Civitavecchia, Italy IHD 0.509 [0.198, 1.309]
Bauleo 2019 Civitavecchia, Italy LC 1.808 [0.840, 3.888]
Bauleo 2019 Civitavecchia, Italy ND 3.975 [1.057, 14.949]
Bauleo 2019 Civitavecchia, Italy RES 1.562 [0.625, 3.902]
Bauleo 2019 Italy IHD 0.530 [0.264, 1.066]
Carey 2013 England CV 1.000 [0.949, 1.054]
Carey 2013 England LC 1.108 [1.003, 1.224]
Carey 2013 England RES 1.164 [1.073, 1.262]
Sifaki-Pistolla 2017 Crete, Greece LC 1.883 [1.250, 2.836]
Raaschou-Nielsen 2012 2 Danish cities DB 1.662 [0.958, 2.883]
Hvitfeldt 2019 2 Danish cities CV 1.217 [0.977, 1.515]
Hvitfeldt 2019 2 Danish cities CV female 1.019 [0.845, 1.228]
Hvitfeldt 2019 2 Danish cities CV male 1.322 [1.171, 1.493]
Hvitfeldt 2019 2 Danish cities LC 1.077 [0.924, 1.255]
Hvitfeldt 2019 2 Danish cities RES 1.057 [0.947, 1.180]
Sørensen 2014 2 Danish cities CEV 3.345 [2.027, 5.521]
Fischer 2015 Netherlands CV 1.000 [0.981, 1.019]
Fischer 2015 Netherlands CV with PM10 0.963 [0.954, 0.972]
Fischer 2015 Netherlands LC 1.196 [1.176, 1.217]
Fischer 2015 Netherlands LC with PM10 1.156 [1.126, 1.186]
Fischer 2015 Netherlands RES 1.038 [1.019, 1.057]
Fischer 2015 Netherlands RES with PM10 0.981 [0.963, 1.000]
Beelen 2014 Europe CEV 1.019 [0.870, 1.193]
Beelen 2014 Europe CV 1.019 [0.937, 1.108]
Beelen 2014 Europe IHD 1.000 [0.844, 1.185]
Beelen 2014 Europe MI 0.963 [0.787, 1.177]
Dimakopoulou 2014 Europe RES 0.944 [0.808, 1.103]
Dimakopoulou 2014 Europe RES age <=59 0.539 [0.236, 1.230]
Dimakopoulou 2014 Europe RES age 60-74 0.981 [0.757, 1.272]
Dimakopoulou 2014 Europe RES age 75+ 1.038 [0.800, 1.346]
Dimakopoulou 2014 Europe RES female 0.837 [0.633, 1.108]
Dimakopoulou 2014 Europe RES male 0.820 [0.616, 1.092]
Bentayeb 2015 France CV 0.990 [0.602, 1.630]
Bentayeb 2015 France RES 0.912 [0.636, 1.308]
Stockfelt 2015 Gothenburg, Sweden CEV 1.137 [0.883, 1.465]
Stockfelt 2015 Gothenburg, Sweden CV 1.090 [0.980, 1.212]
Stockfelt 2015 Gothenburg, Sweden IHD 1.090 [0.959, 1.239]
Stockfelt 2015 Gothenburg, Sweden RES 1.000 [0.753, 1.329]
Dehbi 2017 London, UK CV 0.944 [0.674, 1.324]
Beelen 2008 Netherlands CEV 1.295 [1.045, 1.605]
Beelen 2008 Netherlands CV 1.049 [0.957, 1.150]
Beelen 2008 Netherlands CV 1.043 [0.964, 1.129]
Beelen 2008 Netherlands DR 1.031 [0.697, 1.525]
Beelen 2008 Netherlands HF 1.525 [1.068, 2.178]
Beelen 2008 Netherlands IHD 0.981 [0.868, 1.108]
Beelen 2008 Netherlands LC 0.943 [0.814, 1.092]
Beelen 2008 Netherlands RES 1.218 [1.001, 1.482]
Brunekreef 2009 Netherlands CV 1.043 [0.964, 1.129]
Brunekreef 2009 Netherlands LC 0.943 [0.814, 1.092]
Brunekreef 2009 Netherlands RES 1.218 [1.001, 1.482]
Hoek 2002 Netherlands CPD 1.451 [0.988, 2.131]
Hoek 2002 Netherlands LC 1.150 [0.580, 2.279]
Nafstad 2004 Oslo, Norway CEV male 1.186 [0.765, 1.838]
Nafstad 2004 Oslo, Norway IHD male 1.397 [1.165, 1.676]
Nafstad 2004 Oslo, Norway LC male 1.574 [1.150, 2.154]
Nafstad 2004 Oslo, Norway RES male 1.906 [1.309, 2.775]
Naess 2007 Oslo, Norway COPD female age 51-70 1.027 [0.965, 1.094]
Naess 2007 Oslo, Norway COPD female age 71-90 1.060 [0.978, 1.150]
Naess 2007 Oslo, Norway COPD male age 51-70 1.179 [1.045, 1.331]
Naess 2007 Oslo, Norway COPD male age 71-90 1.035 [0.956, 1.119]
Naess 2007 Oslo, Norway CV female age 51-70 1.060 [1.002, 1.122]
Naess 2007 Oslo, Norway CV female age 71-90 1.009 [0.987, 1.030]
Naess 2007 Oslo, Norway CV male age 51-70 1.069 [1.031, 1.108]
Naess 2007 Oslo, Norway CV male age 71-90 1.017 [0.992, 1.044]
Naess 2007 Oslo, Norway LC female age 51-70 1.196 [1.084, 1.319]
Naess 2007 Oslo, Norway LC female age 71-90 1.103 [0.986, 1.234]
Naess 2007 Oslo, Norway LC male age 51-70 1.060 [0.974, 1.154]
Naess 2007 Oslo, Norway LC male age 71-90 1.077 [0.987, 1.176]
Filleul 2005 7 French cities CPD 1.019 [0.894, 1.161]
Filleul 2005 7 French cities CPD (excl traffic) 1.568 [1.071, 2.296]
Filleul 2005 7 French cities LC 0.944 [0.741, 1.203]
Filleul 2005 7 French cities LC (excl traffic) 2.091 [1.109, 3.941]
Klompmaker 2020 Netherlands CV 0.927 [0.837, 1.027]
Klompmaker 2020 Netherlands LC 1.076 [0.899, 1.287]
Klompmaker 2020 Netherlands ND 1.050 [0.855, 1.290]
Klompmaker 2020 Netherlands RES 0.951 [0.788, 1.149]
Cesaroni 2013 Rome, Italy CEV 1.019 [0.982, 1.058]
Cesaroni 2013 Rome, Italy CV 1.057 [1.038, 1.077]
Cesaroni 2013 Rome, Italy IHD 1.096 [1.067, 1.126]
Cesaroni 2013 Rome, Italy LC 1.077 [1.029, 1.126]
Cesaroni 2013 Rome, Italy RES 1.057 [1.001, 1.117]
Rosenlund 2008 Rome, Italy MI 1.136 [1.040, 1.240]
Gehring 2006 Germany CPD 1.918 [1.355, 2.715]
Heinrich 2013 Germany CPD 1.674 [1.365, 2.054]
Heinrich 2013 Germany LC 1.560 [0.906, 2.688]
Heinrich 2013 Germany RES 1.155 [0.668, 1.995]
Schikowski 2007 7 German cities CV 1.892 [1.286, 2.783]
Heritier 2019 Switzerland MI 0.992 [0.952, 1.035]
Heritier 2019 Switzerland MI with PM2.5 0.981 [0.935, 1.030]
0.8
1
1.5
2
2.5
3
Hazard Ratio, 95% Confidence Interval per 10 ppb NO2

F) Other

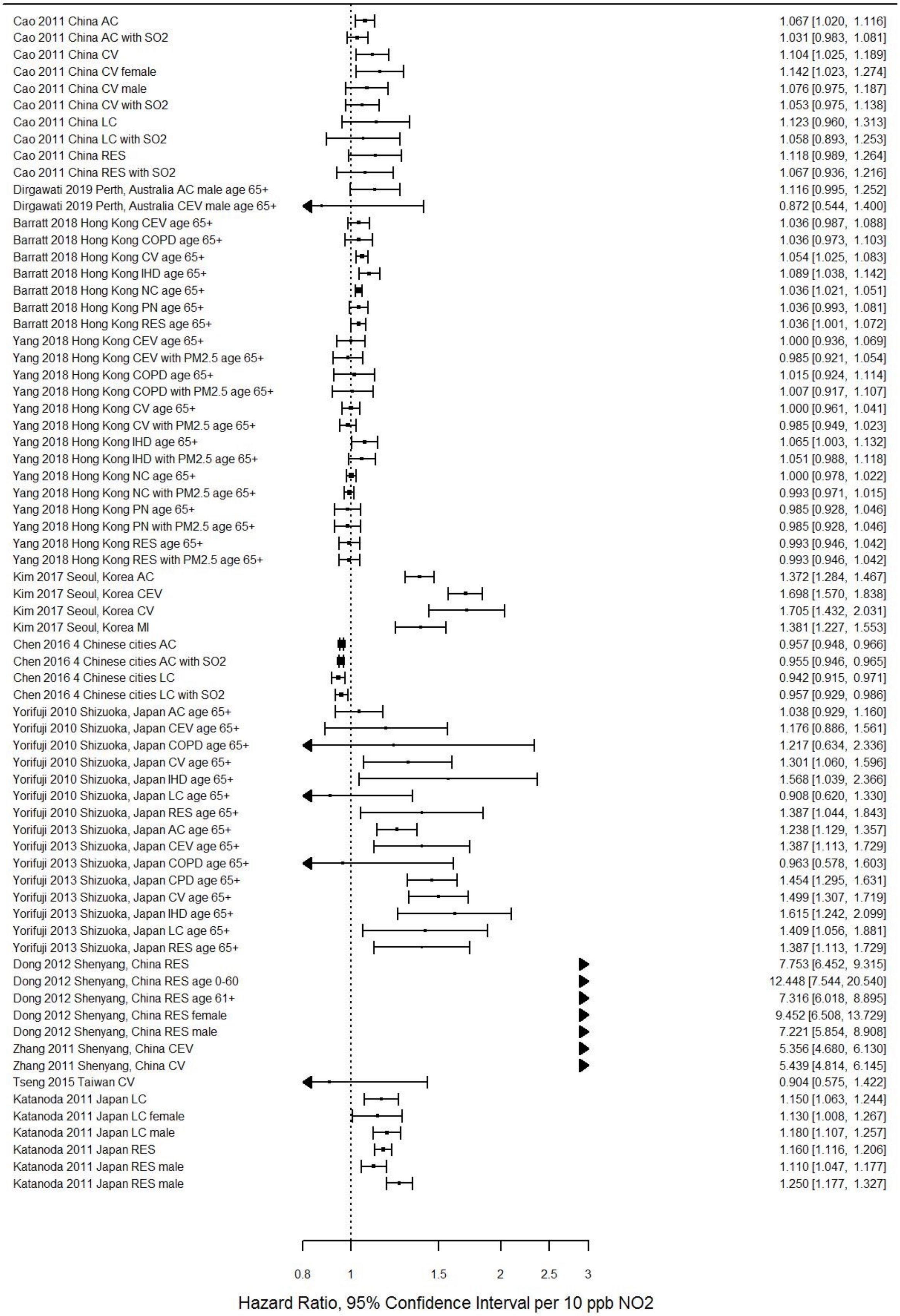

Cao 2011 China AC 1.067 [1.020, 1.116]
Cao 2011 China AC with SO2 1.031 [0.983, 1.081]
Cao 2011 China CV 1.104 [1.025, 1.189]
Cao 2011 China CV female 1.142 [1.023, 1.274]
Cao 2011 China CV male 1.076 [0.975, 1.187]
Cao 2011 China CV with SO2 1.053 [0.975, 1.138]
Cao 2011 China LC 1.123 [0.960, 1.313]
Cao 2011 China LC with SO2 1.058 [0.893, 1.253]
Cao 2011 China RES 1.118 [0.989, 1.264]
Cao 2011 China RES with SO2 1.067 [0.936, 1.216]
Dirgawati 2019 Perth, Australia AC male age 65+ 1.116 [0.995, 1.252]
Dirgawati 2019 Perth, Australia CEV male age 65+ 0.872 [0.544, 1.400]
Barratt 2018 Hong Kong CEV age 65+ 1.036 [0.987, 1.088]
Barratt 2018 Hong Kong COPD age 65+ 1.036 [0.973, 1.103]
Barratt 2018 Hong Kong CV age 65+ 1.054 [1.025, 1.083]
Barratt 2018 Hong Kong IHD age 65+ 1.089 [1.038, 1.142]
Barratt 2018 Hong Kong NC age 65+ 1.036 [1.021, 1.051]
Barratt 2018 Hong Kong PN age 65+ 1.036 [0.993, 1.081]
Barratt 2018 Hong Kong RES age 65+ 1.036 [1.001, 1.072]
Yang 2018 Hong Kong CEV age 65+ 1.000 [0.936, 1.069]
Yang 2018 Hong Kong CEV with PM2.5 age 65+ 0.985 [0.921, 1.054]
Yang 2018 Hong Kong COPD age 65+ 1.015 [0.924, 1.114]
Yang 2018 Hong Kong COPD with PM2.5 age 65+ 1.007 [0.917, 1.107]
Yang 2018 Hong Kong CV age 65+ 1.000 [0.961, 1.041]
Yang 2018 Hong Kong CV with PM2.5 age 65+ 0.985 [0.949, 1.023]
Yang 2018 Hong Kong IHD age 65+ 1.065 [1.003, 1.132]
Yang 2018 Hong Kong IHD with PM2.5 age 65+ 1.051 [0.988, 1.118]
Yang 2018 Hong Kong NC age 65+ 1.000 [0.978, 1.022]
Yang 2018 Hong Kong NC with PM2.5 age 65+ 0.993 [0.971, 1.015]
Yang 2018 Hong Kong PN age 65+ 0.985 [0.928, 1.046]
Yang 2018 Hong Kong PN with PM2.5 age 65+ 0.985 [0.928, 1.046]
Yang 2018 Hong Kong RES age 65+ 0.993 [0.946, 1.042]
Yang 2018 Hong Kong RES with PM2.5 age 65+ 0.993 [0.946, 1.042]
Kim 2017 Seoul, Korea AC 1.372 [1.284, 1.467]
Kim 2017 Seoul, Korea CEV 1.698 [1.570, 1.838]
Kim 2017 Seoul, Korea CV 1.705 [1.432, 2.031]
Kim 2017 Seoul, Korea MI 1.381 [1.227, 1.553]
Chen 2016 4 Chinese cities AC 0.957 [0.948, 0.966]
Chen 2016 4 Chinese cities AC with SO2 0.955 [0.946, 0.965]
Chen 2016 4 Chinese cities LC 0.942 [0.915, 0.971]
Chen 2016 4 Chinese cities LC with SO2 0.957 [0.929, 0.986]
Yorifuji 2010 Shizuoka, Japan AC age 65+ 1.038 [0.929, 1.160]
Yorifuji 2010 Shizuoka, Japan CEV age 65+ 1.176 [0.886, 1.561]
Yorifuji 2010 Shizuoka, Japan COPD age 65+ 1.217 [0.634, 2.336]
Yorifuji 2010 Shizuoka, Japan CV age 65+ 1.301 [1.060, 1.596]
Yorifuji 2010 Shizuoka, Japan IHD age 65+ 1.568 [1.039, 2.366]
Yorifuji 2010 Shizuoka, Japan LC age 65+ 0.908 [0.620, 1.330]
Yorifuji 2010 Shizuoka, Japan RES age 65+ 1.387 [1.044, 1.843]
Yorifuji 2013 Shizuoka, Japan AC age 65+ 1.238 [1.129, 1.357]
Yorifuji 2013 Shizuoka, Japan CEV age 65+ 1.387 [1.113, 1.729]
Yorifuji 2013 Shizuoka, Japan COPD age 65+ 0.963 [0.578, 1.603]
Yorifuji 2013 Shizuoka, Japan CPD age 65+ 1.454 [1.295, 1.631]
Yorifuji 2013 Shizuoka, Japan CV age 65+ 1.499 [1.307, 1.719]
Yorifuji 2013 Shizuoka, Japan IHD age 65+ 1.615 [1.242, 2.099]
Yorifuji 2013 Shizuoka, Japan LC age 65+ 1.409 [1.056, 1.881]
Yorifuji 2013 Shizuoka, Japan RES age 65+ 1.387 [1.113, 1.729]
Dong 2012 Shenyang, China RES 7.753 [6.452, 9.315]
Dong 2012 Shenyang, China RES age 0-60 12.448 [7.544, 20.540]
Dong 2012 Shenyang, China RES age 61+ 7.316 [6.018, 8.895]
Dong 2012 Shenyang, China RES female 9.452 [6.508, 13.729]
Dong 2012 Shenyang, China RES male 7.221 [5.854, 8.908]
Zhang 2011 Shenyang, China CEV 5.356 [4.680, 6.130]
Zhang 2011 Shenyang, China CV 5.439 [4.814, 6.145]
Tseng 2015 Taiwan CV 0.904 [0.575, 1.422]
Katanoda 2011 Japan LC 1.150 [1.063, 1.244]
Katanoda 2011 Japan LC female 1.130 [1.008, 1.267]
Katanoda 2011 Japan LC male 1.180 [1.107, 1.257]
Katanoda 2011 Japan RES 1.160 [1.116, 1.206]
Katanoda 2011 Japan RES male 1.110 [1.047, 1.177]
Katanoda 2011 Japan RES male 1.250 [1.177, 1.327]
0.8
1
1.5
2
2.5
3
Hazard Ratio, 95% Confidence Interval per 10 ppb NO2

AC/NC, All cause/Natural cause; BC, brain cancer; C, cardiac; CA, cardiac arrest; CC, colorectal cancer; CPD, cardiopulmonary disease; CV, cardiovascular; CEV, cerebrovascular; COPD, chronic obstructive pulmonary disease; DB, diabetes; IHD, ischemic heart disease; LC, lung cancer; MI, myocardial infarction; ND, neurologic disease; PN, pneumonia; RES, respiratory.
