# Supplementary material for: Systematic review and meta-analysis of cohort studies of long term outdoor nitrogen dioxide exposure and mortality
Source: PLoS One. 2021 Feb 4;16(2):e0246451. doi: 10.1371/journal.pone.0246451 (PMC7861378; doi:10.1371/journal.pone.0246451)

Online supplementary figure S4. Hazard ratios from single pollutant models from individual cohort studies and pooled estimates by region

A) Cardiovascular mortality

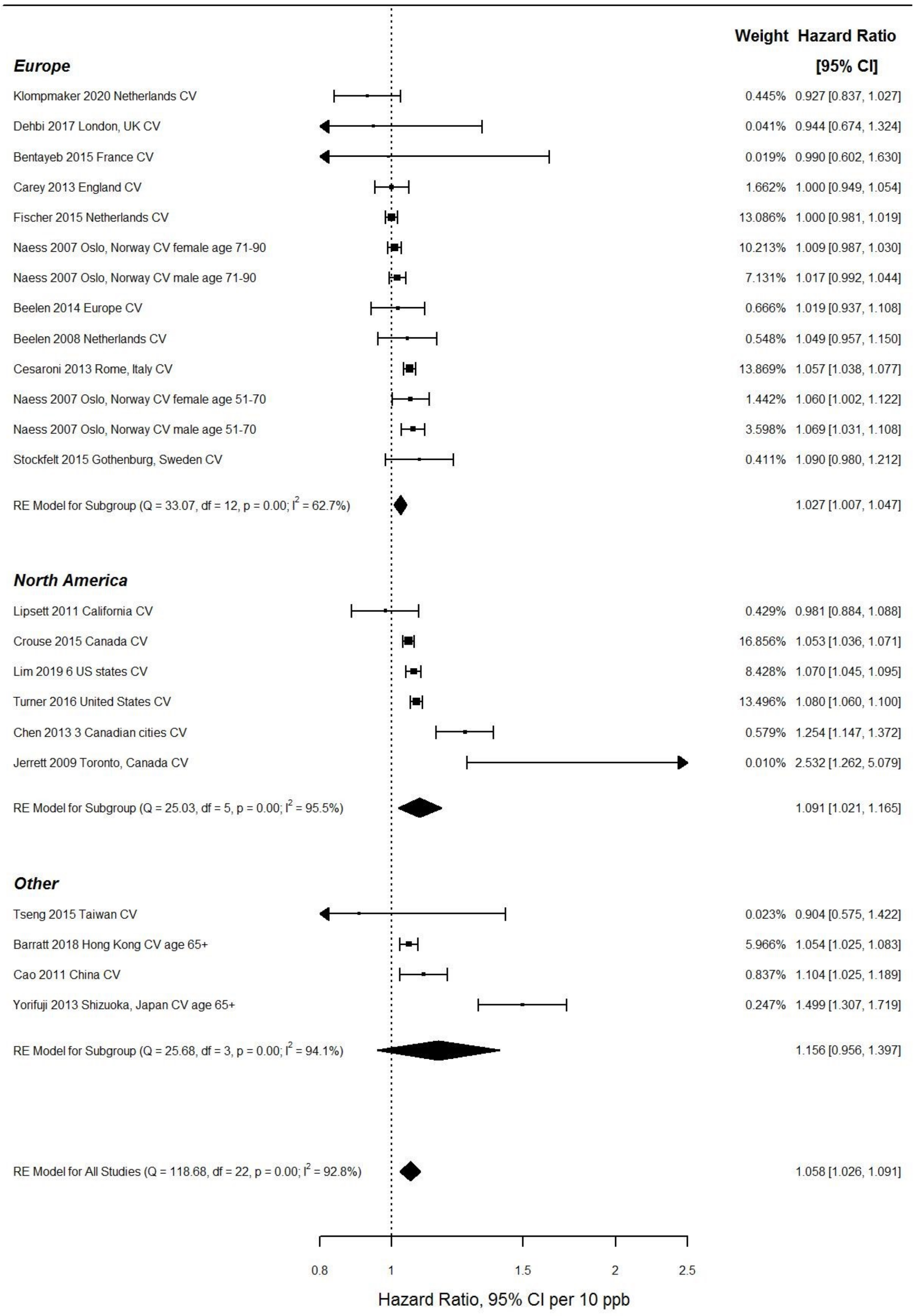

B) Lung cancer mortality

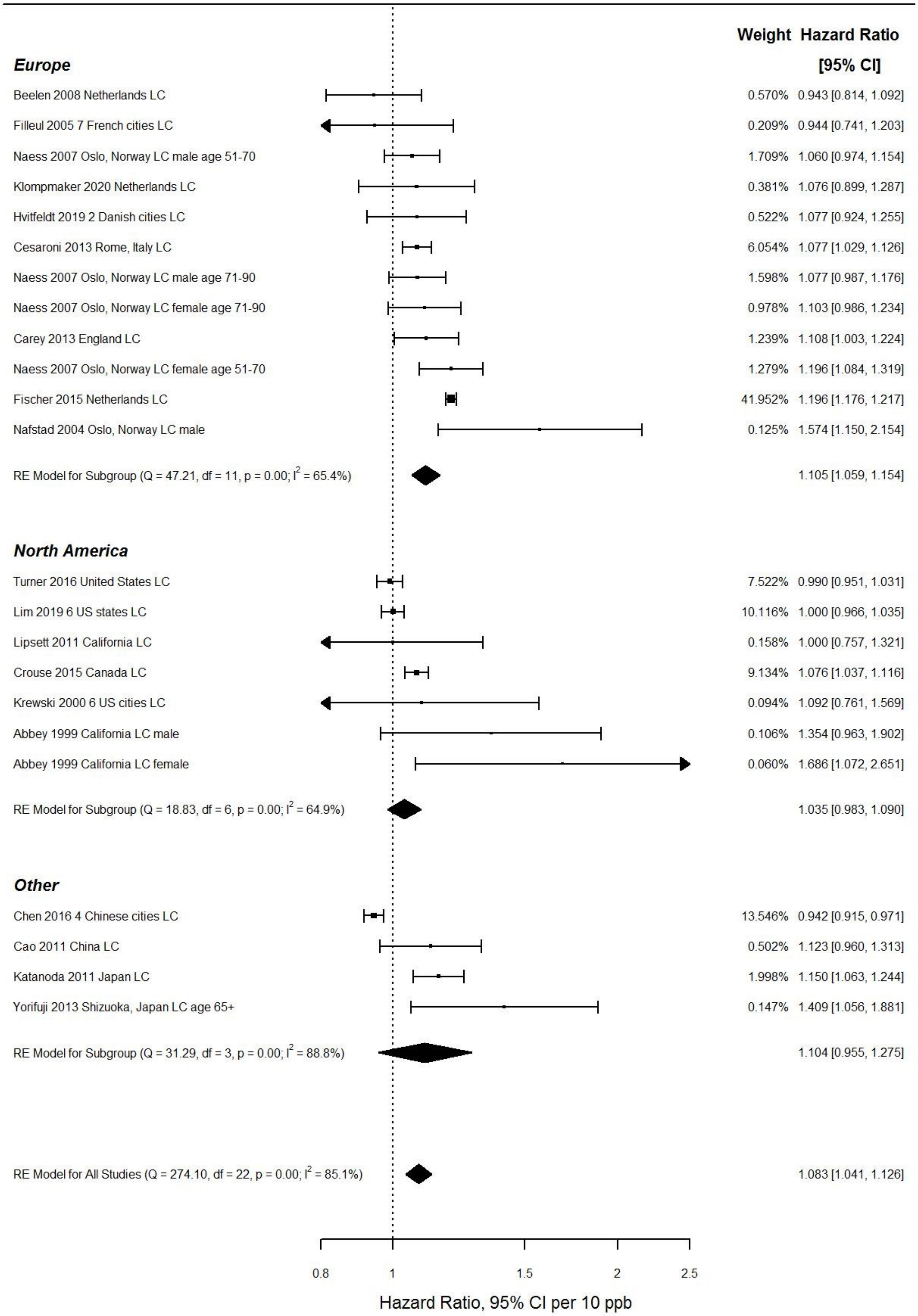

C) Respiratory mortality

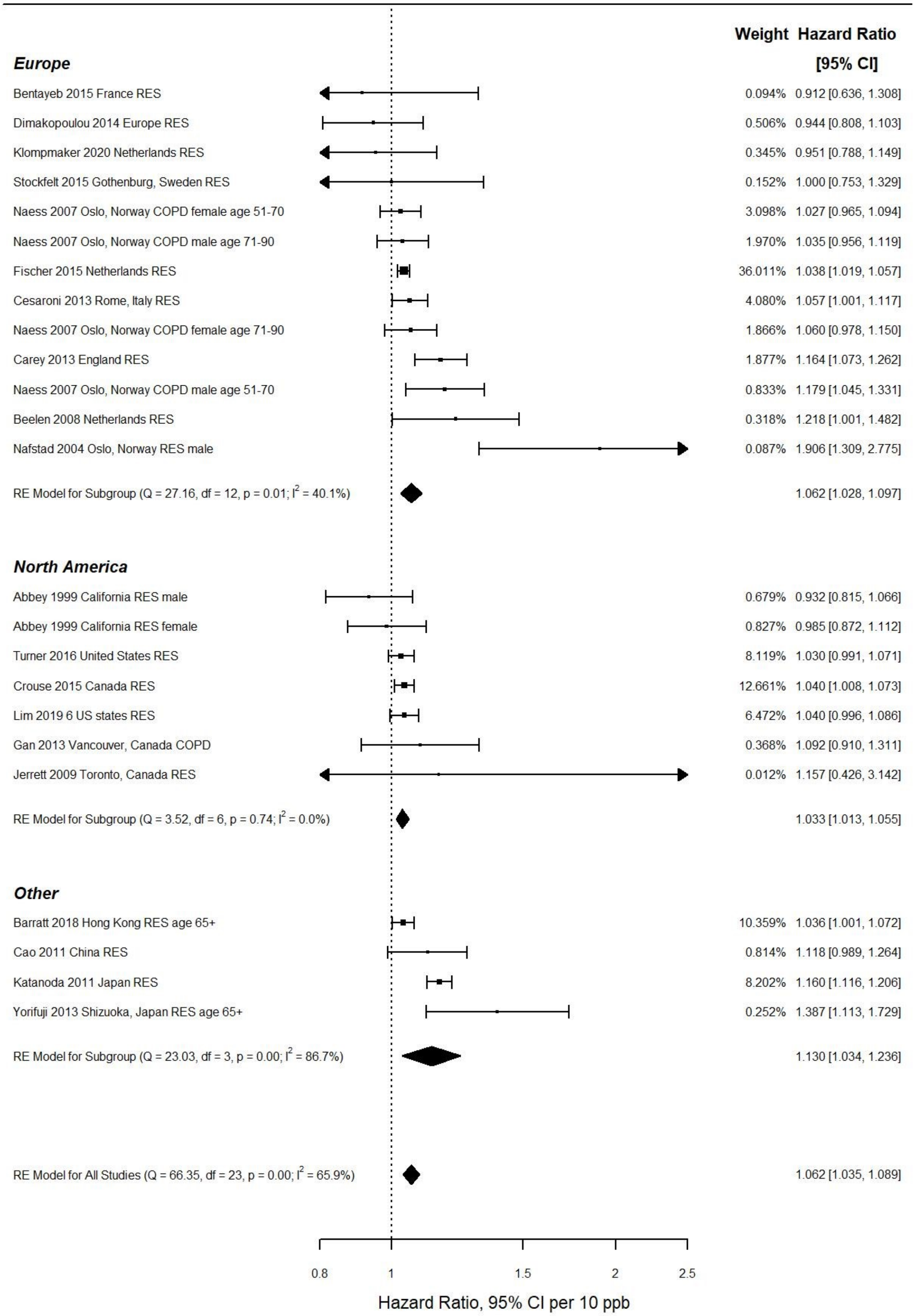

D) Cerebrovascular mortality

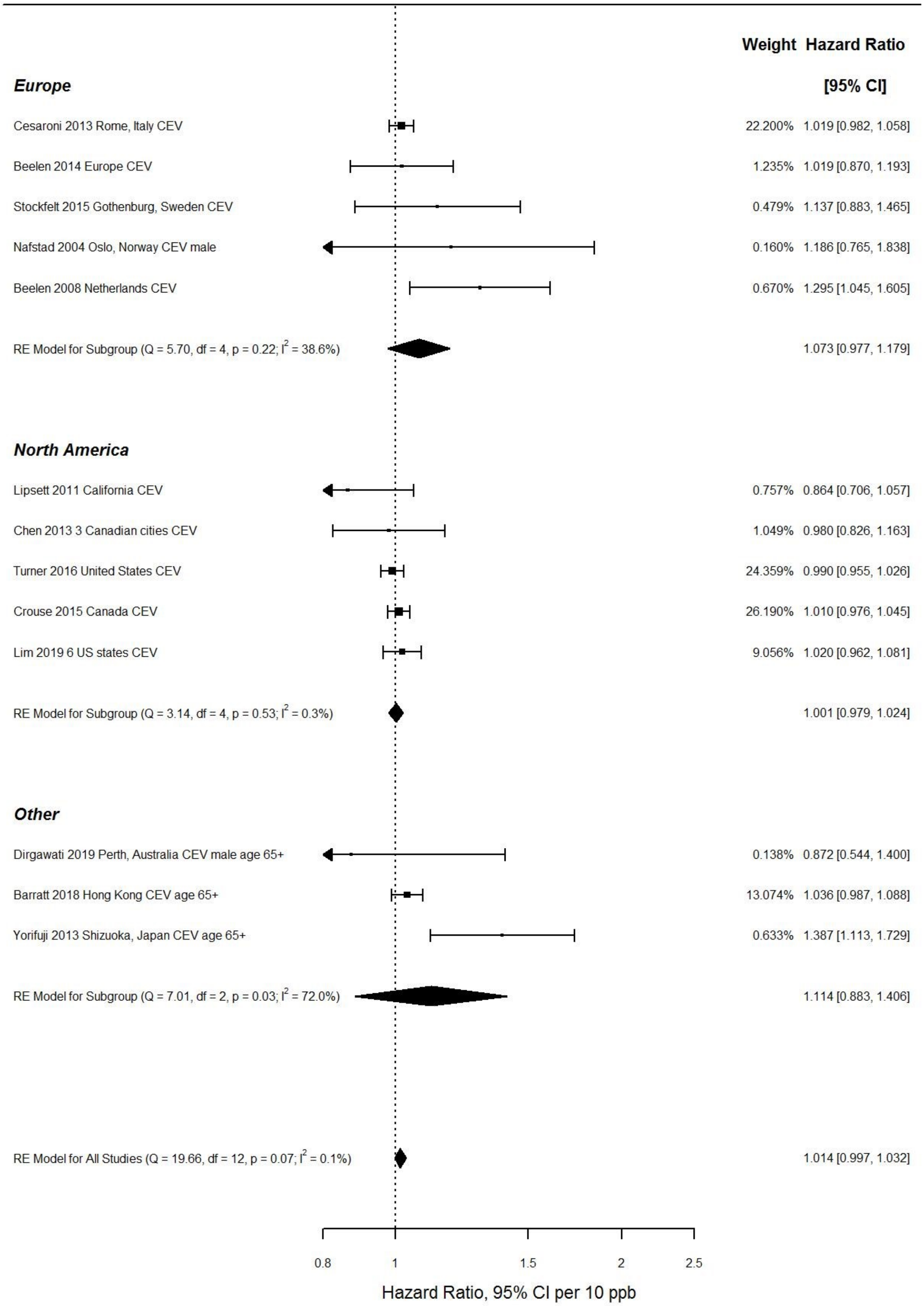

E) Ischemic heart disease mortality

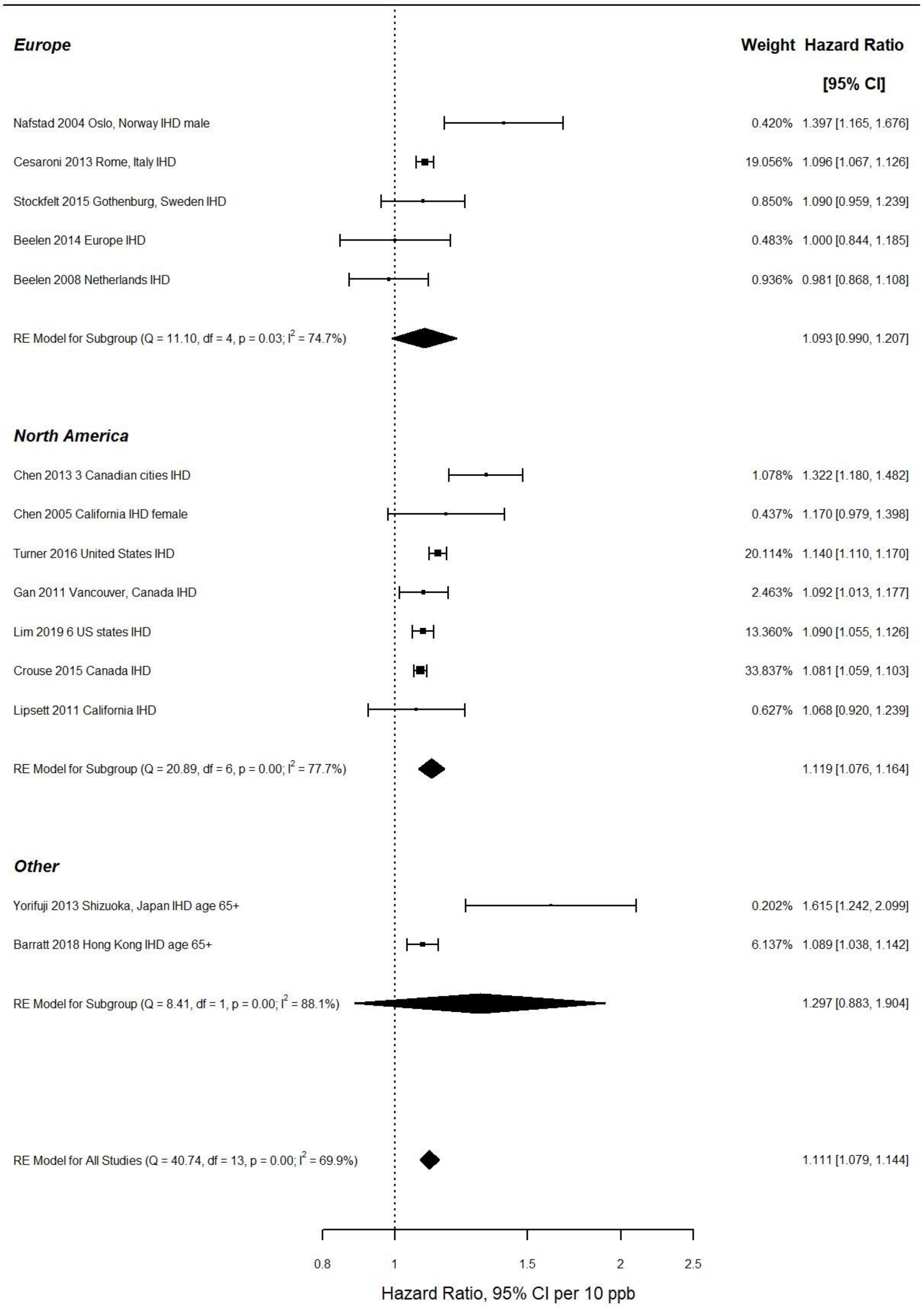

Supplement: S4 Fig — (PDF) [file pone.0246451.s004.pdf]
