# Supplementary material for: Systematic review and meta-analysis of cohort studies of long term outdoor nitrogen dioxide exposure and mortality
Source: PLoS One. 2021 Feb 4;16(2):e0246451. doi: 10.1371/journal.pone.0246451 (PMC7861378; doi:10.1371/journal.pone.0246451)
Supplement: S5 Fig — (PDF) [file pone.0246451.s005.pdf]

Online supplementary figure S5. Funnel plot of log(Hazard Ratio) vs. standard error, lung cancer mortality

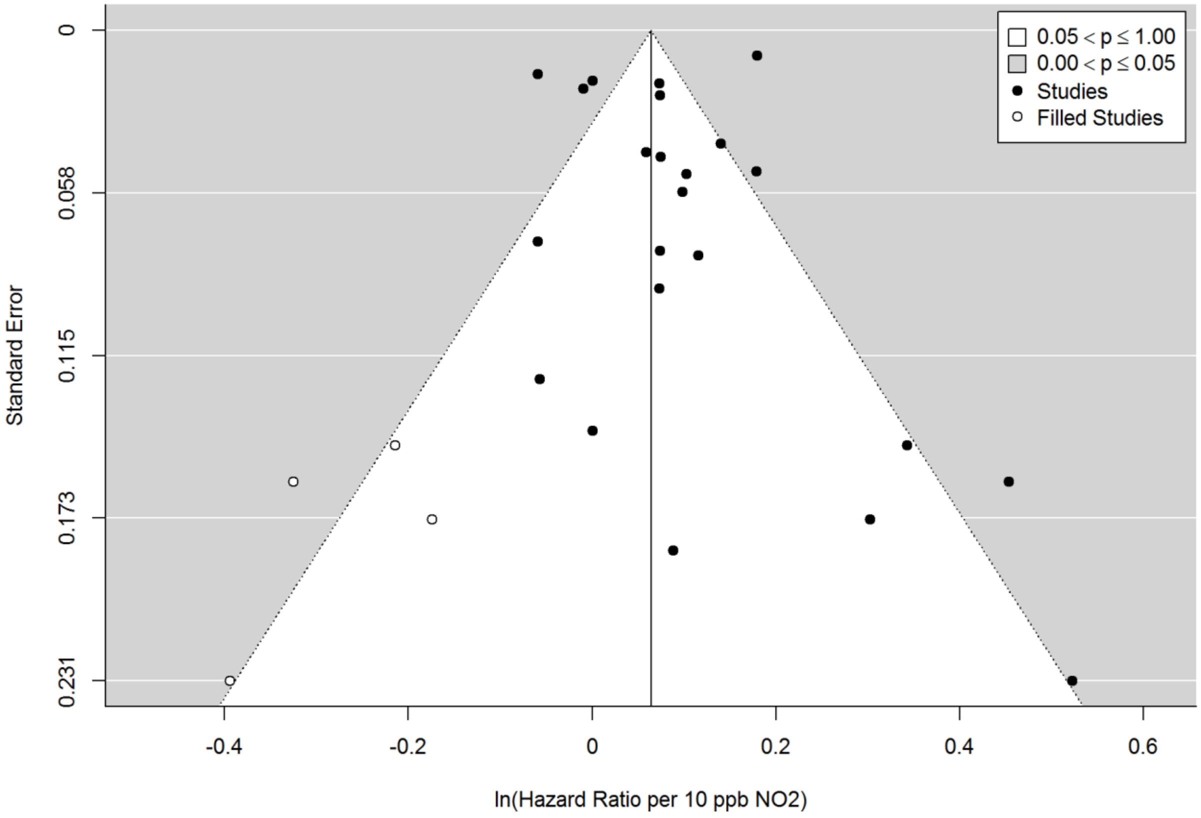

Filled circles represent observed values, open circles represent missing studies identified with trim and fill, and the vertical line represents the log of the pooled hazard ratio. In the absence of publication bias, points should be symmetrically distributed around the vertical line, with smaller studies (larger standard errors on vertical axis) more widely scattered. Filling the plot with points mirroring observed values corrects for apparently missing smaller and/or negative studies which may have been suppressed due to publication bias.
