# Supplementary material for: Systematic review and meta-analysis of cohort studies of long term outdoor nitrogen dioxide exposure and mortality
Source: PLoS One. 2021 Feb 4;16(2):e0246451. doi: 10.1371/journal.pone.0246451 (PMC7861378; doi:10.1371/journal.pone.0246451)
Supplement: S1 File — (PDF) [file pone.0246451.s006.pdf]

Systematic review and meta-analysis of effects of outdoor nitrogen dioxide: short term exposure and cardio-respiratory morbidity and long term exposure and mortality

David Stieb, Ninon Lyrette, Eric Lavigne, Robyn Hocking, Carine Zheng, Dina Salama, Hwashin Shin

Citation

David Stieb, Ninon Lyrette, Eric Lavigne, Robyn Hocking, Carine Zheng, Dina Salama, Hwashin Shin. Systematic review and meta-analysis of effects of outdoor nitrogen dioxide: short term exposure and cardio-respiratory morbidity and long term exposure and mortality. PROSPERO 2018 CRD42018084497 Available from: https://www.crd.york.ac.uk/prospero/display_record.php?ID=CRD42018084497

Review question

Is short term exposure to outdoor nitrogen dioxide (NO2) associated with morbidity from chronic obstructive pulmonary disease (COPD), respiratory infections, and cardiovascular disease? Is long term exposure to outdoor NO2 associated with all-cause or cause-specific mortality?

Searches

MEDLINE, Embase, CENTRAL, Global Health and Toxline databases will be searched using terms developed by a librarian (R. Hocking). Publications in abstract form only will be excluded. Manual searches will be completed of reference lists of all relevant studies. There are no restrictions on language or publication date. The search strategy will undergo PRESS peer review.

Types of study to be included

Time series, case-crossover studies (short term exposure), cohort studies (long-term exposure).

Condition or domain being studied

Hospital admissions, emergency visits, physician office visits for COPD, respiratory infections, cardiovascular disease. All-cause and cause-specific mortality.

Participants/population

Humans.

Intervention(s), exposure(s)

Exposure to outdoor nitrogen dioxide (and other oxides of nitrogen).

Comparator(s)/control

Lower levels of exposure.

Main outcome(s)

Counts of hospital admissions, emergency visits, physician office visits for COPD, respiratory infections, cardiovascular disease. Counts of all-cause and cause-specific mortality.

* Measures of effect

Morbidity effects reported as regression coefficients, odds ratios or relative risks associated with exposures over days to weeks, expressed per specified increment in exposure. Mortality effects reported as hazard ratios associated with exposures over years expressed per increment in exposure.

Additional outcome(s)
None

Data extraction (selection and coding)
Two individuals will independently screen all studies retrieved from literature searches based on title and abstract. Where relevance cannot be determined based on abstract and title, the full text will be reviewed. Discrepancies between reviewers will be resolved by consensus and/or consultation of a third reviewer if necessary. A pilot test of screening procedures will be conducted prior to their implementation. Two individuals will independently extract study characteristics and results from each study and enter these into Distiller SR. Discrepancies will be resolved by consensus and/or consultation of a third reviewer if necessary. Where required data are not provided in a paper, authors will be contacted by e-mail, and if non-responsive within one month will be reminded. A pilot test of data extraction procedures will be conducted prior to their implementation. Data to be extracted include: bibliographic data, study location and timing, design, population age group(s), sample size, outcome (including ICD code(s) if available), method of exposure assessment, pollutant (including name, averaging time, units, lag, descriptive statistics), type of regression model, effect measure and standard error or confidence interval, model covariates and their specification.

Risk of bias (quality) assessment
Modifications of the Cochrane collaboration “Risk of Bias” tool and the Agency for Healthcare Research and Quality’s (AHRQ) criteria for the Navigation Guide systematic review methodology will be employed. Additional criteria related to sensitivity (ability to detect a true effect) will also be applied. Each study will also be characterized according to funding source, declared conflicts of interest, and occurrence of retractions or corrections. The above criteria will be applied independently to each study by two reviewers. Discrepancies will be resolved by consensus and/or consultation of a third or additional reviewers. A pilot test of risk of bias assessment procedures will be conducted prior to their implementation.

Strategy for data synthesis
Appropriateness of pooling results across studies will be determined based on sufficient similarity with respect to study design, population, exposure, outcome and type of data or summary statistics available. Two senior investigators will independently assess whether sufficient comparability exists among studies to warrant pooling results.

Analysis of subgroups or subsets
Where possible (4 or more studies per subgroup), subgroup analyses will be conducted by key characteristics such as region, age group, method of exposure characterization, and risk of bias characterization.

Contact details for further information
David Stieb
dave.stieb@canada.ca

Organisational affiliation of the review
Health Canada/University of Ottawa

Review team members and their organisational affiliations
Dr David Stieb. Health Canada/ University of Ottawa
Ms Ninon Lyrette. Health Canada
Dr Eric Lavigne. Health Canada/ University of Ottawa
Ms Robyn Hocking. Health Canada
Ms Carine Zheng. University of Ottawa
Ms Dina Salama. University of Ottawa
Dr Hwashin Shin. Health Canada/ Queen's University

Type and method of review

Epidemiologic, Meta-analysis, Systematic review

Anticipated or actual start date
02 October 2017

Anticipated completion date
31 March 2019

Funding sources/sponsors
Health Canada

Conflicts of interest

Language
(there is not an English language summary)

Country
Canada

Stage of review
Review Ongoing

Subject index terms status
Subject indexing assigned by CRD

Subject index terms
Air Pollution; Humans; Nitrogen Dioxide; Particulate Matter

Date of registration in PROSPERO
16 January 2018

Date of first submission
20 December 2017

Stage of review at time of this submission

Stage	Started	Completed
Preliminary searches	Yes	No
Piloting of the study selection process	Yes	No
Formal screening of search results against eligibility criteria	No	No
Data extraction	No	No
Risk of bias (quality) assessment	No	No
Data analysis	No	No

The record owner confirms that the information they have supplied for this submission is accurate and complete and they understand that deliberate provision of inaccurate information or omission of data may be construed as scientific misconduct.

The record owner confirms that they will update the status of the review when it is completed and will add publication details in due course.

Versions

16 January 2018
19 February 2018

PROSPERO
This information has been provided by the named contact for this review. CRD has accepted this information in good faith and registered the review in PROSPERO. The registrant confirms that the information supplied for this submission is accurate and complete. CRD bears no responsibility or liability for the content of this registration record, any associated files or external websites.
