# Supplementary material for: Systematic review and meta-analysis of cohort studies of long term outdoor nitrogen dioxide exposure and mortality
Source: PLoS One. 2021 Feb 4;16(2):e0246451. doi: 10.1371/journal.pone.0246451 (PMC7861378; doi:10.1371/journal.pone.0246451)
Supplement: S1 Table — (PDF) [file pone.0246451.s009.pdf]

Online supplementary table S1 – Details of Search Strategies

Database(s): Ovid MEDLINE(R) Epub Ahead of Print, In-Process & Other Non-Indexed Citations, Ovid MEDLINE(R) Daily, Ovid MEDLINE and Versions(R) 1946 to February 07, 2018. Updated February 25, 2020.		
**#**	**Searches**	**Results**
1	exp nitrogen oxides/	103678
2	((nitrogen adj2 oxide*) or nitric oxide* or dinitrogen monoxide or dinitrogen oxide or hyponitrous acid anhydride or nitrogen hypoxide or nitrogen monoxide or nitrogen protoxide).tw,kf.	137450
3	(nitrogen dioxide or nitrogen peroxide or "NO_2" or "NOx").tw,kf.	20441
4	or/1-3 [NOx]	183579
5	((respira* or lung* or bronch* or cilia* or laryng* or pulmonar* or alveol* or aortic* or vascular* or cerebrovasc* or pericard* or endocard* or pleural or thorax or pneumo* or thoracic or trachea* or cardio* or cardia* or heart? or vascula* or atria* or ventric* or myocard* or coronary) adj2 (health or symptom* or infect* or disease* or disorder* or dysfunction* or syndrome* granuloma* or neoplas* or cancer* or tumor* or tumour* or ischemia* or mortal* or morbid* or fatal* or death? or admission* or hospital* or emergency or emergencies)).tw,kf.	1061933
6	exp Respiratory Tract Infections/	332704
7	exp Respiratory Tract Diseases/	1227094
8	(pleural empyema or whooping cough or pertussis or bronchiectasis).tw,kf.	37905
9	((bronchial adj2 (fistula* or hyperreactiv* or neoplasm* or spasm*)) or bronchitis or bronchiolitis or bronchogenic cyst* or bronchopneumonia* or bronchiectas?s or bronchospasm*).tw,kf.	52145
10	((laryng* adj2 (edema* or neoplasm* or nerve injur*)) or laryngitis or croup or supraglottitis or tuberculos* or laryngotracheobronchit*).tw,kf.	203143
11	acute chest syndrome*.tw,kf.	890
12	cystic fibrosis.tw,kf.	39849
13	lung abscess*.tw,kf.	3038
14	blastomycos*.tw,kf.	2306
15	pneumonia*.tw,kf.	146884
16	pulmonary aspergillos*.tw,kf.	2989
17	alveolitis.tw,kf.	4032
18	pneumonitis.tw,kf.	12372
19	((chronic obstructive adj2 (pulmonary or airway or lung)) or chronic airflow obstruction or "COPD" or "COAD" or "AECOPD" or ("OPD" or obstructive pulmonary)).tw,kf.	58955
20	pulmonary edema*.tw,kf.	13996

21	pulmonary embolism*.tw,kf.	30365
22	respiratory distress syndrome*.tw,kf.	22702
23	bronchiogenic carcinoma*.tw,kf.	110
24	pulmonary nodule*.tw,kf.	5941
25	pancoast syndrome.tw,kf.	134
26	pulmonary sclerosing hemangioma*.tw,kf.	117
27	exp Cardiovascular Diseases/	2166950
28	(NSTEMI or STEMI or cardiogenic shock* or ((cardiovascular or heart or myocardial) adj2 (stroke* or attack* or infarct* or event?)) or ((ischemic* or hemmorhagic or haemmorhagic) adj2 stroke?)).tw,kf.	252316
29	(angina or stenocardi*).tw,kf.	51051
30	(((heart or cardiac or myocardia*) adj2 (failure or decompensat*)) or cardiac edema or paroxysmal dyspnea or cardio-renal syndrome* or cardiorenal syndrome*).tw,kf.	158514
31	(arrhythmia* or dysrhythmia* or bradycard* or brugada syndrome* or commotio cordis or heart block* or long QT syndrome or parasystole or pre-excitation syndrome* or tachycardia* or ((atrial or ventricular) adj2 (fibrillation* or flutter)) or conduction disturbance*).tw,kf.	203105
32	or/5-31 [Health Outcomes]	3883975
33	air/	17354
34	air pollution/ or environmental exposure/ or inhalation exposure/ or exp occupational exposure/ or smog/	146365
35	environmental pollution/	15338
36	exp air pollutants/	77603
37	(air? adj3 (pollut* or environment* or outdoor* or outside*)).tw,kf.	30108
38	Vehicle Emissions/	8668
39	Automobile Driving/	16898
40	Parking Facilities/	351
41	exp transportation/	63060
42	((distanc* or proximit* or close* or near) adj3 (traffic* or road* or highway* or transitway*)).tw,kf.	2099
43	traffic exposure*.tw,kf.	234
44	Power Plants/	5806
45	(power adj2 plant?).tw,kf.	5618
46	((motor* or vehic* or diesel* or gasoline* or fossil fuel* or crude oil* or coal? or natural gas or petrol*) adj4 (exhaust? or emission* or pollut* or vapor* or vapour* or volatile* or effluvia* or	11575

	smoke* or fume? or combust* or haze? or smog* or nitrogen oxide* or NOx or nitrogen dioxide* or NO_2)).tw,kf.	
47	(automobile* or automotive* or autocar* or autobus* or taxi* or engine? or car or cars or truck* or bus or buses or bussing or highway* or high way* or motorway* or motor way or transitway* or road* or parkade* or parking* or carpark* or car park* or traffic or street* or motorcycl* or motorbi* or expressway* or parkway*).tw,kf.	134145
48	or/33-47 [NOx Sources]	418362
49	exp hospitalization/	202889
50	child hospitalized/ or adolescent hospitalized/	6530
51	exp patient care/	844917
52	exp primary health care/	134468
53	exp hospital departments/	166722
54	exp hospitals/	248647
55	health facilities/	13600
56	ambulatory care facilities/	16679
57	Physicians' Offices/	1685
58	(((clinic* or emergen* or doctor* or physician* or pediatric* or paediatric* or cardi* or respir* or healthcare or patient* or medical or primary care or health team or ambulatory care or "walk in") adj4 (admission* or admit* or readmission* or readmit* or discharg* or handoff or hand* off or transfer* or visit? or consult*)) or hospital*).tw,kf.	1233251
59	(("walk in" or clinic* or health care or healthcare or practitioner* or physician* or doctor* or medical or health care) adj3 (appointment* or booking* or office? or meeting*)).tw,kf.	19373
60	((CVD or STEMI or NSTEMI or "MI" or infarct* or cardio* or cardia* or myocard* or pericard* or coronary or respirat* or pulmonary or heart? or vascula* or atria* or ventric*) adj3 (event or events or inciden* or risk? or crisis or crises or symptom* or emergencies or emergency)).tw,kf.	298005
61	(primary adj2 care).tw,kf.	118122
62	exp morbidity/	475798
63	exp mortality/	337377
64	exp epidemiology/	24531
65	epidemiological monitoring/	5805
66	(IQR or interquartile range* or inter quartile range* or mortalit* or morbidit* or epidemiolog* or death* or dead* or fatal* or lethal*).tw,kf.	1889892
67	or/49-66 [Hospitalization, Recorded Health Events]	4202430
68	4 and 32 and 48 and 67 [NOx + Health + NOx Sources + Hospitalization]	2220

Database(s): Embase 1974 to 2018 February 13. Updated February 25, 2020.		
#	Searches	Results
1	nitrogen oxide/ or nitrogen dioxide/	19125
2	((nitrogen adj2 oxide*) or nitric oxide* or dinitrogen monoxide or dinitrogen oxide or hyponitrous acid anhydride or nitrogen hypoxide or nitrogen monoxide or nitrogen protoxide).tw,kw.	176192
3	(nitrogen dioxide or nitrogen peroxide or "NO_2" or "NOx").tw,kw.	32286
4	or/1-3 [NOx]	206118
5	((respira* or lung* or bronch* or cilia* or laryng* or pulmonar* or alveol* or aortic* or vascular* or cerebrovasc* or pericard* or endocard* or pleural or thorax or pneumo* or thoracic or trachea* or cardio* or cardia* or heart? or vascula* or atria* or ventric* or myocard* or coronary) adj2 (health or symptom* or infect* or disease* or disorder* or dysfunction* or syndrome* granuloma* or neoplas* or cancer* or tumor* or tumour* or ischemia* or mortal* or morbid* or fatal* or death? or admission* or hospital* or emergency or emergencies)).tw,kw.	1493275
6	respiratory tract disease/ or acute respiratory tract disease/ or exp bronchus disease/ or chronic aspecific respiratory tract disease/ or chronic respiratory tract disease/ or exp larynx disorder/ or exp lung disease/ or exp mediastinum disease/ or exp pleura disease/ or exp respiratory distress/ or exp respiratory failure/ or exp respiratory function disorder/ or exp respiratory tract hemorrhage/ or exp respiratory tract infection/ or exp respiratory tract inflammation/ or exp respiratory tract tumor/ or exp trachea disease/ or exp tracheobronchomalacia/ or obstructive airway disease/ or airway constriction/ or airway obstruction/ or exp bronchitis/ or bronchus obstruction/ or bronchus stenosis/ or chronic obstructive lung disease/ or exp lung emphysema/ or trachea stenosis/	2029890
7	(pleural empyema or whooping cough or pertussis or bronchiectasis).tw,kw.	43256
8	((bronchial adj2 (fistula* or hyperreactiv* or neoplasm* or spasm*)) or bronchitis or bronchiolitis or bronchogenic cyst* or bronchopneumonia* or bronchiectas?s or bronchospasm*).tw,kw.	68850
9	((laryng* adj2 (edema* or neoplasm* or nerve injur*)) or laryngitis or croup or supraglottitis or tuberculos* or laryngotracheobronchit*).tw,kw.	194835
10	acute chest syndrome*.tw,kw.	1694
11	cystic fibrosis.tw,kw.	57233
12	lung abscess*.tw,kw.	2634
13	blastomycos*.tw,kw.	2079
14	pneumonia*.tw,kw.	199482
15	pulmonary aspergillos*.tw,kw.	4048
16	alveolitis.tw,kw.	5461
17	pneumonitis.tw,kw.	19423
18	((chronic obstructive adj2 (pulmonary or airway or lung)) or chronic airflow obstruction or "COPD" or "COAD" or "AECOPD" or ("OPD" or obstructive pulmonary)).tw,kw.	101298

19	pulmonary edema*.tw,kw.	19376
20	pulmonary embolism*.tw,kw.	45158
21	respiratory distress syndrome*.tw,kw.	32110
22	bronchiogenic carcinoma*.tw,kw.	98
23	pulmonary nodule*.tw,kw.	9374
24	pulmonary nodule*.tw,kw.	9374
25	pulmonary sclerosing hemangioma*.tw,kw.	158
26	cardiovascular disease/ or exp cardiovascular infection/ or exp cardiovascular inflammation/ or cardiovascular symptom/ or exp cardiovascular system tumor/ or exp heart disease/ or exp vascular disease/	3244135
27	(NSTEMI or STEMI or cardiogenic shock* or ((cardiovascular or heart or myocardial) adj2 (stroke* or attack* or infarct* or event?)) or ((ischemic* or hemmorhagic or haemmorhagic) adj2 stroke?)).tw,kw.	378215
28	(angina or stenocardi*).tw,kw.	70011
29	(((heart or cardiac or myocardia*) adj2 (failure or decompensat*)) or cardiac edema or paroxysmal dyspnea or cardio-renal syndrome* or cardiorenal syndrome*).tw,kw.	251260
30	(arrhythmia* or dysrhythmia* or bradycard* or brugada syndrome* or commotio cordis or heart block* or long QT syndrome or parasystole or pre-excitation syndrome* or tachycardia* or ((atrial or ventricular) adj2 (fibrillation* or flutter)) or conduction disturbance*).tw,kw.	298942
31	or/5-30 [Health Outcomes]	5347836
32	exp air pollution/ or environmental exposure/ or occupational exposure/	283288
33	(air? adj3 (pollut* or environment* or outdoor* or outside*)).tw,kw.	42842
34	"traffic and transport"/ or exp aviation/ or exp motor vehicle/ or railway/ or ship/ or exp traffic/	190113
35	((distanc* or proximit* or close* or near) adj3 (traffic* or road* or highway* or transitway*)).tw,kw.	2959
36	traffic exposure*.tw,kw.	318
37	electric power plant/	7506
38	(power adj2 plant?).tw,kw.	9194
39	((motor* or vehic* or diesel* or gasoline* or fossil fuel* or crude oil* or coal? or natural gas or petrol*) adj4 (exhaust? or emission* or pollut* or vapor* or vapour* or volatile* or effluvia* or smoke* or fume? or combust* or haze? or smog* or nitrogen oxide* or NOx or nitrogen dioxide* or NO_2)).tw,kw.	18162
40	(automobile* or automotive* or autocar* or autobus* or taxi* or engine? or car or cars or truck* or bus or buses or bussing or highway* or high way* or motorway* or motor way or transitway* or road* or parkade* or parking* or carpark* or car park* or traffic or street* or motorcycl* or motorbi* or expressway* or parkway*).tw,kw.	171949

41	or/32-40 [NOx Sources]	573696
42	hospitalization/ or hospitalized adolescent/ or hospitalized child/ or hospitalized infant/	300849
43	exp medical care/	941022
44	exp ambulatory care/	45694
45	health care facility/ or air medical transport/ or exp ambulance/ or health center/ or exp hospital/ or exp isolation facility/ or pain clinic/ or exp pharmacy/ or secondary care center/ or tertiary care center/	1157185
46	(((clinic* or emergen* or doctor* or physician* or pediatric* or paediatric* or cardi* or respir* or healthcare or patient* or medical or primary care or health team or ambulatory care or "walk in") adj4 (admission* or admit* or readmission* or readmit* or discharg* or handoff or hand* off or transfer* or visit? or consult*)) or hospital*).tw,kw.	1824442
47	(("walk in" or clinic* or health care or healthcare or practitioner* or physician* or doctor* or medical or health care) adj3 (appointment* or booking* or office? or meeting*)).tw,kw.	27385
48	((CVD or STEMI or NSTEMI or "MI" or infarct* or cardio* or cardia* or myocard* or pericard* or coronary or respirat* or pulmonary or heart? or vascula* or atria* or ventric*) adj3 (event or events or inciden* or risk? or crisis or crises or symptom* or emergencies or emergency)).tw,kw.	454572
49	(primary adj2 care).tw,kw.	150558
50	epidemiological data/ or exp morbidity/ or exp mortality/ or epidemiological monitoring/ or epidemiology/	1258073
51	(IQR or interquartile range* or inter quartile range* or mortalit* or morbidit* or epidemiolog* or death* or dead* or fatal* or lethal*).tw,kw.	2577568
52	or/42-51 [Hospitalization, Recorded Health Events]	5718205
53	4 and 31 and 41 and 52 [NOx + Health + NOx Sources + Hospitalization]	2941

Database(s): Global Health 1973 to 2018 Week 05. Updated February 25, 2020.

#	Searches	Results
1	exp nitrogen oxides/	16898
2	((nitrogen adj2 oxide*) or nitric oxide* or dinitrogen monoxide or dinitrogen oxide or hyponitrous acid anhydride or nitrogen hypoxide or nitrogen monoxide or nitrogen protoxide).tw.	22301
3	(nitrogen dioxide or nitrogen peroxide or "NO_2" or "NOx").tw.	5264
4	or/1-3 [NOx]	26619
5	((respira* or lung* or bronch* or cilia* or laryng* or pulmonar* or alveol* or aortic* or vascular* or cerebrovasc* or pericard* or endocard* or pleural or thorax or pneumo* or thoracic or trachea* or cardio* or cardia* or heart? or vascula* or atria* or ventric* or myocard* or coronary) adj2 (health or symptom* or infect* or disease* or disorder* or dysfunction* or syndrome* granuloma* or neoplas* or cancer* or tumor* or tumour* or ischemia* or mortal* or morbid* or fatal* or death? or admission* or hospital* or emergency or emergencies)).tw.	199570

6	exp respiratory diseases/	105799
7	(pleural empyema or whooping cough or pertussis or bronchiectasis).tw.	7605
8	((bronchial adj2 (fistula* or hyperreactiv* or neoplasm* or spasm*)) or bronchitis or bronchiolitis or bronchogenic cyst* or bronchopneumonia* or bronchiectas?s or bronchospasm*).tw.	8132
9	((laryng* adj2 (edema* or neoplasm* or nerve injur*)) or laryngitis or croup or supraglottitis or tuberculos* or laryngotracheobronchit*).tw.	53261
10	acute chest syndrome*.tw.	74
11	cystic fibrosis.tw.	5687
12	lung abscess*.tw.	317
13	blastomycos*.tw.	9532
14	pneumonia*.tw.	57693
15	pulmonary aspergillos*.tw.	1856
16	alveolitis.tw.	614
17	pneumonitis.tw.	1756
18	((chronic obstructive adj2 (pulmonary or airway or lung)) or chronic airflow obstruction or "COPD" or "COAD" or "AECOPD" or ("OPD" or obstructive pulmonary)).tw.	7657
19	pulmonary edema*.tw.	652
20	pulmonary embolism*.tw.	971
21	respiratory distress syndrome*.tw.	2439
22	bronchiogenic carcinoma*.tw.	2
23	pulmonary nodule*.tw.	389
24	pancoast syndrome.tw.	8
25	pulmonary sclerosing hemangioma*.tw.	5
26	exp cardiovascular diseases/ or cardiovascular disorders/	99416
27	(NSTEMI or STEMI or cardiogenic shock* or ((cardiovascular or heart or myocardial) adj2 (stroke* or attack* or infarct* or event?)) or ((ischemic* or hemmorhagic or haemmorhagic) adj2 stroke?)).tw.	17599
28	(angina or stenocardi*).tw.	2216
29	(((heart or cardiac or myocardia*) adj2 (failure or decompensat*)) or cardiac edema or paroxysmal dyspnea or cardio-renal syndrome* or cardiorenal syndrome*).tw.	7725
30	(arrhythmia* or dysrhythmia* or bradycard* or brugada syndrome* or commotio cordis or heart block* or long QT syndrome or parasystole or pre-excitation syndrome* or tachycardia* or ((atrial or ventricular) adj2 (fibrillation* or flutter)) or conduction disturbance*).tw.	7400

31	or/5-30 [Health Outcomes]	338880
32	air pollution/ or exp air pollutants/	18643
33	(air? adj3 (pollut* or environment* or outdoor* or outside*)).tw.	20378
34	vehicle emissions/	3
35	exp vehicles/	5771
36	parking areas/ or motoring/	49
37	((distanc* or proximit* or close* or near) adj3 (traffic* or road* or highway* or transitway*)).tw.	1014
38	traffic exposure*.tw.	146
39	(power adj2 plant?).tw.	1875
40	((motor* or vehic* or diesel* or gasoline* or fossil fuel* or crude oil* or coal? or natural gas or petrol*) adj4 (exhaust? or emission* or pollut* or vapor* or vapour* or volatile* or effluvia* or smoke* or fume? or combust* or haze? or smog* or nitrogen oxide* or NOx or nitrogen dioxide* or NO_2)).tw.	2921
41	(automobile* or automotive* or autocar* or autobus* or taxi* or engine? or car or cars or truck* or bus or buses or bussing or highway* or high way* or motorway* or motor way or transitway* or road* or parkade* or parking* or carpark* or car park* or traffic or street* or motorcycl* or motorbi* or expressway* or parkway*).tw.	28217
42	or/32-41 [NOx Sources]	50963
43	hospital stay/ or hospital admission/ or hospital care/	11445
44	primary health care/	10072
45	health centres/ or hospitals/	35142
46	(((clinic* or emergen* or doctor* or physician* or pediatric* or paediatric* or cardi* or respir* or healthcare or patient* or medical or primary care or health team or ambulatory care or "walk in") adj4 (admission* or admit* or readmission* or readmit* or discharg* or handoff or hand* off or transfer* or visit? or consult*)) or hospital*).tw.	237731
47	(("walk in" or clinic* or health care or healthcare or practitioner* or physician* or doctor* or medical or health care) adj3 (appointment* or booking* or office? or meeting*)).tw.	2385
48	((CVD or STEMI or NSTEMI or "MI" or infarct* or cardio* or cardia* or myocard* or pericard* or coronary or respirat* or pulmonary or heart? or vascula* or atria* or ventric*) adj3 (event or events or inciden* or risk? or crisis or crises or symptom* or emergencies or emergency)).tw.	53310
49	(primary adj2 care).tw.	22073
50	morbidity/ or mortality/	96216
51	epidemiology/	250285
52	(IQR or interquartile range* or inter quartile range* or mortalit* or morbidit* or epidemiolog* or death* or dead* or fatal* or lethal*).tw.	531399
53	or/43-52 [Hospitalization, Recorded Health Events]	723805

54	4 and 31 and 42 and 53 [NOx + Health + NOx Sources + Hospitalization]	1184
Database(s): EBM Reviews - Cochrane Central Register of Controlled Trials January 2018. Updated February 25, 2020.		
**#**	**Searches**	**Results**
1	exp nitrogen oxides/	3079
2	((nitrogen adj2 oxide*) or nitric oxide* or dinitrogen monoxide or dinitrogen oxide or hyponitrous acid anhydride or nitrogen hypoxide or nitrogen monoxide or nitrogen protoxide).tw,kf.	4826
3	(nitrogen dioxide or nitrogen peroxide or "NO_2" or "NOx").tw,kf.	473
4	or/1-3 [NOx]	6528
5	((respira* or lung* or bronch* or cilia* or laryng* or pulmonar* or alveol* or aortic* or vascular* or cerebrovasc* or pericard* or endocard* or pleural or thorax or pneumo* or thoracic or trachea* or cardio* or cardia* or heart? or vascula* or atria* or ventric* or myocard* or coronary) adj2 (health or symptom* or infect* or disease* or disorder* or dysfunction* or syndrome* granuloma* or neoplas* or cancer* or tumor* or tumour* or ischemia* or mortal* or morbid* or fatal* or death? or admission* or hospital* or emergency or emergencies)).tw,kf.	82383
6	exp Respiratory Tract Infections/	10042
7	exp Respiratory Tract Diseases/	41598
8	(pleural empyema or whooping cough or pertussis or bronchiectasis).tw,kf.	1737
9	((bronchial adj2 (fistula* or hyperreactiv* or neoplasm* or spasm*)) or bronchitis or bronchiolitis or bronchogenic cyst* or bronchopneumonia* or bronchiectas?s or bronchospasm*).tw,kf.	4694
10	((laryng* adj2 (edema* or neoplasm* or nerve injur*)) or laryngitis or croup or supraglottitis or tuberculos* or laryngotracheobronchit*).tw,kf.	3914
11	acute chest syndrome*.tw,kf.	98
12	cystic fibrosis.tw,kf.	3705
13	lung abscess*.tw,kf.	35
14	blastomycos*.tw,kf.	6
15	pneumonia*.tw,kf.	7509
16	pulmonary aspergillos*.tw,kf.	54
17	alveolitis.tw,kf.	95
18	pneumonitis.tw,kf.	771
19	((chronic obstructive adj2 (pulmonary or airway or lung)) or chronic airflow obstruction or "COPD" or "COAD" or "AECOPD" or ("OPD" or obstructive pulmonary)).tw,kf.	12648
20	pulmonary edema*.tw,kf.	489
21	pulmonary embolism*.tw,kf.	1975

22	respiratory distress syndrome*.tw,kf.	2162
23	bronchiogenic carcinoma*.tw,kf.	0
24	pulmonary nodule*.tw,kf.	143
25	pancoast syndrome.tw,kf.	0
26	pulmonary sclerosing hemangioma*.tw,kf.	0
27	exp Cardiovascular Diseases/	78173
28	(NSTEMI or STEMI or cardiogenic shock* or ((cardiovascular or heart or myocardial) adj2 (stroke* or attack* or infarct* or event?)) or ((ischemic* or hemmorhagic or haemmorhagic) adj2 stroke?)).tw,kf.	30219
29	(angina or stenocardi*).tw,kf.	8324
30	(((heart or cardiac or myocardia*) adj2 (failure or decompensat*)) or cardiac edema or paroxysmal dyspnea or cardio-renal syndrome* or cardiorenal syndrome*).tw,kf.	16229
31	(arrhythmia* or dysrhythmia* or bradycard* or brugada syndrome* or commotio cordis or heart block* or long QT syndrome or parasystole or pre-excitation syndrome* or tachycardia* or ((atrial or ventricular) adj2 (fibrillation* or flutter)) or conduction disturbance*).tw,kf.	16928
32	or/5-31 [Health Outcomes]	202306
33	air/	497
34	air pollution/ or environmental exposure/ or inhalation exposure/ or exp occupational exposure/ or smog/	1152
35	environmental pollution/	11
36	exp air pollutants/	481
37	(air? adj3 (pollut* or environment* or outdoor* or outside*)).tw,kf.	522
38	Vehicle Emissions/	71
39	Automobile Driving/	730
40	Parking Facilities/	2
41	exp transportation/	595
42	((distanc* or proximit* or close* or near) adj3 (traffic* or road* or highway* or transitway*)).tw,kf.	45
43	traffic exposure*.tw,kf.	5
44	Power Plants/	22
45	(power adj2 plant?).tw,kf.	16
46	((motor* or vehic* or diesel* or gasoline* or fossil fuel* or crude oil* or coal? or natural gas or petrol*) adj4 (exhaust? or emission* or pollut* or vapor* or vapour* or volatile* or effluvia* or	207

	smoke* or fume? or combust* or haze? or smog* or nitrogen oxide* or NOx or nitrogen dioxide* or NO_2)).tw,kf.	
47	(automobile* or automotive* or autocar* or autobus* or taxi* or engine? or car or cars or truck* or bus or buses or bussing or highway* or high way* or motorway* or motor way or transitway* or road* or parkade* or parking* or carpark* or car park* or traffic or street* or motorcycl* or motorbi* or expressway* or parkway*).tw,kf.	3379
48	or/33-47 [NOx Sources]	6458
49	exp hospitalization/	11743
50	child hospitalized/ or adolescent hospitalized/	107
51	exp patient care/	36584
52	exp primary health care/	4240
53	exp hospital departments/	3022
54	exp hospitals/	3107
55	health facilities/	62
56	ambulatory care facilities/	403
57	Physicians' Offices/	22
58	(((clinic* or emergen* or doctor* or physician* or pediatric* or paediatric* or cardi* or respir* or healthcare or patient* or medical or primary care or health team or ambulatory care or "walk in") adj4 (admission* or admit* or readmission* or readmit* or discharg* or handoff or hand* off or transfer* or visit? or consult*)) or hospital*).tw,kf.	109946
59	(("walk in" or clinic* or health care or healthcare or practitioner* or physician* or doctor* or medical or health care) adj3 (appointment* or booking* or office? or meeting*)).tw,kf.	6315
60	((CVD or STEMI or NSTEMI or "MI" or infarct* or cardio* or cardia* or myocard* or pericard* or coronary or respirat* or pulmonary or heart? or vascula* or atria* or ventric*) adj3 (event or events or inciden* or risk? or crisis or crises or symptom* or emergencies or emergency)).tw,kf.	42028
61	(primary adj2 care).tw,kf.	12981
62	exp morbidity/	13547
63	exp mortality/	11696
64	exp epidemiology/	43
65	epidemiological monitoring/	29
66	(IQR or interquartile range* or inter quartile range* or mortalit* or morbidit* or epidemiolog* or death* or dead* or fatal* or lethal*).tw,kf.	94834
67	or/49-66 [Hospitalization, Recorded Health Events]	246028
68	4 and 32 and 48 and 67 [NOx + Health + NOx Sources + Hospitalization]	51

Toxline (1900-2018). Updated February 25, 2020.				
**Search**	**Database**	**Query**	**Time**	**Result**
# 5	toxline	(#1 AND #2 AND #3 AND #4) AND NOT PubMed [org] AND NOT pubdart [org]	09:12:37	39
# 4	toxline	("nitrogen dioxide" OR "stikstofdioxyde dutch " "stickstoffdioxid german " "nitrogen peroxide" OR nitrito OR "azoto italian " "azote french " OR 10102-44-0 [rn]) [not] PubMed [org] [not] pubdart [org]	09:10:17	5874
# 3	toxline	(exhaust OR emission OR pollute OR pollutant OR pollution OR automobile OR automotive OR autocar OR autobus OR taxi OR engine OR car OR cars OR truck OR bus OR buses OR bussing OR highway OR "high way" OR motorway OR "motor way" OR transitway OR road OR parkade OR parking OR carpark OR "car park" OR traffic OR street OR motorcycle OR motorbike OR expressway OR parkway OR "power plant") AND NOT PubMed [org] AND NOT pubdart [org]	09:08:33	500000
# 2	toxline	(clinic OR emergency OR emergencies OR doctor OR physician OR healthcare OR patient OR medical OR "primary care" OR "health team" OR "ambulatory care" OR "walk in" OR hospital OR event OR incident OR risk OR crisis OR crises) AND NOT PubMed [org] AND NOT pubdart [org]	09:06:13	500000
# 1	toxline	(respiratory OR lung OR bronchial OR cilia OR laryngeal OR pulmonary OR alveola OR aortic OR vascular OR cerebrovascular OR pericardial OR endocardial OR pleural OR thorax OR thoracic OR tracheal OR cardiopulmonary OR cardiovascular OR cardiac OR heart OR atrial OR ventricle OR ventricular OR myocardial OR coronary) AND NOT PubMed [org] AND NOT pubdart [org]	09:04:57	500000
