# Supplementary material for: Systematic review and meta-analysis of cohort studies of long term outdoor nitrogen dioxide exposure and mortality
Source: PLoS One. 2021 Feb 4;16(2):e0246451. doi: 10.1371/journal.pone.0246451 (PMC7861378; doi:10.1371/journal.pone.0246451)
Supplement: S2 Table — (PDF) [file pone.0246451.s010.pdf]

Online supplementary table S2, Navigation Guide Criteria for Overall Quality and Strength of Evidence[a]

A) Overall Quality

Downgrading Factors	Summary of criteria for downgrading
Risk of bias	Study limitations – a substantial risk of bias across body of evidence
Indirectness	Evidence was not directly comparable to the question of interest (i.e., population, exposure, comparator, outcome)
Inconsistency	Widely different estimates of effect in similar populations (heterogeneity or variability in results)
Imprecision	Studies had few participants and few events (wide confidence intervals as judged by reviewers)
Publication Bias	Studies missing from body of evidence, resulting in an over or underestimate of true effects from exposure
**Upgrading Factors**	**Summary of criteria for upgrading**
Large magnitude of effect	Upgraded if modeling suggested confounding alone unlikely to explain associations with large effect estimate as judged by reviewers
Dose response	Upgraded if consistent relationship between dose and response in one or multiple studies, and/or dose response across studies
Confounding minimizes effect	Upgraded if consideration of all plausible residual confounders or biases would underestimate the effect or suggest a spurious effect when results show no effect

B) Strength of evidence

Strength rating	Definition
Sufficient evidence of toxicity	A positive relationship is observed between exposure and outcome where chance, bias, and confounding can be ruled out with reasonable confidence. The available evidence includes results from one or more well-designed, well conducted studies, and the conclusion is unlikely to be strongly affected by the results of future studies.
Limited Evidence of Toxicity	A positive relationship is observed between exposure and outcome where chance, bias, and confounding cannot be ruled out with reasonable confidence. Confidence in the relationship is constrained by such factors as: the number, size, or quality of individual studies, or inconsistency of findings across individual studies. As more information becomes available, the observed effect could change, and this change may be large enough to alter the conclusion.
Inadequate Evidence of Toxicity	The available evidence is insufficient to assess effects of the exposure. Evidence is insufficient because of: the limited number or size of studies, low quality of individual studies, or inconsistency of findings across individual studies. More information may allow an assessment of effects.
Evidence of Lack of Toxicity	No relationship is observed between exposure and outcome, and chance, bias and confounding can be ruled out with reasonable confidence. The available evidence includes consistent results from more than one well-designed, well conducted study at the full range of exposure levels that humans are known to encounter, and the conclusion is unlikely to be strongly affected by the results of future studies. The conclusion is limited to the age at exposure and/or other conditions and levels of exposure studied.

[a]Adapted/reproduced from: Lam J, Sutton P, McPartland J, Davidson L, Daniels N, Sen S, et al. Applying the Navigation Guide Systematic Review Methodology Case Study #5 Association between Developmental Exposures to PBDEs and Human Neurodevelopment: A Systematic Review of the Evidence Protocol April 2015 [Internet]. [cited 2019 Aug 18]. Available from: http://www.crd.york.ac.uk/PROSPEROFILES/17890_PROTOCOL_20150322.pdf
