# Supplementary material for: Systematic review and meta-analysis of cohort studies of long term outdoor nitrogen dioxide exposure and mortality
Source: PLoS One. 2021 Feb 4;16(2):e0246451. doi: 10.1371/journal.pone.0246451 (PMC7861378; doi:10.1371/journal.pone.0246451)
Supplement: S4 Table — (PDF) [file pone.0246451.s012.pdf]

Online supplementary table S4. Characteristics of primary studies (shading indicates reason for exclusion from pooled analysis; yellow indicates exclusion in favour of other analysis of same cohort of greater duration or geographic scope; blue indicates results were provided only from multi-pollutant models [4], or there were too few studies of the outcome [16, 17, 26, 36], green indicates exclusion from pooled analysis in favour of ESCAPE[a] multi-cohort analysis)

Study	Cohort[a]	Location	Country/ Region	Start	End	N	Exposure	Minimum Age	Cause of Death[b]	Mean NO_2 (ppb)[c]
Crouse 2015 [1]	CanCHEC 1991	Canada	Canada	1991	2006	2,521,525	model	25	AC/NC, CEV, COPD, CV, IHD, LC, RES	11.6
Crouse 2015 [2]	CanCHEC 1991	10 Canadian cities	Canada	1991	2006	735,590	model	25	AC/NC, CEV, CV, IHD, RES	25.2
Weichenthal 2017 [3]	CanCHEC 2001	Canada	Canada	2001	2011	2,448,500	model	25	AC/NC, CV, RES	11.5
Villeneuve 2013 [4]	OTCS	Toronto, Canada	Canada	1982	2004	58,760	model	35	AC/NC, C, CV, LC, RES	28.9
Chen 2013 [5]	OTCS	Toronto, Hamilton, Windsor, Canada	Canada	1982	2004	205,440	model	35	CEV, CV, IHD	23.3
Jerrett 2009 [6]	Toronto Western Hospital	Toronto, Canada	Canada	1992	2002	2,360	model	NA	AC/NC, CV, RES	23.1
Gan 2011 [7]	Vancouver CHD	Vancouver, Canada	Canada	1999	2002	452,735	model	45	IHD	17.1
Gan 2013 [8]	Vancouver COPD	Vancouver, Canada	Canada	1999	2002	467,994	model	45	COPD	17.1
Abbey 1999 [9]	AHSMOG	California	United States	1977	1992	6,338	monitor	27	AC/NC, CPD, LC, RES	36.8
Chen 2005 [10]	AHSMOG	San Francisco, South Coast, San Diego	United States	1977	1998	3,239	monitor	25	IHD	34.9
Eckel 2016 [11]	California cancer	California	United States	1988	2009	352,053	monitor	NA	AC/NC, LC	21.9
Hartiala 2016 [12]	Cleveland Clinic GeneBank	Ohio	United States	2001	2010	4,363	monitor	NA	AC/NC	18.2
Jerrett 2013 [13]	CPS II	California	United States	1982	2000	73,711	model	30	AC/NC, CEV, CV, IHD, LC, RES	12.3
Pope 2002 [14]	CPS II	United States	United States	1982	1998	500,000	monitor	30	AC/NC, CPD, LC	21.4
Krewski 2009 [15]	CPS II	United States	United States	1982	2000	406,917	monitor	30	AC/NC, CPD, IHD, LC	27.9
McKean-Cowdin 2009 [16]	CPS II	United States	United States	1982	2000	527,123	monitor	30	BC	21.3
Turner 2017 [17]	CPS II	United States	United States	1982	2004	623,048	model	30	CC	11.6
Turner 2016 [18]	CPS II	United States	United States	1982	2004	669,046	model	30	AC/NC, CEV, COPD, CV, IHD, LC, RES	11.6
Lipsett 2011 [19]	CTS	California	United States	1996	2005	101,784	monitor	30	AC/NC, CEV, CV, IHD, LC, RES	33.6
Krewski 2000 [20]	Harvard Six Cities	6 US cities	United States	1974	1989	8,111	monitor	25	AC/NC, CPD, LC	15.0
Eum 2019 [21]	Medicare	United States	United States	2000	2008	14,100,000	monitor	65	AC/NC, CEV, COPD, CV, HF, IHD, LC, PN, RES	14.8
Lefler 2019 [22]	NHIS	United States	United States	1987	2015	635,539	model	18	AC/NC,CPD	10.7
Hart 2013 [23]	NHS	United States	United States	1988	2008	93,807	model	30	AC/NC	13.9
Lim 2019 [24]	NIH-AARP	6 US states	United States	1995	2011	548,845	model	50	CA,CEV,CV,IHD	13.3
Lim 2019 [25]	NIH-AARP	6 US states	United States	1995	2011	548,780	model	50	AC/NC,CA,CEV,COPD,	11.1

Online supplementary table S4. Characteristics of primary studies (shading indicates reason for exclusion from pooled analysis; yellow indicates exclusion in favour of other analysis of same cohort of greater duration or geographic scope; blue indicates results were provided only from multi-pollutant models [4], or there were too few studies of the outcome [16, 17, 26, 36], green indicates exclusion from pooled analysis in favour of ESCAPE[a] multi-cohort analysis)

Study	Cohort[a]	Location	Country/ Region	Start	End	N	Exposure	Minimum Age	Cause of Death[b]	Mean NO_2 (ppb)[c]
									CV,HF,IHD,LC,PN,RES	
Lim 2018 [26]	NIH-AARP	6 US states	United States	1995	2011	549,735	model	50	DB	12.2
Hart 2011 [27]	TrIPS	United States	United States	1985	2000	39,948	model	NA	AC/NC, COPD, CV, IHD, LC, RES	14.2
Lipfert 2006 [28]	WU/EPRI Veterans	United States	United States	1997	2001	28,635	monitor	NA	AC/NC	13.6
Lipfert 2006 [29]	WU/EPRI Veterans	United States	United States	1989	2001	41,598	monitor	NA	AC/NC	21.5
Lipfert 2009 [30]	WU/EPRI Veterans	United States	United States	1976	2001	67,938	model	NA	AC/NC	11.1
Lipfert 2019 [31]	WU/EPRI Veterans	United States	United States	1976	2001	67,938	monitor	NA	AC/NC	27.1
Lipfert 2018 [32]	WU/EPRI Veterans	United States	United States	1976	2001	67,938	monitor	NA	AC/NC	27.0
Bauleo 2019 [33]	Civitavecchia	Civitavecchia, Italy	Europe	1996	2013	71,362	model	18	AC/NC,CD,CEV,COPD, CV,IHD,LC,ND,RES	1.8
Carey 2013 [34]	CPRD	England	Europe	2003	2007	830,429	model	40	AC/NC, CV, LC, RES	12.0
Sifaki-Pistolla 2017 [35]	Crete lung cancer	Crete, Greece	Europe	1992	2014	5,057	model	NA	LC	9.0
Raaschou-Nielsen 2012 [36]	DDCH	Copenhagen and Aarhus, Denmark	Europe	1993	2009	52,061	model	50	DB	9.0
Raaschou-Nielsen 2012 [37]	DDCH	Copenhagen and Aarhus, Denmark	Europe	1993	2009	52,061	model	50	AC/NC, CEV, CV, IHD	9.0
Hvidtfeldt 2019 [38]	DDCH	Copenhagen and Aarhus, Denmark	Europe	1993	2015	49,564	model	50	AC/NC,CV,LC,RES[d]	14.0
Sørensen 2014 [39]	DDCH	Copenhagen and Aarhus, Denmark	Europe	1993	2009	51,569	model	50	CEV	9.7
Andersen 2012 [40]	DDCH	Copenhagen and Aarhus, Denmark	Europe	1993	2006	52,215	model	50	CEV	10.0
Fischer 2015 [41]	DUELS	Netherlands	Europe	2004	2011	7,218,363	model	30	AC/NC, CV, LC, RES	17.0
Dimakopoulou 2014 [42]	ESCAPE	Europe	Europe	1985	various	307,553	model	NA	RES	NA
Beelen 2014 [43]	ESCAPE	Europe	Europe	1985	various	367,251	model	NA	AC/NC	NA
Beelen 2014 [44]	ESCAPE	Europe	Europe	1985	various	367,383	model	NA	CEV, CV, IHD	NA
Bentayeb 2015 [45]	GAZEL	France	Europe	1989	2013	20,327	model	NA	AC/NC, CV, RES	14.9
Tonne 2013 [46]	MINAP	England and Wales	Europe	2004	2010	154,204	model	25	AC/NC	10.0
Tonne 2016 [47]	MINAP	London, United Kingdom	Europe	2003	2010	18,138	model	25	AC/NC	19.7
Stockfelt 2015 [48]	MPPS	Gothenburg, Sweden	Europe	1970	2007	7,494	model	NA	AC/NC, CEV, CV, IHD, RES	12.7

Online supplementary table S4. Characteristics of primary studies (shading indicates reason for exclusion from pooled analysis; yellow indicates exclusion in favour of other analysis of same cohort of greater duration or geographic scope; blue indicates results were provided only from multi-pollutant models [4], or there were too few studies of the outcome [16, 17, 26, 36], green indicates exclusion from pooled analysis in favour of ESCAPE[a] multi-cohort analysis)

Study	Cohort[a]	Location	Country/ Region	Start	End	N	Exposure	Minimum Age	Cause of Death[b]	Mean NO_2 (ppb)[c]
Dehbi 2017 [49]	NSHD, SABRE	London, United Kingdom	Europe	1989	2015	7,529	model	40	CV	23.7
Beelen 2008 [50]	NLCS	Netherlands	Europe	1986	1996	117,528	monitor	55	CD, CEV, CV, HF, IHD	NA
Beelen 2008 [51]	NLCS	Netherlands	Europe	1986	1996	117,528	monitor	55	AC/NC, CV, LC, RES	19.6
Brunekreef 2009 [52]	NLCS	Netherlands	Europe	1986	1996	105,296	model	55	AC/NC, CV, LC, RES	20.5
Hoek 2002 [53]	NLCS	Netherlands	Europe	1986	1994	4,492	monitor	55	AC/NC, CPD, LC	19.5
Nafstad 2004 [54]	Norwegian men	Oslo, Norway	Europe	1972	1998	15,966	model	40	AC/NC, CEV, IHD, LC, RES	4.4
Naess 2007 [55]	Oslo	Oslo, Norway	Europe	1992	1998	143,842	model	51	COPD, CV, LC	20.7
Filleul 2005 [56]	PAARC	7 French cities	Europe	1974	2001	14,284	monitor	25	AC/NC, CPD, LC	NA
Klompmaker 2020 [57]	PHM	Netherlands	Europe	2013	2017	339,633	model	30	AC/NC,CV,LC,ND,RES	12.9
Cesaroni 2012 [58]	RoLS	Rome, Italy	Europe	2001	2006	684,000	model	45	AC/NC	24.1
Cesaroni 2013 [59]	RoLS	Rome, Italy	Europe	2001	2010	1,265,058	model	30	AC/NC, CEV, CV, IHD, LC, RES	23.2
Rosenlund 2008 [60]	Rome	Rome, Italy	Europe	1998	2005	6,513	model	35	AC	NA
Schikowski 2007 [61]	SALIA	North Rhine-Westphalia, Germany	Europe	1985	2003	4,750	monitor	55	CV	20.7
Gehring 2006 [62]	SALIA	North Rhine-Westphalia, Germany	Europe	1985	2003	4,752	monitor	50	AC/NC, CPD	20.7
Heinrich 2013 [63]	SALIA	North Rhine-Westphalia, Germany	Europe	1985	2008	4,752	monitor	55	AC/NC, CPD, LC, RES	20.7
Nieuwenhuijsen 2018 [64]	SIDIAP	Barcelona, Spain	Europe	2010	2014	792,649	model	18	AC	
Maheswaran 2010 [65]	SLSR	South London	Europe	1995	2006	3,320	model	NA	AC/NC, CEV	21.8
Desikan et al. 2016 [66]	SLSR	South London	Europe	1995	2012	1,800	model	NA	AC/NC	23.7
Heritier 2019 [67]	SNC	Switzerland	Europe	2000	2008	4,404,046	model	30	MI	14.7
Cao 2011 [68]	CNHS	China	Other	1991	2000	70,947	monitor	40	AC/NC, CV, LC, RES	15.1
Dirgawati 2019 [69]	HIMS	Perth, Australia	Other	1996	2012	11,483	model	65	AC/NC,CEV	7.1
Yang 2018 [70]	HKEHC	Hong Kong	Other	1998	2011	61,386	model	65	AC/NC,CEV,COPD,CV, IHD,PN,RES	53.9
Barratt 2018 [71]	HKEHC	Hong Kong	Other	1998	2014	60,548	model	65	AC/NC,CEV,COPD,CV, IHD,PN,RES	30.8
Kim 2017 [72]	NHIS-NSC	Seoul, Korea	Other	2007	2013	136,094	monitor	18	AC/NC, CV, MI	34.5

Online supplementary table S4. Characteristics of primary studies (shading indicates reason for exclusion from pooled analysis; yellow indicates exclusion in favour of other analysis of same cohort of greater duration or geographic scope; blue indicates results were provided only from multi-pollutant models [4], or there were too few studies of the outcome [16, 17, 26, 36], green indicates exclusion from pooled analysis in favour of ESCAPE[a] multi-cohort analysis)

Study	Cohort[a]	Location	Country/ Region	Start	End	N	Exposure	Minimum Age	Cause of Death[b]	Mean NO_2 (ppb)[c]
Chen 2016 [73]	Northern Chinese	Tianjin, Shenyang, Taiyuan, Rizhao, China	Other	1998	2009	39,054	monitor	23	AC/NC, LC	21.6
Yorifuji 2010 [74]	SEC	Shizuoka, Japan	Other	1999	2006	13,444	model	65	AC/NC, CEV, COPD, CV, IHD, LC, RES	19.0
Yorifuji 2013 [75]	SEC	Shizuoka, Japan	Other	1999	2009	13,412	model	65	AC/NC, CEV, COPD, CPD, CV, IHD, LC, RES	11.7
Dong 2012 [76]	Shenyang	Shenyang, China	Other	1998	2009	9,941	monitor	25	RES	24.4
Zhang 2011 [77]	Shenyang	Shenyang, China	Other	1998	2009	9,941	monitor	25	CEV, CV	24.4
Tseng 2015 [78]	Taiwan civil servants	Taipei, Taiwan	Other	1989	2008	43,227	monitor	NA	CV	NA
Katanoda 2011 [79]	Three Prefecture	Miyagi, Aichi, Osaka, Japan	Other	1983	1995	63,520	monitor	NA	LC, RES	NA

[a] AHSMOG, Adventist Health and smog; CanCHEC, Canadian Census Health and Environment Cohort; Cleveland Clinic GeneBank study; CPS-II, Cancer Prevention Study-II; CNHS, China National Hypertension Survey; CPRD, Clinical Practice Research Datalink; CTS, California Teachers Study; DDCH, Danish Diet, Cancer and Health; DUELS, Dutch Environmental Longitudinal Study; ESCAPE, European Study of Cohorts for Air Pollution Effects; GAZEL, GAZ and ELectricité; HIMS, Health in Men Study; HKEHC, Hong Kong Elderly Health Centres; JPHC, Japan Public Health Centre; MINAP, Myocardial Ischaemia National Audit Project; MPPS, Multifactor Primary Prevention Study; NHIS, National Health Interview Survey; NHIS-NSC, National Health Insurance Service–National Sample Cohort; NIH-AARP, National Institutes of Health, American Association of Retired Persons; NSHD, National Survey of Health and Development; NLCS, Netherlands Cohort study on Diet and Cancer; NHS, Nurses Health Study; OTCS, Ontario Tax Cohort study; PAARC, Pollution Atmosphérique et Affections Respiratoires Chroniques; PHM, Public Health Monitor; RoLS, Rome Longitudinal Study; SEC, Shizuoka elderly cohort; SIDIAP, Sistema d'Informació pel Desenvolupament de la Investigació en Atenció Primària; SLSR, South London Stroke Register; SABRE, Southall And Brent Revisited; SALIA, Study on the influence of Air pollution on Lung function, Inflammation and Aging; SNC, Swiss National Cohort; TRiPS, the Trucking Industry Particle Study; WU/EPRI, Washington University/Electric Power Research Institute.

[b] AC/NC, All cause/Natural cause; BC, brain cancer; C, cardiac; CA, cardiac arrest; CC, colorectal cancer; CPD, cardiopulmonary disease; CV, cardiovascular; CEV, cerebrovascular; COPD, chronic obstructive pulmonary disease; DB, diabetes; IHD, ischemic heart disease; LC, lung cancer; MI, myocardial infarction; ND, neurologic disease; PN, pneumonia; RES, respiratory.

[c] In some cases estimated from median and/or nitrogen oxides.

[d] Only results for lung cancer were included in pooled analysis. Other outcomes captured through ESCAPE multi-cohort analysis.

1. Crouse DL, Peters PA, Hystad P, Brook JR, van Donkelaar A, Martin RV, et al. Ambient PM2.5, O3, and NO2 exposures and associations with mortality over 16 years of follow-up in the canadian census health and environment cohort (CanCHEC). Environ Health Perspect. 2015;123: 1180–1186. doi:10.1289/ehp.1409276

2. Crouse DL, Peters PA, Villeneuve PJ, Proux M-O, Shin HH, Goldberg MS, et al. Within- and between-city contrasts in nitrogen dioxide and mortality in 10 Canadian cities; A subset of the Canadian Census Health and Environment Cohort (CanCHEC). J Expo Sci Environ Epidemiol. 2015;25: 482–489. doi:10.1038/jes.2014.89

3. Weichenthal S, Crouse DL, Pinault L, Godri-Pollitt K, Lavigne E, Evans G, et al. Oxidative burden of fine particulate air pollution and risk of cause-specific mortality in the Canadian Census Health and Environment Cohort (CanCHEC). Environ Res. 2016;146: 92–99. doi:10.1016/j.envres.2015.12.013

4. Villeneuve PJ, Jerrett M, Su J, Burnett RT, Chen H, Brook J, et al. A cohort study of intra-urban variations in volatile organic compounds and mortality, Toronto, Canada. Environ Pollut. 2013;183: 30–39. doi:10.1016/j.envpol.2012.12.022

5. Chen H, Goldberg MS, Burnett RT, Jerrett M, Wheeler AJ, Villeneuve PJ. Long-term exposure to traffic-related air pollution and cardiovascular mortality. Epidemiology. 2013;24: 35–43. doi:10.1097/EDE.0b013e318276c005

6. Jerrett M, Finkelstein MM, Brook JR, Arain MA, Kanaroglou P, Stieb DM, et al. A cohort study of traffic-related air pollution and mortality in Toronto, Ontario, Canada. Environ Health Perspect. 2009;117: 772–777. doi:10.1289/ehp.11533

7. Gan WQ, Koehoorn M, Davies HW, Demers PA, Tamburic L, Brauer M. Long-Term Exposure to Traffic-Related Air Pollution and the Risk of Coronary Heart Disease Hospitalization and Mortality. Environ Health Perspect. 2011;119: 501–507. doi:10.1289/ehp.1002511

8. Gan WQ, FitzGerald JM, Carlsten C, Sadatsafavi M, Brauer M. Associations of Ambient Air Pollution with Chronic Obstructive Pulmonary Disease Hospitalization and Mortality. Am J Respir Crit Care Med. 2013;187: 721–727. doi:10.1164/rccm.201211-2004OC

9. Abbey DE, Nishino N, McDONNELL WF, Burchette RJ, Knutsen SF, Lawrence Beeson W, et al. Long-Term Inhalable Particles and Other Air Pollutants Related to Mortality in Nonsmokers. Am J Respir Crit Care Med. 1999;159: 373–382. doi:10.1164/ajrccm.159.2.9806020

10. Chen LH, Knutsen SF, Shavlik D, Beeson WL, Petersen F, Ghamsary M, et al. The Association between Fatal Coronary Heart Disease and Ambient Particulate Air Pollution: Are Females at Greater Risk? Environ Health Perspect. 2005;113: 1723–1729. doi:10.1289/ehp.8190

11. Eckel SP, Cockburn M, Shu Y-H, Deng H, Lurmann FW, Liu L, et al. Air pollution affects lung cancer survival. Thorax. 2016;71: 891–898. doi:10.1136/thoraxjnl-2015-207927

12. Hartiala J, Breton CV, Tang WHW, Lurmann F, Hazen SL, Gilliland FD, et al. Ambient Air Pollution Is Associated With the Severity of Coronary Atherosclerosis and Incident Myocardial Infarction in Patients Undergoing Elective Cardiac Evaluation. J Am Heart Assoc. 2016;5: e003947. doi:10.1161/JAHA.116.003947

13. Jerrett M, Burnett RT, Beckerman BS, Turner MC, Krewski D, Thurston G, et al. Spatial Analysis of Air Pollution and Mortality in California. Am J Respir Crit Care Med. 2013;188: 593–599. doi:10.1164/rccm.201303-0609OC

14. Pope III CA. Lung Cancer, Cardiopulmonary Mortality, and Long-term Exposure to Fine Particulate Air Pollution. JAMA. 2002;287: 1132. doi:10.1001/jama.287.9.1132

15. Krewski D, Jerrett M, Burnett RT, Ma R, Hughes E, Shi Y, et al. Extended follow-up and spatial analysis of the American Cancer Society study linking particulate air pollution and mortality. Res Rep Health Eff Inst. 2009;140.

16. McKean-Cowdin R, Calle EE, Peters JM, Henley J, Hannan L, Thurston GD, et al. Ambient air pollution and brain cancer mortality. Cancer Causes Control. 2009;20: 1645–1651. doi:10.1007/s10552-009-9412-1

17. Turner MC, Krewski D, Diver WR, Pope CA, Burnett RT, Jerrett M, et al. Ambient Air Pollution and Cancer Mortality in the Cancer Prevention Study II. Environ Health Perspect. 2017;125: 087013. doi:10.1289/EHP1249

18. Turner MC, Jerrett M, Pope CA, Krewski D, Gapstur SM, Diver WR, et al. Long-Term Ozone Exposure and Mortality in a Large Prospective Study. Am J Respir Crit Care Med. 2016;193: 1134–1142. doi:10.1164/rccm.201508-1633OC

19. Lipsett MJ, Ostro BD, Reynolds P, Goldberg D, Hertz A, Jerrett M, et al. Long-Term Exposure to Air Pollution and Cardiorespiratory Disease in the California Teachers Study Cohort. Am J Respir Crit Care Med. 2011;184: 828–835. doi:10.1164/rccm.201012-2082OC

20. Krewski D, Burnett RT, Goldberg M, Hoover K, Siemiatycki J, Jerrett M, et al. Reanalysis of the Harvard Six Cities Study and the American Cancer Society Study of Particulate Air Pollution and Mortality. Res Rep Health Eff Inst. 2000.

21. Eum K-D, Kazemiparkouhi F, Wang B, Manjourides J, Pun V, Pavlu V, et al. Long-term NO2 exposures and cause-specific mortality in American older adults. Environ Int. 2019;124: 10–15. doi:10.1016/j.envint.2018.12.060

22. Lefler JS, Higbee JD, Burnett RT, Ezzati M, Coleman NC, Mann DD, et al. Air pollution and mortality in a large, representative U.S. cohort: multiple-pollutant analyses, and spatial and temporal decompositions. Environ Health. 2019;18: 101. doi:10.1186/s12940-019-0544-9

23. Hart JE, Rimm EB, Rexrode KM, Laden F. Changes in Traffic Exposure and the Risk of Incident Myocardial Infarction and All-Cause Mortality: Epidemiology. 2013;24: 734–742. doi:10.1097/EDE.0b013e31829d5dae

24. Lim CC, Hayes RB, Ahn J, Shao Y, Silverman DT, Jones RR, et al. Mediterranean Diet and the Association Between Air Pollution and Cardiovascular Disease Mortality Risk. Circulation. 2019;139: 1766–1775. doi:10.1161/CIRCULATIONAHA.118.035742

25. Lim CC, Hayes RB, Ahn J, Shao Y, Silverman DT, Jones RR, et al. Long-Term Exposure to Ozone and Cause-Specific Mortality Risk in the United States. Am J Respir Crit Care Med. 2019;200: 1022–1031. doi:10.1164/rccm.201806-1161OC

26. Lim CC, Hayes RB, Ahn J, Shao Y, Silverman DT, Jones RR, et al. Association between long-term exposure to ambient air pollution and diabetes mortality in the US. Environ Res. 2018;165: 330–336. doi:10.1016/j.envres.2018.04.011

27. Hart JE, Garshick E, Dockery DW, Smith TJ, Ryan L, Laden F. Long-Term Ambient Multipollutant Exposures and Mortality. Am J Respir Crit Care Med. 2011;183: 73–78. doi:10.1164/rccm.200912-1903OC

28. Lipfert FW, Baty JD, Miller JP, Wyzga RE. $PM_{2.5}$ Constituents and Related Air Quality Variables As Predictors of Survival in a Cohort of U.S. Military Veterans. Inhal Toxicol. 2006;18: 645–657. doi:10.1080/08958370600742946

29. Lipfert FW, Wyzga RE, Baty JD, Miller JP. Traffic density as a surrogate measure of environmental exposures in studies of air pollution health effects: Long-term mortality in a cohort of US veterans. Atmos Environ. 2006;40: 154–169. doi:10.1016/j.atmosenv.2005.09.027

30. Lipfert FW, Wyzga RE, Baty JD, Miller JP. Air Pollution and Survival within the Washington University– EPRI Veterans Cohort: Risks Based on Modeled Estimates of Ambient Levels of Hazardous and Criteria Air Pollutants. J Air Waste Manag Assoc. 2009;59: 473–489. doi:10.3155/1047-3289.59.4.473

31. Lipfert FW, Wyzga RE. Environmental predictors of survival in a cohort of U.S. military veterans: A multi-level spatio-temporal analysis stratified by race. Environ Res. 2019; 108842. doi:10.1016/j.envres.2019.108842

32. Lipfert FW, Wyzga RE. Revisiting the Veterans Cohort Mortality Study: New results and synthesis. J Air Waste Manag Assoc. 2018;68: 1248–1268. doi:10.1080/10962247.2018.1498409

33. Bauleo L, Bucci S, Antonucci C, Sozzi R, Davoli M, Forastiere F, et al. Long-term exposure to air pollutants from multiple sources and mortality in an industrial area: a cohort study. Occup Environ Med. 2019;76: 48–57. doi:10.1136/oemed-2018-105059

34. Carey IM, Atkinson RW, Kent AJ, van Staa T, Cook DG, Anderson HR. Mortality Associations with Long-Term Exposure to Outdoor Air Pollution in a National English Cohort. Am J Respir Crit Care Med. 2013;187: 1226–1233. doi:10.1164/rccm.201210-1758OC

35. Sifaki-Pistolla D, Lionis C, Koinis F, Georgoulias V, Tzanakis N. Lung cancer and annual mean exposure to outdoor air pollution in Crete, Greece: Eur J Cancer Prev. 2017;26: S208–S214. doi:10.1097/CEJ.0000000000000407

36. Raaschou-Nielsen O, Sørensen M, Ketzel M, Hertel O, Loft S, Tjønneland A, et al. Long-term exposure to traffic-related air pollution and diabetes-associated mortality: a cohort study. Diabetologia. 2012;56: 36–46. doi:10.1007/s00125-012-2698-7

37. Raaschou-Nielsen O, Andersen ZJ, Jensen SS, Ketzel M, Sørensen M, Hansen J, et al. Traffic air pollution and mortality from cardiovascular disease and all causes: a Danish cohort study. Environ Health. 2012;11: 60. doi:10.1186/1476-069X-11-60

38. Hvidtfeldt UA, Sørensen M, Geels C, Ketzel M, Khan J, Tjønneland A, et al. Long-term residential exposure to PM2.5, PM10, black carbon, NO2, and ozone and mortality in a Danish cohort. Environ Int. 2019;123: 265–272. doi:10.1016/j.envint.2018.12.010

39. Sørensen M, Lühdorf P, Ketzel M, Andersen ZJ, Tjønneland A, Overvad K, et al. Combined effects of road traffic noise and ambient air pollution in relation to risk for stroke? Environ Res. 2014;133: 49–55. doi:10.1016/j.envres.2014.05.011

40. Andersen ZJ, Kristiansen LC, Andersen KK, Olsen TS, Hvidberg M, Jensen SS, et al. Stroke and Long-Term Exposure to Outdoor Air Pollution From Nitrogen Dioxide: A Cohort Study. Stroke. 2012;43: 320–325. doi:10.1161/STROKEAHA.111.629246

41. Fischer PH, Marra M, Ameling CB, Hoek G, Beelen R, de Hoogh K, et al. Air Pollution and Mortality in Seven Million Adults: The Dutch Environmental Longitudinal Study (DUELS). Environ Health Perspect. 2015;123: 697–704. doi:10.1289/ehp.1408254

42. Dimakopoulou K, Samoli E, Beelen R, Stafoggia M, Andersen ZJ, Hoffmann B, et al. Air Pollution and Nonmalignant Respiratory Mortality in 16 Cohorts within the ESCAPE Project. Am J Respir Crit Care Med. 2014;189: 684–696. doi:10.1164/rccm.201310-1777OC

43. Beelen R, Raaschou-Nielsen O, Stafoggia M, Andersen ZJ, Weinmayr G, Hoffmann B, et al. Effects of long-term exposure to air pollution on natural-cause mortality: an analysis of 22 European cohorts within the multicentre ESCAPE project. The Lancet. 2014;383: 785–795. doi:10.1016/S0140-6736(13)62158-3

44. Beelen R, Stafoggia M, Raaschou-Nielsen O, Andersen ZJ, Xun WW, Katsouyanni K, et al. Long-term Exposure to Air Pollution and Cardiovascular Mortality: An Analysis of 22 European Cohorts. Epidemiology. 2014;25: 368–378. doi:10.1097/EDE.0000000000000076

45. Bentayeb M, Wagner V, Stempfelet M, Zins M, Goldberg M, Pascal M, et al. Association between long-term exposure to air pollution and mortality in France: A 25-year follow-up study. Environ Int. 2015;85: 5–14. doi:10.1016/j.envint.2015.08.006

46. Tonne C, Wilkinson P. Long-term exposure to air pollution is associated with survival following acute coronary syndrome. Eur Heart J. 2013;34: 1306–1311. doi:10.1093/eurheartj/ehs480

47. Tonne C, Halonen JI, Beevers SD, Dajnak D, Gulliver J, Kelly FJ, et al. Long-term traffic air and noise pollution in relation to mortality and hospital readmission among myocardial infarction survivors. Int J Hyg Environ Health. 2016;219: 72–78. doi:10.1016/j.ijheh.2015.09.003

48. Stockfelt L, Andersson EM, Molnár P, Rosengren A, Wilhelmsen L, Sallsten G, et al. Long term effects of residential NOx exposure on total and cause-specific mortality and incidence of myocardial infarction in a Swedish cohort. Environ Res. 2015;142: 197–206. doi:10.1016/j.envres.2015.06.045

49. Dehbi H-M, Blangiardo M, Gulliver J, Fecht D, de Hoogh K, Al-Kanaani Z, et al. Air pollution and cardiovascular mortality with over 25 years follow-up: A combined analysis of two British cohorts. Environ Int. 2017;99: 275–281. doi:10.1016/j.envint.2016.12.004

50. Beelen R, Hoek G, Houthuijs D, van den Brandt PA, Goldbohm RA, Fischer P, et al. The joint association of air pollution and noise from road traffic with cardiovascular mortality in a cohort study. Occup Environ Med. 2008;66: 243–250. doi:10.1136/oem.2008.042358

51. Beelen R, Hoek G, van den Brandt PA, Goldbohm RA, Fischer P, Schouten LJ, et al. Long-Term Effects of Traffic-Related Air Pollution on Mortality in a Dutch Cohort (NLCS-AIR Study). Environ Health Perspect. 2008;116: 196–202. doi:10.1289/ehp.10767

52. Brunekreef B, Beelen R, Hoek G, Schouten L, Bausch-Goldbohm S, Fischer P, et al. Effects of Long-Term Exposure to Traffic-Related Air Pollution on Respiratory and Cardiovascular Mortality in the Netherlands: The NLCS-AIR Study. Res Rep Health Eff Inst. 2009;139.

53. Hoek G, Brunekreef B, Goldbohm S, Fischer P, van den Brandt PA. Association between mortality and indicators of traffic-related air pollution in the Netherlands: a cohort study. The Lancet. 2002;360: 1203–1209. doi:10.1016/S0140-6736(02)11280-3

54. Nafstad P, Håheim LL, Wisløff T, Gram F, Oftedal B, Holme I, et al. Urban air pollution and mortality in a cohort of Norwegian men. Environ Health Perspect. 2004;112: 610–615. doi:10.1289/ehp.6684

55. Næss Ø, Nafstad P, Aamodt G, Claussen B, Rosland P. Relation between Concentration of Air Pollution and Cause-Specific Mortality: Four-Year Exposures to Nitrogen Dioxide and Particulate Matter Pollutants in 470 Neighborhoods in Oslo, Norway. Am J Epidemiol. 2007;165: 435–443. doi:10.1093/aje/kwk016

56. Filleul L. Twenty five year mortality and air pollution: results from the French PAARC survey. Occup Environ Med. 2005;62: 453–460. doi:10.1136/oem.2004.014746

57. Klompmaker JO, Hoek G, Bloemsma LD, Marra M, Wijga AH, van den Brink C, et al. Surrounding green, air pollution, traffic noise exposure and non-accidental and cause-specific mortality. Environ Int. 2020;134: 105341. doi:10.1016/j.envint.2019.105341

58. Cesaroni G, Porta D, Badaloni C, Stafoggia M, Eeftens M, Meliefste K, et al. Nitrogen dioxide levels estimated from land use regression models several years apart and association with mortality in a large cohort study. Environ Health. 2012;11: 48. doi:10.1186/1476-069X-11-48

59. Cesaroni G, Badaloni C, Gariazzo C, Stafoggia M, Sozzi R, Davoli M, et al. Long-Term Exposure to Urban Air Pollution and Mortality in a Cohort of More than a Million Adults in Rome. Environ Health Perspect. 2013;121: 324–331. doi:10.1289/ehp.1205862

60. Rosenlund M, Picciotto S, Forastiere F, Stafoggia M, Perucci CA. Traffic-Related Air Pollution in Relation to Incidence and Prognosis of Coronary Heart Disease. Epidemiology. 2008;19: 121–128. doi:10.1097/EDE.0b013e31815c1921

61. Schikowski T, Sugiri D, Ranft U, Gehring U, Heinrich J, Wichmann H-E, et al. Does respiratory health contribute to the effects of long-term air pollution exposure on cardiovascular mortality? Respir Res. 2007;8: 20. doi:10.1186/1465-9921-8-20

62. Gehring U, Heinrich J, Kramer U, Grote V, Hochadel M, Sugiri D, et al. Long-Term Exposure to Ambient Air Pollution and Cardiopulmonary Mortality in Women: Epidemiology. 2006;17: 545–551. doi:10.1097/01.ede.0000224541.38258.87

63. Heinrich J, Thiering E, Rzehak P, Krämer U, Hochadel M, Rauchfuss KM, et al. Long-term exposure to NO_2 and PM_{10} and all-cause and cause-specific mortality in a prospective cohort of women. Occup Environ Med. 2013;70: 179–186. doi:10.1136/oemed-2012-100876

64. Nieuwenhuijsen M, Gascon M, Martinez D, Ponjoan A, Blanch J, Garcia-Gil M, et al. Air Pollution, Noise, Blue Space, and Green Space and Premature Mortality in Barcelona: A Mega Cohort. Int J Environ Res Public Health. 2018;15: 2405. doi:10.3390/ijerph15112405

65. Maheswaran R, Pearson T, Smeeton NC, Beevers SD, Campbell MJ, Wolfe CD. Impact of Outdoor Air Pollution on Survival After Stroke: Population-Based Cohort Study. Stroke. 2010;41: 869–877. doi:10.1161/STROKEAHA.109.567743

66. Desikan A, Crichton S, Hoang U, Barratt B, Beevers SD, Kelly FJ, et al. Effect of Exhaust- and Nonexhaust-Related Components of Particulate Matter on Long-Term Survival After Stroke. Stroke. 2016;47: 2916–2922. doi:10.1161/STROKEAHA.116.014242

67. Héritier H, Vienneau D, Foraster M, Eze IC, Schaffner E, de Hoogh K, et al. A systematic analysis of mutual effects of transportation noise and air pollution exposure on myocardial infarction mortality: a nationwide cohort study in Switzerland. Eur Heart J. 2019;40: 598–603. doi:10.1093/eurheartj/ehy650

68. Cao J, Yang C, Li J, Chen R, Chen B, Gu D, et al. Association between long-term exposure to outdoor air pollution and mortality in China: A cohort study. J Hazard Mater. 2011;186: 1594–1600. doi:10.1016/j.jhazmat.2010.12.036

69. Dirgawati M, Hinwood A, Nedkoff L, Hankey GJ, Yeap BB, Flicker L, et al. Long-term Exposure to Low Air Pollutant Concentrations and the Relationship with All-Cause Mortality and Stroke in Older Men: Epidemiology. 2019;30: S82–S89. doi:10.1097/EDE.0000000000001034

70. Yang Y, Tang R, Qiu H, Lai P-C, Wong P, Thach T-Q, et al. Long term exposure to air pollution and mortality in an elderly cohort in Hong Kong. Environ Int. 2018;117: 99–106. doi:10.1016/j.envint.2018.04.034

71. Barratt B, Lee M, Wong P, Tang R. A Dynamic Three-Dimensional Air Pollution Exposure Model for Hong Kong. Res Rep Health Eff Inst. 2018;194.

72. Kim H, Kim J, Kim S, Kang S, Kim H, Kim H, et al. Cardiovascular Effects of Long-Term Exposure to Air Pollution: A Population-Based Study With 900 845 Person-Years of Follow-up. J Am Heart Assoc. 2017;6. doi:10.1161/JAHA.117.007170

73. Chen X, Zhang L, Huang J, Song F, Zhang L, Qian Z, et al. Long-term exposure to urban air pollution and lung cancer mortality: A 12-year cohort study in Northern China. Sci Total Environ. 2016;571: 855–861. doi:10.1016/j.scitotenv.2016.07.064

74. Yorifuji T, Kashima S, Tsuda T, Takao S, Suzuki E, Doi H, et al. Long-term exposure to traffic-related air pollution and mortality in Shizuoka, Japan. Occup Environ Med. 2010;67: 111–117. doi:10.1136/oem.2008.045542

75. Yorifuji T, Kashima S, Tsuda T, Ishikawa-Takata K, Ohta T, Tsuruta K, et al. Long-term exposure to traffic-related air pollution and the risk of death from hemorrhagic stroke and lung cancer in Shizuoka, Japan. Sci Total Environ. 2013;443: 397–402. doi:10.1016/j.scitotenv.2012.10.088

76. Dong G-H, Zhang P, Sun B, Zhang L, Chen X, Ma N, et al. Long-Term Exposure to Ambient Air Pollution and Respiratory Disease Mortality in Shenyang, China: A 12-Year Population-Based Retrospective Cohort Study. Respiration. 2012;84: 360–368. doi:10.1159/000332930

77. Zhang P, Dong G, Sun B, Zhang L, Chen X, Ma N, et al. Long-Term Exposure to Ambient Air Pollution and Mortality Due to Cardiovascular Disease and Cerebrovascular Disease in Shenyang, China. Federici M, editor. PLoS ONE. 2011;6: e20827. doi:10.1371/journal.pone.0020827

78. Tseng E, Ho W-C, Lin M-H, Cheng T-J, Chen P-C, Lin H-H. Chronic exposure to particulate matter and risk of cardiovascular mortality: cohort study from Taiwan. BMC Public Health. 2015;15: 936. doi:10.1186/s12889-015-2272-6

79. Katanoda K, Sobue T, Satoh H, Tajima K, Suzuki T, Nakatsuka H, et al. An Association Between Long-Term Exposure to Ambient Air Pollution and Mortality From Lung Cancer and Respiratory Diseases in Japan. J Epidemiol. 2011;21: 132–143. doi:10.2188/jea.JE20100098
