# Supplementary material for: Systematic review and meta-analysis of cohort studies of long term outdoor nitrogen dioxide exposure and mortality
Source: PLoS One. 2021 Feb 4;16(2):e0246451. doi: 10.1371/journal.pone.0246451 (PMC7861378; doi:10.1371/journal.pone.0246451)
Supplement: S5 Table — (PDF) [file pone.0246451.s013.pdf]

Online supplementary table S5. Reasons for assigned ratings of risk of bias greater than low risk (or unable to assess)

Study [reference]	Region	Cohort	Selection Bias	Exposure Assessment	Confounding	Completeness of Outcome Data	Conflict of Interest
Crouse 2015 [1]	Canada	CanCHEC 1991		did not account for exposure other than at places of residence	indirect adjustment for smoking		
Crouse 2015 [2]	Canada	CanCHEC 1991 (10 cities)		did not account for exposure other than at places of residence	indirect adjustment for smoking		
Weichenthal 2017 [3]	Canada	CanCHEC 2001		did not account for exposure other than at places of residence	no adjustment for smoking		
Villeneuve 2013 [4]	Canada	OTCS		single address	indirect adjustment for smoking		
Chen 2013 [5]	Canada	OTCS		did not account for exposure other than at places of residence	indirect adjustment for smoking		
Jerrett 2009 [6]	Canada	Toronto Western Hospital		single address			
Gan 2011 [7]	Canada	Vancouver CHD		did not account for exposure other than at places of residence	no adjustment for smoking but smoking not associated with exposure in related dataset		
Gan 2013 [8]	Canada	Vancouver COPD		did not account for exposure other than at places of residence	no adjustment for smoking but smoking not associated with exposure in related dataset		
Abbey 1999 [9]	United States	AHSMOG	may not be representative	monitoring sites up to 50 km from residence			
Chen 2005 [10]	United States	AHSMOG	may not be representative	monitoring sites up to 50 km from residence			

Study [reference]	Region	Cohort	Selection Bias	Exposure Assessment	Confounding	Completeness of Outcome Data	Conflict of Interest
Eckel 2016 [11]	United States	California cancer		single address	no adjustment for smoking		
Hartiala 2016 [12]	United States	Cleveland Clinic GeneBank	may not be representative	monitoring sites up to 50 km from residence and only 4 monitors for state			
Jerrett 2013 [13]	United States	CPS II	may not be representative	single address			
Pope 2002 [14]	United States	CPS II	may not be representative	area level only			
Krewski 2009 [15]	United States	CPS II	may not be representative	area level only			
McKean-Cowdin 2009 [16]	United States	CPS II	may not be representative	area level only			
Turner 2017 [17]	United States	CPS II	may not be representative	single address			
Turner 2016 [18]	United States	CPS II	may not be representative	single address			
Lipsett 2011 [19]	United States	CTS	may not be representative	did not account for exposure other than at places of residence			
Krewski 2000 [20]	United States	Harvard Six Cities	population based, but response rate unknown	single monitor per community			
Eum 2019 [21]	United States	Medicare		single address	no adjustment for smoking or SES but similar results in subset with area level smoking, SES, other covariate data		funded by Electric Power Research Institute (EPRI) but authors associated with academic institutions
Lefler 2019 [22]	United States	NHIS	population based, but response rate unknown	single address			

Study [reference]	Region	Cohort	Selection Bias	Exposure Assessment	Confounding	Completeness of Outcome Data	Conflict of Interest
Hart 2013 [23]	United States	NHS	may not be representative	did not account for exposure other than at places of residence			
Lim 2019 [24]	United States	NIH-AARP	may not be representative	single address			
Lim 2019 [25]	United States	NIH-AARP	may not be representative	single address			
Lim 2018 [26]	United States	NIH-AARP	may not be representative	single address			
Hart 2011 [27]	United States	TrIPS	may not be representative	single address	no adjustment for SES or smoking		
Lipfert 2006 [28]	United States	WU/EPRI Veterans	may not be representative	area level only			funded by EPRI but 1 author associated with academic institution
Lipfert 2006 [29]	United States	WU/EPRI Veterans	may not be representative	area level only			funded by EPRI but 1 author associated with academic institution
Lipfert 2009 [30]	United States	WU/EPRI Veterans	may not be representative	model performance not reported			funded by EPRI but 1 author associated with academic institution
Lipfert 2019 [31]	United States	WU/EPRI Veterans	may not be representative	area level only			funded by EPRI but analysis conducted at academic institution
Lipfert 2018 [32]	United States	WU/EPRI Veterans	may not be representative	area level only			funded by EPRI but analysis conducted at academic institution
Bauleo 2019 [33]	Europe	Civitavecchia		model performance not reported	no adjustment for smoking		
Carey 2013 [34]	Europe	CPRD	may not be representative	single address			
Sifaki-Pistolla 2017 [35]	Europe	Crete lung cancer		single address	no adjustment for SES		

Study [reference]	Region	Cohort	Selection Bias	Exposure Assessment	Confounding	Completeness of Outcome Data	Conflict of Interest
Raaschou-Nielsen 2012 [36]	Europe	DDCH	may not be representative	did not account for exposure other than at places of residence			
Raaschou-Nielsen 2012 [37]	Europe	DDCH	may not be representative	did not account for exposure other than at places of residence			
Hvidtfeldt 2019 [38]	Europe	DDCH	may not be representative	did not account for exposure other than at places of residence			
Sørensen 2014 [39]	Europe	DDCH	may not be representative	did not account for exposure other than at places of residence			
Andersen 2012 [40]	Europe	DDCH	may not be representative	did not account for exposure other than at places of residence			
Fischer 2015 [41]	Europe	DUELS		single address	no adjustment for smoking but not associated with exposure in related dataset		
Dimakopoulou 2014 [42]	Europe	ESCAPE	may not be representative	single address but similar results when restricted to non-movers			
Beelen 2014 [43]	Europe	ESCAPE	may not be representative	single address but similar results when restricted to non-movers			
Beelen 2014 [44]	Europe	ESCAPE	may not be representative	single address but similar results when restricted to non-movers			

Study [reference]	Region	Cohort	Selection Bias	Exposure Assessment	Confounding	Completeness of Outcome Data	Conflict of Interest
Bentayeb 2015 [45]	Europe	GAZEL	may not be representative	did not account for exposure other than at places of residence			
Tonne 2013 [46]	Europe	MINAP		single address, but of 25% of those with multiple admissions who moved, 90% changed postcode centroid by ≤300 m			
Tonne 2016 [47]	Europe	MINAP		multiple but not all addresses; only 5% moved of those with multiple admissions			
Stockfelt 2015 [48]	Europe	MPPS	may not be representative	did not account for exposure other than at places of residence			
Dehbi 2017 [49]	Europe	NHSD, SABRE	may not be representative	single address			
Beelen 2008 [50]	Europe	NLCS	may not be representative	single address but 70% of those who died did not move			
Beelen 2008 [51]	Europe	NLCS	may not be representative	single address but 70% of those who died did not move			
Brunekreef 2009 [52]	Europe	NLCS	may not be representative	single address but 70% of those who died did not move			
Hoek 2002 [53]	Europe	NLCS	may not be representative	did not account for exposure other than at places of residence			

Study [reference]	Region	Cohort	Selection Bias	Exposure Assessment	Confounding	Completeness of Outcome Data	Conflict of Interest
Nafstad 2004 [54]	Europe	Norwegian men	may not be representative	did not account for exposure other than at places of residence			
Naess 2007 [55]	Europe	Oslo		did not account for exposure other than at places of residence	no adjustment for smoking but smoking not associated with NO2 exposure in related data		
Filleul 2005 [56]	Europe	PAARC	may not be representative	single address			
Klompmaker 2020 [57]	Europe	PHM	may not be representative	single address			
Cesaroni 2012 [58]	Europe	RoLS		did not account for exposure other than at places of residence; non-movers only	no adjustment for smoking		
Cesaroni 2013 [59]	Europe	RoLS		did not account for exposure other than at places of residence	no adjustment for smoking but smoking not associated with exposure in related dataset and adjusted for chronic conditions associated with smoking		
Rosenlund 2008 [60]	Europe	Rome		area level only	no adjustment for smoking but adjusted for smoking-related co-morbidity and smoking not associated with NO2 exposure in related data		

Study [reference]	Region	Cohort	Selection Bias	Exposure Assessment	Confounding	Completeness of Outcome Data	Conflict of Interest
Schikowski 2007 [61]	Europe	SALIA	may not be representative	1 community had no monitoring site			
Gehring 2006 [62]	Europe	SALIA	may not be representative	1 community had no monitoring site			
Heinrich 2013 [63]	Europe	SALIA	may not be representative	1 community had no monitoring site			
Nieuwenhuijsen 2018 [64]				did not account for exposure other than at places of residence			
Maheswaran 2010 [65]	Europe	SLSR	may not be representative	did not account for exposure other than at places of residence			
Desikan et al. 2016 [66]	Europe	SLSR	may not be representative	single address	no adjustment for smoking		
Heritier 2019 [67]	Europe	SNC		single address	no adjustment for smoking		
Cao 2011 [68]	Other	CNHS	may not be representative	single address			
Dirgawati 2019 [69]	Other	HIMS	may not be representative	did not account for exposure other than at places of residence			
Yang 2018 [70]	Other	HKEHC	may not be representative	model R2=0.46, RMSE=28 µg/m3			
Barratt 2018 [71]	Other	HKEHC	may not be representative				
Kim 2017 [72]	Other	NHIS-NSC		place of residence mapped to nearest monitor but no distance criterion	not adjusted for smoking		
Chen 2016 [73]	Other	Northern Chinese	may not be representative	did not account for exposure other than at places of residence		~20% attrition but little difference by city	

Study [reference]	Region	Cohort	Selection Bias	Exposure Assessment	Confounding	Completeness of Outcome Data	Conflict of Interest
Yorifuji 2010 [74]	Other	SEC	may not be representative	model R2=0.54		27% lost to follow-up over 7 years	
Yorifuji 2013 [75]	Other	SEC	may not be representative	model R2=0.54		57% lost to follow-up over 10 years	
Dong 2012 [76]	Other	Shenyang	may not be representative	area level only	all important confounders but data based on retrospective interview which could have been affected by recall bias		
Zhang 2011 [77]	Other	Shenyang	may not be representative	area level only	all important confounders but data based on retrospective interview which could have been affected by recall bias		
Tseng 2015 [78]	Other	Taiwan civil servants	may not be representative	area level only			
Katanoda 2011 [79]	Other	Three Prefecture	may not be representative	area level, single monitor per community, at 2 sites >10 km from city centre			
