# Supplementary material for: Systematic review and meta-analysis of cohort studies of long term outdoor nitrogen dioxide exposure and mortality
Source: PLoS One. 2021 Feb 4;16(2):e0246451. doi: 10.1371/journal.pone.0246451 (PMC7861378; doi:10.1371/journal.pone.0246451)
Supplement: S6 Table — (PDF) [file pone.0246451.s014.pdf]

Online supplementary table S6. Leave one out analyses

Omitted Study	Hazard Ratio	Lower 95% Confidence Interval	Upper 95% Confidence Interval	Q	p(Q)	I^2
			All/natural cause			
Chen 2016 4 Chinese cities AC	1.050	1.027	1.075	144.6	0.000	95.8
Turner 2016 United States AC	1.048	1.022	1.075	425.1	0.000	96.9
Bentayeb 2015 France NC	1.046	1.021	1.071	424.1	0.000	96.8
Stockfelt 2015 Gothenburg, Sweden NC	1.046	1.021	1.072	423.2	0.000	96.9
Fischer 2015 Netherlands NC	1.047	1.021	1.074	396.6	0.000	96.5
Hart 2013 United States NC	1.049	1.023	1.075	425.8	0.000	97.0
Carey 2013 England AC	1.048	1.022	1.074	425.2	0.000	97.1
Tonne 2013 England and Wales AC	1.049	1.023	1.075	425.8	0.000	97.0
Cesaroni 2013 Rome, Italy NC	1.047	1.021	1.074	396.6	0.000	96.5
Yorifuji 2013 Shizuoka, Japan AC age 65+	1.041	1.019	1.063	411.2	0.000	95.6
Lipsett 2011 California AC	1.050	1.025	1.075	422.5	0.000	96.8
Cao 2011 China AC	1.047	1.021	1.073	424.0	0.000	97.0
Maheswaran 2010 London, UK AC	1.044	1.020	1.068	416.2	0.000	96.4
Jerrett 2009 Toronto, Canada NC	1.046	1.021	1.071	422.6	0.000	96.7
Rosenlund 2008 Rome, Italy AC	1.050	1.025	1.075	422.1	0.000	96.7
Filleul 2005 7 French cities NC	1.050	1.027	1.074	416.1	0.000	96.3
Nafstad 2004 Oslo, Norway NC male	1.036	1.020	1.053	391.0	0.000	91.9
Abbey 1999 California NC female	1.049	1.024	1.076	424.3	0.000	97.0
Abbey 1999 California NC male	1.049	1.023	1.075	425.7	0.000	97.0
Crouse 2015 Canada NC	1.047	1.021	1.074	396.3	0.000	96.8
Krewski 2000 6 US cities AC	1.044	1.020	1.069	421.3	0.000	96.6
Beelen 2014 Europe NC	1.049	1.023	1.076	425.5	0.000	97.0
Beelen 2008 Netherlands NC	1.048	1.022	1.074	425.5	0.000	97.1
Barratt 2018 Hong Kong NC age 65+	1.048	1.022	1.075	425.8	0.000	96.9
Dirgawati 2019 Perth, Australia AC male age 65+	1.046	1.021	1.071	424.3	0.000	96.9
Klompmaker 2020 Netherlands NC	1.050	1.025	1.076	420.9	0.000	96.8
Lefler 2019 United States AC	1.049	1.023	1.076	422.0	0.000	96.9
Lim 2019 6 US states NC	1.048	1.022	1.075	425.1	0.000	96.9
Lipfert 2019 United States AC male (black)	1.048	1.022	1.075	425.5	0.000	97.0
Lipfert 2019 United States AC male (white)	1.049	1.022	1.075	425.9	0.000	96.9
Nieuwenhuijsen 2018 Barcelona, Spain AC	1.048	1.022	1.075	425.7	0.000	97.0
Hartiala 2016 Ohio AC	1.047	1.023	1.073	426.0	0.000	96.8

Omitted Study	Hazard Ratio	Lower 95% Confidence Interval	Upper 95% Confidence Interval	Q	p(Q)	I^2
	Cardiovascular					
Dehbi 2017 London, UK	1.059	1.027	1.093	118.3	0.000	93.2
Turner 2016 United States	1.058	1.022	1.094	105.1	0.000	93.0
Bentayeb 2015 France	1.059	1.026	1.092	118.6	0.000	93.1
Stockfelt 2015 Gothenburg, Sweden	1.058	1.024	1.093	118.1	0.000	93.6
Fischer 2015 Netherlands	1.063	1.029	1.098	93.9	0.000	92.4
Beelen 2014 Europe	1.061	1.026	1.096	118.3	0.000	93.7
Carey 2013 England	1.062	1.028	1.097	115.9	0.000	93.4
Cesaroni 2013 Rome, Italy	1.059	1.024	1.096	117.0	0.000	93.2
Chen 2013 3 Canadian cities	1.048	1.025	1.071	102.6	0.000	83.9
Yorifuji 2013 Shizuoka, Japan age 65+	1.047	1.026	1.067	92.0	0.000	80.5
Lipsett 2011 California	1.062	1.028	1.096	117.2	0.000	93.2
Cao 2011 China	1.057	1.023	1.092	116.6	0.000	93.5
Jerrett 2009 Toronto, Canada	1.056	1.025	1.089	112.5	0.000	92.8
Naess 2007 Oslo, Norway female age 51-70	1.059	1.024	1.095	118.4	0.000	93.9
Naess 2007 Oslo, Norway female age 71-90	1.062	1.028	1.098	106.5	0.000	92.9
Naess 2007 Oslo, Norway male age 51-70	1.058	1.023	1.095	117.2	0.000	93.8
Naess 2007 Oslo, Norway male age 71-90	1.062	1.027	1.098	113.9	0.000	93.3
Crouse 2015 Canada	1.060	1.024	1.096	117.7	0.000	93.0
Beelen 2008 Netherlands	1.059	1.025	1.095	118.7	0.000	93.8
Tseng 2015 Taiwan	1.059	1.027	1.093	118.3	0.000	93.1
Barratt 2018 Hong Kong age 65+	1.059	1.024	1.096	118.4	0.000	93.7
Klompmaker 2020 Netherlands	1.063	1.032	1.094	113.4	0.000	91.8
Lim 2019 6 US states	1.058	1.023	1.095	114.6	0.000	93.5

Omitted Study	Hazard Ratio	Lower 95% Confidence Interval	Upper 95% Confidence Interval	Q	p(Q)	I^2
	Lung cancer					
Chen 2016 4 Chinese cities	1.093	1.053	1.133	165.4	0.000	77.6
Turner 2016 United States	1.090	1.047	1.135	248.8	0.000	83.8
Fischer 2015 Netherlands	1.071	1.031	1.112	92.4	0.000	75.4
Carey 2013 England	1.082	1.038	1.128	274.0	0.000	86.2
Cesaroni 2013 Rome, Italy	1.084	1.039	1.131	273.6	0.000	85.7
Yorifuji 2013 Shizuoka, Japan age 65+	1.078	1.037	1.121	271.1	0.000	85.0
Lipsett 2011 California	1.084	1.042	1.129	273.7	0.000	85.9
Katanoda 2011 Japan	1.079	1.035	1.124	272.5	0.000	85.5
Cao 2011 China	1.082	1.038	1.126	274.0	0.000	86.0
Naess 2007 Oslo, Norway female age 51-70	1.076	1.034	1.120	270.9	0.000	84.7
Naess 2007 Oslo, Norway female age 71-90	1.082	1.038	1.128	274.1	0.000	86.2
Naess 2007 Oslo, Norway male age 51-70	1.085	1.040	1.131	273.6	0.000	86.3
Naess 2007 Oslo, Norway male age 71-90	1.084	1.039	1.130	274.0	0.000	86.4
Filleul 2005 7 French cities	1.086	1.043	1.130	272.7	0.000	85.8
Nafstad 2004 Oslo, Norway male	1.077	1.036	1.119	268.9	0.000	84.6
Abbey 1999 California female	1.079	1.038	1.122	270.6	0.000	85.2
Abbey 1999 California male	1.080	1.038	1.123	272.6	0.000	85.4
Crouse 2015 Canada	1.084	1.039	1.131	273.2	0.000	85.2
Krewski 2005 6 US cities	1.083	1.040	1.127	274.1	0.000	85.8
Beelen 2008 Netherlands	1.088	1.046	1.133	270.1	0.000	85.4
Hvitfeldt 2019 2 Danish cities	1.083	1.040	1.129	274.1	0.000	86.2
Klompmaker 2020 Netherlands	1.083	1.040	1.128	274.1	0.000	86.1
Lim 2019 6 US states	1.089	1.046	1.135	245.8	0.000	83.6

Omitted Study	Hazard Ratio	Lower 95% Confidence Interval	Upper 95% Confidence Interval	Q	p(Q)	I^2
	Respiratory					
Turner 2016 United States RES	1.065	1.036	1.095	65.2	0.000	67.1
Bentayeb 2015 France RES	1.062	1.035	1.090	65.8	0.000	67.1
Stockfelt 2015 Gothenburg, Sweden RES	1.062	1.035	1.090	66.2	0.000	67.3
Fischer 2015 Netherlands RES	1.065	1.035	1.095	63.4	0.000	62.2
Carey 2013 England RES	1.056	1.031	1.082	60.2	0.000	60.7
Gan 2013 Vancouver, Canada COPD	1.061	1.034	1.089	66.2	0.000	67.6
Cesaroni 2013 Rome, Italy RES	1.062	1.033	1.093	66.3	0.000	69.4
Yorifuji 2013 Shizuoka, Japan RES age 65+	1.058	1.032	1.085	60.3	0.000	64.0
Katanoda 2011 Japan RES	1.043	1.030	1.055	39.4	0.013	0.70
Cao 2011 China RES	1.060	1.032	1.088	65.4	0.000	67.3
Jerrett 2009 Toronto, Canada RES	1.062	1.035	1.089	66.3	0.000	66.9
Naess 2007 Oslo, Norway COPD female age 51-70	1.064	1.035	1.094	65.8	0.000	68.3
Naess 2007 Oslo, Norway COPD female age 71-90	1.062	1.033	1.092	66.3	0.000	69.1
Naess 2007 Oslo, Norway COPD male age 51-70	1.058	1.032	1.085	62.9	0.000	64.4
Naess 2007 Oslo, Norway COPD male age 71-90	1.063	1.035	1.093	66.2	0.000	68.8
Nafstad 2004 Oslo, Norway RES male	1.059	1.033	1.085	56.7	0.000	64.7
Abbey 1999 California RES female	1.064	1.037	1.092	65.2	0.000	67.1
Abbey 1999 California RES male	1.065	1.038	1.092	63.2	0.000	65.1
Crouse 2015 Canada RES	1.064	1.034	1.095	65.8	0.000	66.8
Beelen 2008 Netherlands RES	1.059	1.033	1.087	64.2	0.000	66.0
Barratt 2018 Hong Kong RES age 65+	1.064	1.035	1.095	65.5	0.000	67.1
Klompmaker 2020 Netherlands RES	1.063	1.036	1.091	65.3	0.000	67.1
Lim 2019 6 US states RES	1.064	1.034	1.094	66.1	0.000	68.4
Dimakopoulou 2014 Europe RES	1.064	1.037	1.092	64.5	0.000	66.4

Omitted Study	Hazard Ratio	Lower 95% Confidence Interval	Upper 95% Confidence Interval	Q	p(Q)	I^2
			Cerebrovascular			
Turner 2016 United States	1.022	1.002	1.043	17.3	0.099	0.2
Stockfelt 2015 Gothenburg, Sweden	1.014	0.996	1.032	18.9	0.063	0.5
Beelen 2014 Europe	1.014	0.996	1.032	19.7	0.050	0.1
Cesaroni 2013 Rome, Italy	1.014	0.993	1.035	19.6	0.051	2.9
Chen 2013 3 Canadian cities	1.015	0.997	1.033	19.5	0.053	0.0
Yorifuji 2013 Shizuoka, Japan age 65+	1.012	0.995	1.030	11.9	0.374	0.0
Lipsett 2011 California	1.015	0.998	1.034	17.2	0.102	0.1
Nafstad 2004 Oslo, Norway male	1.014	0.996	1.032	19.2	0.058	0.0
Crouse 2015 Canada	1.018	0.994	1.041	19.6	0.051	7.3
Beelen 2008 Netherlands	1.013	0.995	1.031	14.6	0.200	0.0
Barratt 2018 Hong Kong age 65+	1.011	0.992	1.030	18.8	0.065	0.0
Dirgawati 2019 Perth, Australia male age 65+	1.014	0.997	1.032	19.3	0.056	0.1
Lim 2019 6 US states	1.014	0.995	1.033	19.6	0.051	0.2
			Ischemic heart disease			
Turner 2016 United States IHD	1.091	1.077	1.106	32.2	0.001	0.0
Stockfelt 2015 Gothenburg, Sweden IHD	1.118	1.076	1.162	40.7	0.000	83.8
Beelen 2014 Europe IHD	1.117	1.081	1.154	39.5	0.000	77.1
Cesaroni 2013 Rome, Italy IHD	1.123	1.073	1.175	40.6	0.000	84.9
Chen 2013 3 Canadian cities IHD	1.100	1.079	1.122	30.6	0.002	38.0
Yorifuji 2013 Shizuoka, Japan IHD age 65+	1.104	1.079	1.130	32.5	0.001	54.2
Lipsett 2011 California IHD	1.118	1.077	1.160	40.6	0.000	82.2
Gan 2011 Vancouver, Canada IHD	1.121	1.074	1.170	40.7	0.000	86.3
Chen 2005 California IHD female	1.111	1.076	1.147	40.3	0.000	75.3
Nafstad 2004 Oslo, Norway IHD male	1.103	1.079	1.128	34.1	0.001	51.1
Crouse 2015 Canada IHD	1.124	1.075	1.174	36.0	0.000	81.4
Beelen 2008 Netherlands IHD	1.115	1.085	1.145	37.3	0.000	63.8
Barratt 2018 Hong Kong IHD age 65+	1.123	1.074	1.173	40.5	0.000	86.4
Lim 2019 6 US states IHD	1.123	1.074	1.174	40.3	0.000	85.4
