# Supplementary material for: Systematic review and meta-analysis of cohort studies of long term outdoor nitrogen dioxide exposure and mortality
Source: PLoS One. 2021 Feb 4;16(2):e0246451. doi: 10.1371/journal.pone.0246451 (PMC7861378; doi:10.1371/journal.pone.0246451)
Supplement: S1 Text — (PDF) [file pone.0246451.s015.pdf]

Online Supplementary File S1. Sensitivity to Estimator
REML = Restricted Maximum Likelihood
DL = Dersimonian and Laird
EB = Empirical Bayes

All/natural cause mortality

```
Random-Effects Model (k = 32; tau^2 estimator: REML)

tau^2 (estimated amount of total heterogeneity): 0.0036 (SE = 0.0011)
tau (square root of estimated tau^2 value):      0.0597
I^2 (total heterogeneity / total variability):   96.72%
H^2 (total variability / sampling variability):  30.47

Test for Heterogeneity:
Q(df = 31) = 425.9933, p-val < .0001

Model Results:

estimate      se    zval    pval   ci.lb   ci.ub
  0.0461  0.0121  3.8037  0.0001  0.0223  0.0699  ***

Signif. codes:  0 ‘***’ 0.001 ‘**’ 0.01 ‘*’ 0.05 ‘.’ 0.1 ‘ ’ 1

Random-Effects Model (k = 32; tau^2 estimator: DL)

tau^2 (estimated amount of total heterogeneity): 0.0015 (SE = 0.0007)
tau (square root of estimated tau^2 value):      0.0393
I^2 (total heterogeneity / total variability):   92.72%
H^2 (total variability / sampling variability):  13.74

Test for Heterogeneity:
Q(df = 31) = 425.9933, p-val < .0001

Model Results:

estimate      se    zval    pval   ci.lb   ci.ub
  0.0412  0.0086  4.8056  <.0001  0.0244  0.0580  ***

Signif. codes:  0 ‘***’ 0.001 ‘**’ 0.01 ‘*’ 0.05 ‘.’ 0.1 ‘ ’ 1

Random-Effects Model (k = 32; tau^2 estimator: EB)

tau^2 (estimated amount of total heterogeneity): 0.0067 (SE = 0.0021)
tau (square root of estimated tau^2 value):      0.0821
I^2 (total heterogeneity / total variability):   98.24%
H^2 (total variability / sampling variability):  56.72

Test for Heterogeneity:
Q(df = 31) = 425.9933, p-val < .0001

Model Results:

estimate      se    zval    pval   ci.lb   ci.ub
  0.0503  0.0160  3.1429  0.0017  0.0189  0.0817  **

Signif. codes:  0 ‘***’ 0.001 ‘**’ 0.01 ‘*’ 0.05 ‘.’ 0.1 ‘ ’ 1
```

Cardiovascular mortality

```
Random-Effects Model (k = 23; tau^2 estimator: REML)

tau^2 (estimated amount of total heterogeneity): 0.0038 (SE = 0.0016)
tau (square root of estimated tau^2 value):      0.0618
I^2 (total heterogeneity / total variability):   92.76%
H^2 (total variability / sampling variability):  13.80

Test for Heterogeneity:
Q(df = 22) = 118.6822, p-val < .0001

Model Results:

estimate      se    zval    pval   ci.lb   ci.ub
  0.0567  0.0157  3.6057  0.0003  0.0259  0.0875  ***


Signif. codes:  0 '***' 0.001 '**' 0.01 '*' 0.05 '.' 0.1 ' ' 1


Random-Effects Model (k = 23; tau^2 estimator: DL)

tau^2 (estimated amount of total heterogeneity): 0.0013 (SE = 0.0007)
tau (square root of estimated tau^2 value):      0.0362
I^2 (total heterogeneity / total variability):   81.46%
H^2 (total variability / sampling variability):  5.39

Test for Heterogeneity:
Q(df = 22) = 118.6822, p-val < .0001

Model Results:

estimate      se    zval    pval   ci.lb   ci.ub
  0.0519  0.0103  5.0281  <.0001  0.0317  0.0722  ***


Signif. codes:  0 '***' 0.001 '**' 0.01 '*' 0.05 '.' 0.1 ' ' 1


Random-Effects Model (k = 23; tau^2 estimator: EB)

tau^2 (estimated amount of total heterogeneity): 0.0085 (SE = 0.0034)
tau (square root of estimated tau^2 value):      0.0919
I^2 (total heterogeneity / total variability):   96.59%
H^2 (total variability / sampling variability):  29.34

Test for Heterogeneity:
Q(df = 22) = 118.6822, p-val < .0001

Model Results:

estimate      se    zval    pval   ci.lb   ci.ub
  0.0600  0.0221  2.7205  0.0065  0.0168  0.1032  **


Signif. codes:  0 '***' 0.001 '**' 0.01 '*' 0.05 '.' 0.1 ' ' 1
```

Lung cancer mortality

```
Random-Effects Model (k = 23; tau^2 estimator: REML)

tau^2 (estimated amount of total heterogeneity): 0.0052 (SE = 0.0025)
tau (square root of estimated tau^2 value):      0.0719
I^2 (total heterogeneity / total variability):   85.08%
H^2 (total variability / sampling variability):  6.70

Test for Heterogeneity:
Q(df = 22) = 274.1038, p-val < .0001

Model Results:

estimate      se    zval    pval   ci.lb   ci.ub
  0.0794  0.0201  3.9453  <.0001  0.0399  0.1188  ***


Signif. codes:  0 '***' 0.001 '**' 0.01 '*' 0.05 '.' 0.1 ' ' 1


Random-Effects Model (k = 23; tau^2 estimator: DL)

tau^2 (estimated amount of total heterogeneity): 0.0104 (SE = 0.0066)
tau (square root of estimated tau^2 value):      0.1019
I^2 (total heterogeneity / total variability):   91.97%
H^2 (total variability / sampling variability):  12.46

Test for Heterogeneity:
Q(df = 22) = 274.1038, p-val < .0001

Model Results:

estimate      se    zval    pval   ci.lb   ci.ub
  0.0860  0.0261  3.2920  0.0010  0.0348  0.1373  ***


Signif. codes:  0 '***' 0.001 '**' 0.01 '*' 0.05 '.' 0.1 ' ' 1


Random-Effects Model (k = 23; tau^2 estimator: EB)

tau^2 (estimated amount of total heterogeneity): 0.0068 (SE = 0.0034)
tau (square root of estimated tau^2 value):      0.0822
I^2 (total heterogeneity / total variability):   88.17%
H^2 (total variability / sampling variability):  8.45

Test for Heterogeneity:
Q(df = 22) = 274.1038, p-val < .0001

Model Results:

estimate      se    zval    pval   ci.lb   ci.ub
  0.0818  0.0222  3.6855  0.0002  0.0383  0.1253  ***


Signif. codes:  0 '***' 0.001 '**' 0.01 '*' 0.05 '.' 0.1 ' ' 1
```

Respiratory mortality

```
Random-Effects Model (k = 24; tau^2 estimator: REML)

tau^2 (estimated amount of total heterogeneity): 0.0017 (SE = 0.0010)
tau (square root of estimated tau^2 value):      0.0415
I^2 (total heterogeneity / total variability):   65.90%
H^2 (total variability / sampling variability):  2.93

Test for Heterogeneity:
Q(df = 23) = 66.3549, p-val < .0001

Model Results:

estimate      se    zval    pval   ci.lb   ci.ub
  0.0598  0.0131  4.5707  <.0001  0.0341  0.0854  ***

Signif. codes:  0 '***' 0.001 '**' 0.01 '*' 0.05 '.' 0.1 ' ' 1


Random-Effects Model (k = 24; tau^2 estimator: DL)

tau^2 (estimated amount of total heterogeneity): 0.0017 (SE = 0.0011)
tau (square root of estimated tau^2 value):      0.0410
I^2 (total heterogeneity / total variability):   65.34%
H^2 (total variability / sampling variability):  2.88

Test for Heterogeneity:
Q(df = 23) = 66.3549, p-val < .0001

Model Results:

estimate      se    zval    pval   ci.lb   ci.ub
  0.0597  0.0130  4.6019  <.0001  0.0343  0.0851  ***

Signif. codes:  0 '***' 0.001 '**' 0.01 '*' 0.05 '.' 0.1 ' ' 1

Random-Effects Model (k = 24; tau^2 estimator: EB)

tau^2 (estimated amount of total heterogeneity): 0.0055 (SE = 0.0027)
tau (square root of estimated tau^2 value):      0.0742
I^2 (total heterogeneity / total variability):   86.06%
H^2 (total variability / sampling variability):  7.18

Test for Heterogeneity:
Q(df = 23) = 66.3549, p-val < .0001

Model Results:

estimate      se    zval    pval   ci.lb   ci.ub
  0.0637  0.0196  3.2512  0.0011  0.0253  0.1020  **

Signif. codes:  0 '***' 0.001 '**' 0.01 '*' 0.05 '.' 0.1 ' ' 1
```

Cerebrovascular mortality

```
Random-Effects Model (k = 13; tau^2 estimator: REML)

tau^2 (estimated amount of total heterogeneity): 0.0000 (SE = 0.0003)
tau (square root of estimated tau^2 value):      0.0013
I^2 (total heterogeneity / total variability):   0.14%
H^2 (total variability / sampling variability):  1.00

Test for Heterogeneity:
Q(df = 12) = 19.6620, p-val = 0.0738

Model Results:

estimate      se    zval    pval    ci.lb   ci.ub
  0.0141  0.0090  1.5759  0.1151  -0.0034  0.0317

Signif. codes:  0 ‘***’ 0.001 ‘**’ 0.01 ‘*’ 0.05 ‘.’ 0.1 ‘ ’ 1

Random-Effects Model (k = 13; tau^2 estimator: DL)

tau^2 (estimated amount of total heterogeneity): 0.0008 (SE = 0.0009)
tau (square root of estimated tau^2 value):      0.0278
I^2 (total heterogeneity / total variability):   38.97%
H^2 (total variability / sampling variability):  1.64

Test for Heterogeneity:
Q(df = 12) = 19.6620, p-val = 0.0738

Model Results:

estimate      se    zval    pval    ci.lb   ci.ub
  0.0203  0.0147  1.3857  0.1658  -0.0084  0.0491

Signif. codes:  0 ‘***’ 0.001 ‘**’ 0.01 ‘*’ 0.05 ‘.’ 0.1 ‘ ’ 1

Random-Effects Model (k = 13; tau^2 estimator: EB)

tau^2 (estimated amount of total heterogeneity): 0.0049 (SE = 0.0039)
tau (square root of estimated tau^2 value):      0.0702
I^2 (total heterogeneity / total variability):   80.35%
H^2 (total variability / sampling variability):  5.09

Test for Heterogeneity:
Q(df = 12) = 19.6620, p-val = 0.0738

Model Results:

estimate      se    zval    pval    ci.lb   ci.ub
  0.0331  0.0272  1.2153  0.2242  -0.0203  0.0864

Signif. codes:  0 ‘***’ 0.001 ‘**’ 0.01 ‘*’ 0.05 ‘.’ 0.1 ‘ ’ 1
```

Ischemic heart disease mortality

```
Random-Effects Model (k = 14; tau^2 estimator: REML)

tau^2 (estimated amount of total heterogeneity): 0.0014 (SE = 0.0010)
tau (square root of estimated tau^2 value):      0.0373
I^2 (total heterogeneity / total variability):   69.90%
H^2 (total variability / sampling variability):  3.32

Test for Heterogeneity:
Q(df = 13) = 40.7351, p-val = 0.0001

Model Results:

estimate      se    zval    pval   ci.lb   ci.ub
  0.1056  0.0149  7.0671  <.0001  0.0763  0.1349  ***

Signif. codes:  0 ‘***’ 0.001 ‘**’ 0.01 ‘*’ 0.05 ‘.’ 0.1 ‘ ’ 1

Random-Effects Model (k = 14; tau^2 estimator: DL)

tau^2 (estimated amount of total heterogeneity): 0.0013 (SE = 0.0010)
tau (square root of estimated tau^2 value):      0.0357
I^2 (total heterogeneity / total variability):   68.09%
H^2 (total variability / sampling variability):  3.13

Test for Heterogeneity:
Q(df = 13) = 40.7351, p-val = 0.0001

Model Results:

estimate      se    zval    pval   ci.lb   ci.ub
  0.1051  0.0145  7.2381  <.0001  0.0767  0.1336  ***

Signif. codes:  0 ‘***’ 0.001 ‘**’ 0.01 ‘*’ 0.05 ‘.’ 0.1 ‘ ’ 1

Random-Effects Model (k = 14; tau^2 estimator: EB)

tau^2 (estimated amount of total heterogeneity): 0.0088 (SE = 0.0047)
tau (square root of estimated tau^2 value):      0.0938
I^2 (total heterogeneity / total variability):   93.63%
H^2 (total variability / sampling variability):  15.71

Test for Heterogeneity:
Q(df = 13) = 40.7351, p-val = 0.0001

Model Results:

estimate      se    zval    pval   ci.lb   ci.ub
  0.1193  0.0292  4.0780  <.0001  0.0619  0.1766  ***

Signif. codes:  0 ‘***’ 0.001 ‘**’ 0.01 ‘*’ 0.05 ‘.’ 0.1 ‘ ’ 1
```
